# The scale of zebrafish pectoral fin buds is determined by intercellular K^+^ levels and consequent Ca^2+^-mediated signaling via retinoic acid regulation of Rcan2 and Kcnk5b

**DOI:** 10.1371/journal.pbio.3002565

**Published:** 2024-03-25

**Authors:** Xiaowen Jiang, Kun Zhao, Yi Sun, Xinyue Song, Chao Yi, Tianlong Xiong, Sen Wang, Yi Yu, Xiduo Chen, Run Liu, Xin Yan, Christopher L. Antos

**Affiliations:** 1 School of Life Sciences and Technology, ShanghaiTech University, Shanghai, People’s Republic of China; 2 Center for Quantitative Biology, Peking University, Beijing, People’s Republic of China; 3 Institut für Pharmakologie und Toxikologie, Technische Universität Dresden, Dresden, Germany; University of Pennsylvania School of Medicine, UNITED STATES

## Abstract

K^+^ channels regulate morphogens to scale adult fins, but little is known about what regulates the channels and how they control morphogen expression. Using the zebrafish pectoral fin bud as a model for early vertebrate fin/limb development, we found that K^+^ channels also scale this anatomical structure, and we determined how one K^+^-leak channel, Kcnk5b, integrates into its developmental program. From FLIM measurements of a Förster Resonance Energy Transfer (FRET)-based K^+^ sensor, we observed coordinated decreases in intracellular K^+^ levels during bud growth, and overexpression of K^+^-leak channels in vivo coordinately increased bud proportions. Retinoic acid, which can enhance fin/limb bud growth, decreased K^+^ in bud tissues and up-regulated *regulator of calcineurin* (*rcan2*). *rcan2* overexpression increased bud growth and decreased K^+^, while CRISPR-Cas9 targeting of *rcan2* decreased growth and increased K^+^. We observed similar results in the adult caudal fins. Moreover, CRISPR targeting of Kcnk5b revealed that Rcan2-mediated growth was dependent on the Kcnk5b. We also found that Kcnk5b enhanced depolarization in fin bud cells via Na^+^ channels and that this enhanced depolarization was required for Kcnk5b-enhanced growth. Lastly, Kcnk5b-induced *shha* transcription and bud growth required IP_3_R-mediated Ca^2+^ release and CaMKK activity. Thus, we provide a mechanism for how retinoic acid via *rcan2* can regulate K^+^-channel activity to scale a vertebrate appendage via intercellular Ca^2+^ signaling.

## Abstract 2

Potassium channels regulate the scale of fish fins, but the underlying mechanisms remain unclear. These authors show that the scale of zebrafish embryonic pectoral fins is determined by the regulation of intracellular K+ by retinoic acid via Rcan2 and the potassium channel Kcnk5b.

## Introduction

The scaling of anatomical structures involves the coordinated control of gene transcription and intracellular communication. An increasing number of findings are linking electrophysiological changes to the regulation of developmental phenomena in different biological contexts [1,2]; as examples, transmembrane voltage potential influences eye development via regulation of Pax6 [3]; Hedgehog signaling is regulated in *Drosophila* wing discs by cell depolarization [4]; and inactivation of K^+^ inward rectifying channels leads to patterning defects in the craniofacial skeleton, vertebrate phalanges, and fly wings via disruption of BMP/Dpp signaling [5,6]. Furthermore, gain-of-function mutations in channels that facilitate outward flow of intracellular K^+^ are also linked to syndromes that generate craniofacial alterations, neurodevelopmental impairments, defects in the development of the limbs, etc. [7,8]. As discoveries for the involvement of electrophysiological regulation in development increase, much remains unknown about how these endogenous electrophysiological changes are integrated into the known molecular mechanisms that regulate development.

In zebrafish, mutations that increase the activity of K^+^ channels promote allometric growth of juvenile and adult fish fins [9–13], linking intracellular K^+^ in the control of coordinated proportional growth of these anatomical structures. The K^+^ concentration is high inside cells, so the opening of K^+^-leak channels generally causes an outward flow of K^+^ [14]. In the adult fin, increasing K^+^-leak channel activity increases the transcription of several morphogens and promotes growth [11]. However, it remains unknown how K^+^ channel activity at the cell membrane is controlled and relayed during the scaling process of fish appendages, and whether a similar electrophysiological control exists in the conserved embryonic vertebrate fin/limb bud developmental program.

Early embryonic limb and pectoral fin buds form at specific locations in the lateral plate mesoderm that expresses an evolutionary conserved profile of morphogens and growth factors [15]. The development of limb buds and pectoral fin buds initiate when retinoic acid (RA) indirectly promotes the distal transcription of Fgf10 [16], which in turn induces the expression of Fgf8 and the formation of the apical ectodermal ridge (AER) in the distal anterior–posterior interface of ectodermal cells of the buds [17,18]. After formation of the AER, the morphogen Sonic hedgehog (Shh) manifests in a group of bud mesenchymal cells known as a zone of polarizing activity (ZPA). The ZPA forms in the posterior bud mesenchyme and is required for growth and patterning through the activities of Shh [19,20]. Control of the AER and ZPA involve communication between these 2 regions, as well as other regional cell groups via their morphogens/growth factors (e.g., Bmp4) [21]. Similar to early limb bud development, activation of these signaling centers and other morphogens promote pectoral fin bud outgrowth [22]. For the zebrafish pectoral fin bud, growth is underway by 28 to30 hours post fertilization (hpf) [23]. During limb development, growth persist until the formation of the distal digits [24], but in zebrafish, pectoral fin bud development is limited to forming the endochondral bone, musculature, and other tissues at the proximal base of the fin [25,26]. Roughly 24 h after initiation of fin bud outgrowth, its development has transitioned into the development of a different type of appendage: the finfold, and many of the morphogen/growth factor signals have rearranged to the distal tip of the fin bud by 54 to 56 hpf to generate the finfold and then ultimately larval pectoral fins [23,27]. While the basic interactions between signaling centers and signaling molecules during early fin/limb bud development is understood, it is unknown whether changes in intracellular K^+^ will coordinately regulate them in a similar manner as it does during developmental/regenerative growth of fins.

Using a genetic sensor for K^+^ [28] with Fluorescence Lifetime Microscopy (FLIM), we found that relative levels of intracellular K^+^ decrease throughout the early pectoral fin bud during its growth. When we transgenically overexpressed K^+^-leak channels that decrease in intracellular K^+^, we increased bud growth and coordinately increased the expression several morphogens (*fgf8a*, *fgf10a*, *aldh1a2*, *shha*, and *bmp4*) that control bud development. Treatment with RA, a signal that can promote growth of vertebrate appendages, decreased intracellular K^+^ levels in the buds. We found that RA induces *rcan2* expression and that *rcan2* scales fin buds and decreases intracellular K^+^. In addition, we found that the K^+^-leak channel *kcnk5b* is expressed in pectoral fin buds and that it is required for Rcan2-mediated scaling. We also determined that Kcnk5b promotes depolarization and that depolarization is required for enhanced fin bud growth. We further show that Kcnk5b activity requires IP_3_R-mediated Ca^2+^ release and CaMKK activity for SHH transcription in vitro and in vivo as well as for kcnk5b’s enhancement of fin bud growth. Thus, we provide a mechanism through which an important proximal morphogen (RA) can regulate the activity of a K^+^-leak channel via Rcan2 to promote Ca^2+^-mediated scaling of embryonic pectoral fins buds.

## Results

### Endogenous intracellular K^+^ levels decrease during fin bud growth

We previously showed that Kcnk5b promotes allometric growth of adult fins via hierarchical activation of several morphogens [11]. The ability of an individual K^+^ channel to promote the expression of several developmental morphogens in the adult fin and larva led us to hypothesize that this phenomenon may be more broadly functional, and that it regulates the early developmental program conserved in vertebrate fin/limb buds, which is present in the developing pectoral fin buds of zebrafish [22]. Therefore, we wanted to determine whether there are endogenous changes in intracellular K^+^ in the developing pectoral fin bud.

**Fig 1:**
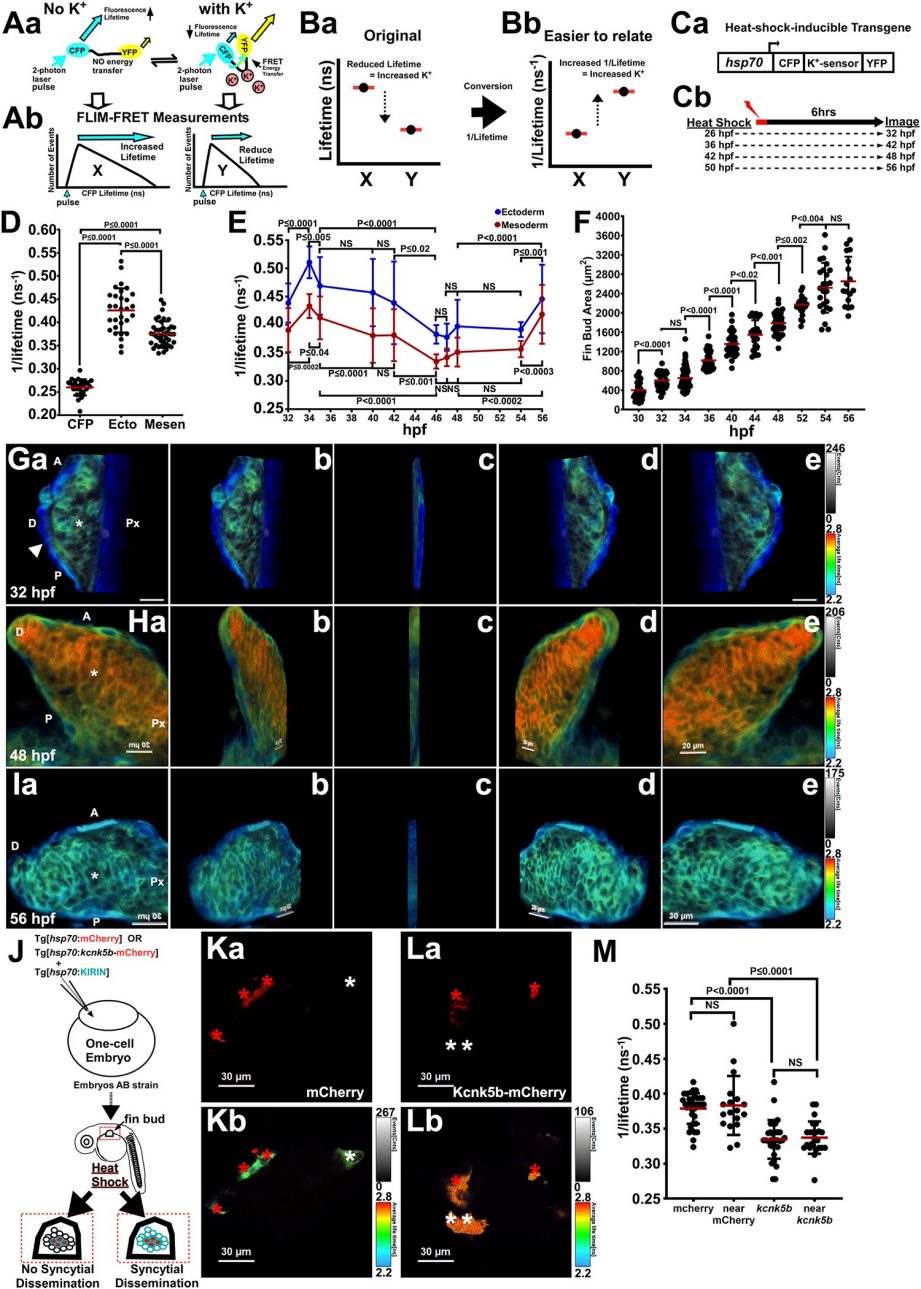
Intracellular K^+^ decreases during pectoral fin bud growth. **(A)** FRET-based mechanism of detection of intracellular K^+^ by KIRIN1 sensor and application of FLIM assessment. Changes in FRET affect the fluorescence lifetime of the donor fluorophore CFP that is excited by a 2-Photon laser pulse (**a**). A reduction of intracellular K^+^ is detected as higher (longer) lifetime of the CFP, while increase in K^+^ is detected as its lower (shorter) lifetime (**b**). **(B)** We depict these lifetime (nanoseconds, ns) relationships of higher lifetime equating to lower intracellular K^+^ (**a**) and the inverse (original) as 1/lifetime (ns^-1^) (**b**) in order to more easily relate the trend changes with what the FLIM-based measurements are indicating for intracellular K^+^. **(C)** Design of K^+^ sensor (KIRIN1) transgene for transgenic zebrafish line (**a**). Heat-shock method for inducing transgene during different developmental time points of the fin bud and subsequently FLIM-FRET (FILM) imaging it (**b**). **(D)** FLIM measurements of the CFP in confocal sections of ectoderm (Ecto) and mesenchyme (Mesen) in pectoral fin buds at 48 hpf using a FRET-based sensor for K^+^. Measurements from a transgene containing only CFP that is unable to FRET with YFP. **(E)** Time course of FLIM measurements of intracellular K^+^ in the ectodermal and mesenchymal tissues of the developing pectoral fin buds. **(F**) Time course of growth measurements of the developing fin buds. **(G–I)** Z-stacks of confocal FLIM planes in fin buds at 32 hpf (G), 48 hpf (H), and 56 hpf (I). Z-stacks cover distances of 15.2 μm (32 hpf), 18.36 μm (48 hpf), and 10.3 μm (56 hpf). The lifetime value of each pixel in the ectoderm (white arrowhead) and the mesenchyme (asterisk) is represented by colors in a rainbow scale from 2.8 ns (blue) to 3.4 ns (red). **(J)** Generation of fin buds that mosaically express the KIRIN1 sensor and either *mCherry* or *kcnk5b*-mCherry (mosaic for 2 transgenes) and proposed outcomes. (**Ka,b**) Images of cells in fin buds of 56 hpf embryos harboring *hsp70*:mCherry (red asterisks) and cells lacking the mCherry transgene (white asterisks). Confocal plane for mCherry fluorescence (**a**). FLIM image of KIRIN1+ cells of the same confocal plane (**b**). (**La,b)** Images of cells in fin buds of 56 hpf embryos harboring *hsp70*:*kcnk5b*-mCherry (red asterisks) and cells lacking the transgene (white asterisks). Confocal plane for mCherry fluorescence (**a**). FLIM image of KIRIN1+ cells of the same confocal plane (**b**). **(M)** FLIM measurements in fin buds of 56 hpf embryos of indicated cell categories; “near” indicates cells next or distant to mCherry-positive (mCherry+) or *kcnk5b*-mCherry-positive (*kcnk5b*+) cells. The graphs are depicted as 1/lifetime (ns^-1^) to more easily relate the portrayal of the values to the related change in intracellular K^+^ (see S1A Fig). Experiments were repeated 3 or more time (*N* ≥ 3). For FLIM, we measured 2 or 3 locations in each tissue of 1 fin bud per embryo. Each measured value is represented as a data point (D, M), except for E, in which the data points are presented as averages and standard deviations of all the measurements (≥11) at each time point. For fin bud size measurements (F), each data point represents 1 fin bud per embryo. *P* values represent statistical analysis by Student’s two-tailed *t* test. *P* values >0.05 are designated as “not significant” (NS). Numerical data used in this figure are included in S1 Data. FLIM, Fluorescence Lifetime Microscopy; FRET, Förster Resonance Energy Transfer; hpf, hours post fertilization.

To measure intracellular K^+^, we used an established FRET-based genetic sensor (KIRIN1) that can detect changes specifically in intracellular K^+^ levels [28]. To overcome the limitations associated with FRET measurements in vivo, we used FLIM, because FLIM does not rely on intensity-based ratios, rather, FLIM detects changes in the intrinsic decay rate of a donor fluorophore (CPF) when it undergoes FRET to the acceptor fluorophore (YFP) within the sensor (**Fig 1Aa and 1Ab**). One can directly quantify the accumulated decay profile of a fluorophore (CFP) after excitation by laser pulses [29] (**Fig 1Ba and 1Bb**); consequently, we could detect relative differences in intracellular K^+^ caused by increases in K^+^-leak channel expression (**S1Aa and S1Ab Fig**), or by posttranslational regulation of a K^+^-leak channel (**S1B Fig**) or by different K^+^-leak channel mutants that have different activities **(S1C Fig)** [11]. These experiments confirmed that we can detect changes in relative intracellular K^+^ levels with the KIRIN1 sensor using FLIM.

To assess the intracellular K^+^ in vivo, we generated a heat-shock-inducible transgenic reporter Tg[*hsp70*:CFP-KIRIN1-YFP] (*hsp70*:KIRIN1) line (**Fig 1Ca**) and used it to detect relative differences in K^+^ levels via FLIM 6 h after heat shock (**Fig 1Cb**). FLIM measurements in the pectoral fin bud showed relatively lower intracellular K^+^ levels in the mesenchyme compared to ectoderm at 48 hpf (**Fig 1D**), a time point in which the growth of the pectoral fin buds is underway. We subsequently measured several time points during pectoral fin bud outgrowth from the same animals, and we observed an increase in intracellular K^+^ in both the ectoderm and the mesenchyme between 32 and 34 hpf, but afterwards, intracellular K^+^ levels decreased to their lowest levels at around 48 hpf (**Fig 1E**). Intracellular K^+^ levels increased again between 54 and 56 hpf (**Fig 1E**). From the plotted time points, we observed that although mesenchyme K^+^ levels remained lower than the ectoderm, the changes in K^+^ in both tissues were coupled (**Fig 1E**). These lifetime measurements were not influenced by differences in intensity levels (**S2D–S2H Fig**) or by the method for immobilizing the embryos (**S2I–S2O Fig**). To ascertain whether there is a relationship between relative changes in intracellular K^+^ and growth, we measured fin bud sizes between 30 hpf to 56 hpf. From bud area measurements, we observed incremental growth from 30 hpf to 56 hpf (**Fig 1F**). We also observed 2 pauses in average growth between 32 hpf and 34 hpf and 54 hpf and 56 hpf (**Fig 1F**) that correlated with time points in which intracellular K^+^ increased (**Fig 1E**). Together, these results suggested a coordinated regulation of intracellular K^+^ that relates to fin bud growth.

To visualize the relative distribution of intracellular K^+^ during pectoral fin bud development, we represented the lifetime values along a rainbow scale: the lower the value (the higher intracellular K^+^ levels), the more towards blue; conversely, the higher values (the lower intracellular K^+^ levels), the more towards red. From the colorized individual confocal FLIM planes (**S3A–S3C Fig**) and 3D Z-stacks of FLIM planes through fin buds at 32 hpf (**Figs 1Ga–1Ge and S3A**), at 48 hpf (**Figs 1Ha–1He and S3B**), and at 56 hpf (**Figs 1Ia–1Ie and S3C**), we observed clear differences in K^+^ levels between the mesenchyme and ectoderm, but few regional differences within each tissue. We also observed global decreases in intracellular K^+^ between 32 hpf (**Figs 1Ga–1Ge and S3A**) and 48 hpf (**Figs 1Ha–1He and S3B**) when the growth of the bud is high. Conversely, we observed global increases in K^+^ between 48 hpf and 56 hpf (**Figs 1Ia–1Ie and S3C**) when growth reduces. From these observations, we propose that there is a decrease in intracellular K^+^ during fin bud growth due to increases in K^+^-leak channel activity, and these changes are linked to the growth of the bud.

The lack of regional differences in intracellular K^+^ in either the mesenchyme or ectoderm suggested equilibration of K^+^ between the cells of each tissue. To test this hypothesis, we generated double mosaic embryos by injecting recipient embryos with the heat-shock-inducible *hsp70*:KIRIN1 and either *kcnk5b*-mCherry transgene (*hsp70*:*kcnk5b*-mCherry) or with mCherry (*hsp70*:mCherry) transgene as a control (**Fig 1J**). We identified mCherry-expressing cells (**Fig 1K**) or *kcnk5b*-mCherry cells (**Fig 1L**) in fin buds and then assessed intracellular K^+^ in mCherry-positive cells (**Fig 1Ka, 1La, and 1M** and red asterisks in **Fig 1Kb and 1Lb**) and the surrounding mCherry-negative cells (**Fig 1K and 1L**, white asterisks). Compared to cells that harbored the mCherry transgene (**Fig 1K**, red asterisks), we observed that cells expressing *kcnk5b*-mCherry (**Fig 1L**, red asterisks) and cells surrounding the transgene-expressing cells displayed decreases in K^+^ levels (**Figs 1M and S3D**). These data indicated that changes in intracellular K^+^ are shared such that cells with more K^+^-leak channel activity can decrease the intracellular K^+^ of neighboring cells.

### Overexpression of a K^+^-leak channel in early fin bud coordinately enhances the expression of the important morphogens to scale proximodistal growth

**Fig 2:**
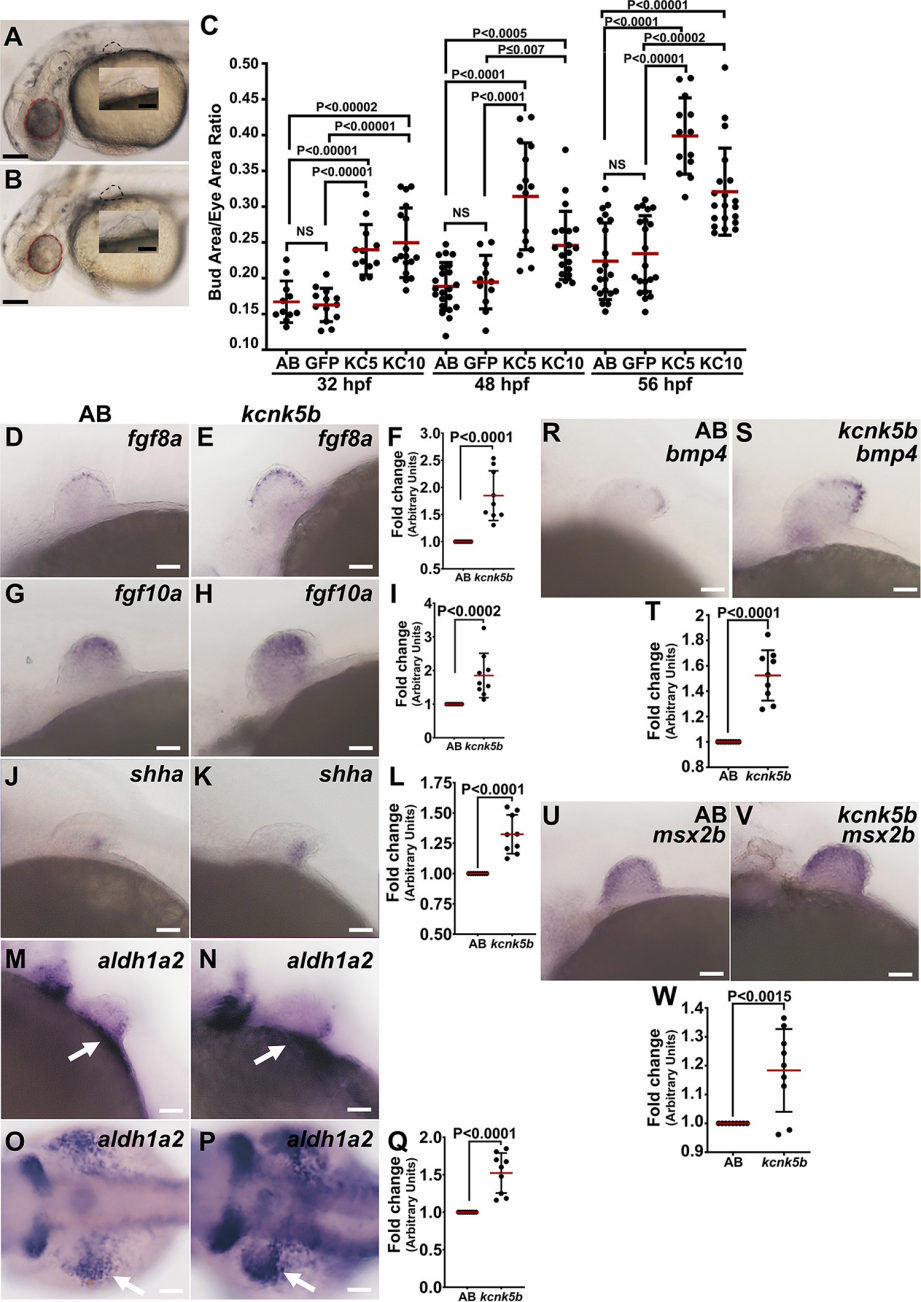
K^+^-leak channels enhance the growth of pectoral fin buds. **(A, B)** Brightfield images of a post-heat-shocked 48 hpf non-transgenic embryo (A) or post-heat-shocked 48 hpf *hsp70*:*kcnk5b*-GFP transgenic embryo (B). The area of the fin bud (black-dotted line). **(C)** Measurements of pectoral fin bud areas of heat-shocked groups of non-transgenic (AB), Tg[*hsp70*:GFP], Tg[*hsp70*:*kcnk5b*-GFP], and Tg[*hsp70*:*kcnk10*-GFP]. The non-transgenic and Tg[*hsp70*:*kcnk5b*-GFP] embryos are siblings. Each measured bud was standardized to the eye area (red-dotted circles in A,B) in the same embryo. **(D–S)** Expression of the indicated morphogens. **(D, G, J, M)** Lateral views of whole-mount in situs of heat-shock control non-transgenic embryos at 48 hpf for *fgf8a* (**D)**, *fgf10a* (**G),** *shha* (at 52 hpf) (**J**), *aldh1a2* (**M**) and dorsal view of *aldh1a2* (**O**) or lateral views of heat-shocked Tg[*hsp70*:*kcnk5b*-GFP] embryos at 48 hpf for *fgf8a* (**E)**, *fgf10a* (**H)**, *shha* (at 52 hpf) **(K)**, *aldh1a2* (**N**), and dorsal view of *aldh1a2* (**P**). White arrows indicate proximal expression of *aldh1a2*. qRT-PCR measurements from fin buds for *fgf8a* (**F**), *fgf10a* (**I**), *shha* (**L**), and *aldh1a2* (**Q**). Lateral views of in situs of heat-shocked non-transgenic fin bud for *bmp4* (**R**) and *msx2b* (**U**) and of heat-shocked Tg[*hsp70*:*kcnk5b*-GFP] embryos at 48 hpf for *bmp4* (**S**) and *msx2b* (**V**). qRT-PCR measurements from fin buds for *bmp4* (**T**), *msx2b* (**W**) the in situ experiments were repeated 3 or more time (*N* ≥ 3). Each in situ repeat contains 6–12 embryos per replicate. For the fin bud size measurements, we measured 1 fin bud and eye per embryo. We measured at least 4 embryos per experimental repeat. For the qRT-PCRs, we collected 80 fin bud samples per isolation. We assessed gene expression with 3 or more isolations. Each isolation was assessed in duplicate or triplicate. Each measured value is represented as a data point. *P* values represent statistical analysis by Student’s two-tailed *t* test. *P* values ≥0.05 are designated as “not significant” (NS). The scale bars are 0.5 mm (A, B), 100 μm (D–P, R–S,U–T). Numerical data used in this figure are included in S2 Data. hpf, hours post fertilization.

The coordinated decrease in the endogenous levels of K^+^ in the developing pectoral fin bud suggested that intracellular K^+^ has a role in bud development. Consequently, we wished to know whether decreasing intracellular K^+^ is sufficient to enhance the scale of the buds. To decrease intracellular K^+^, we overexpressed Kcnk5b or Kcnk10 K^+^-leak channel by heat-shock induction of the Tg[*hsp70*:*kcnk5b*-GFP] [11] or Tg[*hsp70*:*kcnk10a*-GFP] transgenic lines. Six hours after transgene induction at 48 hpf (**S4A–S4C Fig**), we measured the growth area of the fin buds and standardized the measurements of each bud to the area of the eye (**Fig 2A and 2B**) or the otic vesicle (**S4D and S4E Fig**). From these analyses, we observed enhanced growth of pectoral fin buds caused by *kcnk5b* or *kcnk10* compared to heat-shocked non-transgenic siblings and transgenic Tg[*hsp70*:*GFP*] control groups at 32 hpf, 48 hpf, and 56 hpf when standardized to the area of the eye (**Fig 2C)** or of the area of the otic vesicle (**S4F Fig**). Together, these data indicated that reducing intracellular K^+^ increases growth of the buds.

The increase in pectoral fin bud size from K^+^-leak channel overexpression indicated that the developmental gene program was affected. We therefore examined the expression of selected morphogens known to control early bud growth at 48 hpf. We observed that compared to heat-shocked control siblings (**Fig 2D, 2G, 2J, 2M and 2O**), transgenic expression of *kcnk5b* increased the expression of *fgf8a* (**Fig 2D and 2E**), *fgf10a* (**Fig 2G and 2H**), *shha* (**Fig 2J and 2K**), and *aldh1a2* (**Fig 2M–2P**). We observed similar up-regulation of these genes from qRT-PCR analyses of fin buds (**Fig 2F, 2I, 2L and 2Q**). Because previous findings show that BMP signaling in vertebrate and invertebrate appendages is affected by K^+^ channels [5,6], we also assessed the expression of an important BMP ligand (*bmp4*) and its down-stream target *msx2b*. We observed that compared to in situ controls (**Fig 2R and 2U**), *kcnk5b* increased the expression of both genes in the in buds (**Fig 2S and 2V**). We also observed similar up-regulation of these genes by qRT-PCR (**Fig 2T and 2W**). Thus, the endogenous decreases in intracellular K^+^ during fin bud development (**Fig 1**), the coordinated increase in the expression of several important morphogens by increasing the expression of the K^+^-leak channel *kcnk5b* (**Fig 2D–2W**), and the enhanced growth the pectoral fin bud by *kcnk5b* or *kcnk10a* overexpression (**Fig 2C**) all implicated intracellular K^+^ as an integral part of the fin bud growth control, specifically, decreased intracellular K^+^ augmented bud proportional growth.

### Retinoic acid decreases intracellular K^+^ via an Rcan2-mediated mechanism that scales fin buds

**Fig 3:**
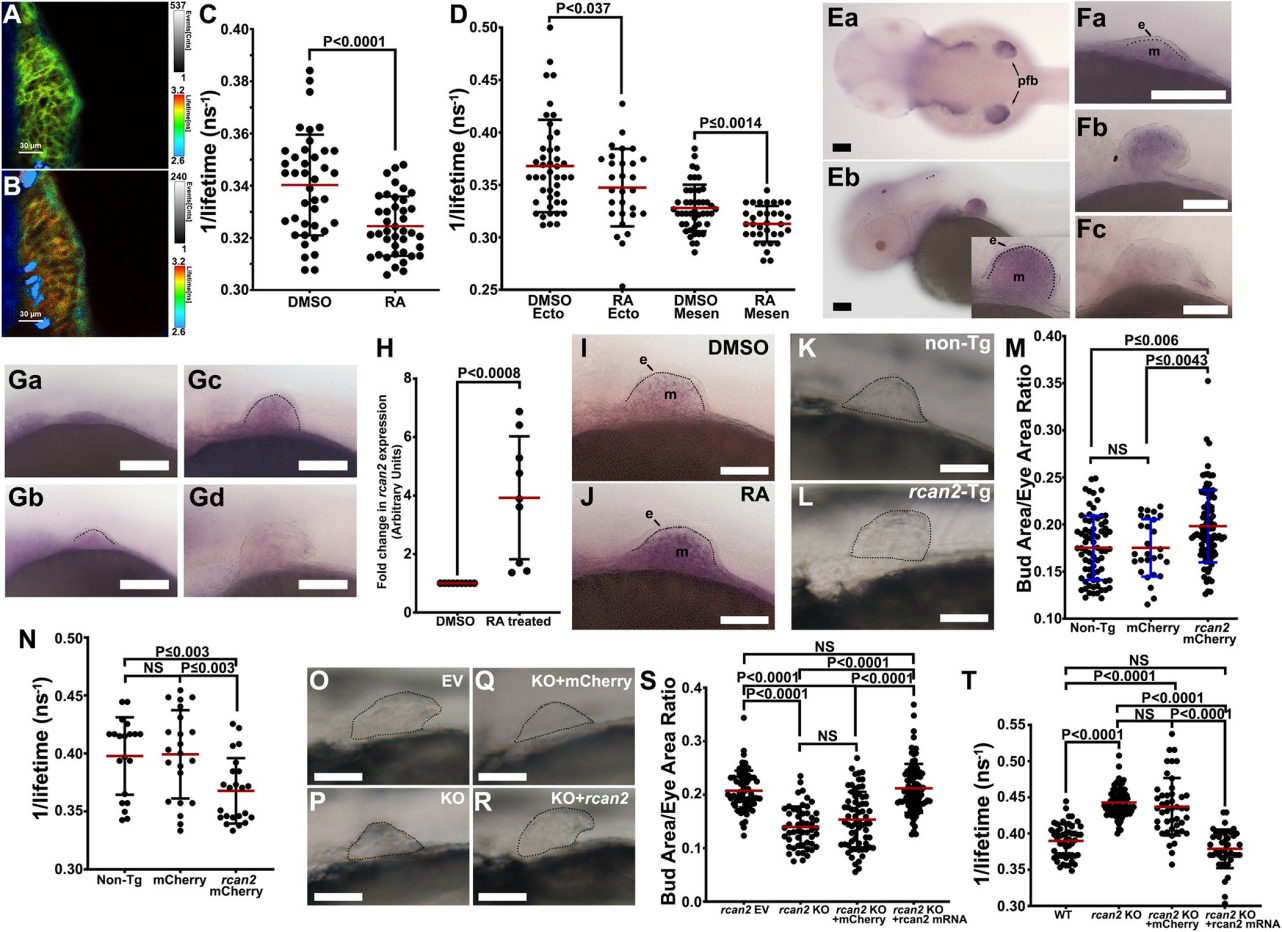
Retinoic acid decreases intracellular K^+^ in pectoral fin buds via transcriptional activation of the calcineurin inhibitor *rcan2*. **(A, B)** FLIM images of developing fin buds from embryos treated for 6 h with DSMO-treated (A) or with 200 nM RA-treated (B) 32 hpf embryos. **(C)** FLIM measurements from cells in developing buds at 32 hpf of the indicated treatment groups. **(D)** FLIM measurements of cells in the ectoderm (Ecto) or the mesenchyme (Mesen) of buds at 32 hpf treated either with DMSO or RA. **(E)** In situ images of dorsal view (**a**) and lateral view (**b**) for *kcnk5b* expression in 48 hpf embryos. Pectoral fin buds (pfb), ectoderm (e), and mesenchyme (m). **(F)** Representative lateral view images of pectoral fin buds of in situ experiments for *kcnk5b* expression at 32 hpf (**a**), 56 hpf (**b**), and 72 hpf (**c**). **(G)** Representative lateral views of pectoral fin buds after in situ experiments for *rcan2* expression at 32 hpf (**a**), 34 hpf (**b**), 48 hpf (**c**), and 56 hpf (**d**) embryos. **(H)** qRT-PCR of *rcan2* from isolated pectoral fin buds of 48–52 hpf embryos treated with DMSO or 200 nM RA for 6 h. **(I)** In situ for *rcan2* expression in fin buds of 48 hpf embryos after 6 h DMSO treatment. Ectoderm is indicated by (e), and mesenchyme is indicated by (m). **(J)** In situ images for *rcan2* expression in fin buds of 48 hpf embryo after 6 h 200 nM RA treatment. Ectoderm (e), mesenchyme (m). **(K, L)** Pectoral fin buds of heat-shocked non-transgenic embryo (K) or heat-shocked transgenic *hsp70*:*rcan2*-mCherry sibling (L). **(M)** Measured fin buds were standardized to the area of an eye in the same embryo. **(N)** FLIM measurements of intracellular K^+^ in pectoral fin bud cells from heat-shocked non-transgenic and mCherry controls and transgenic *rcan2*-mCherry-expressing siblings. **(O–R)** Representative fin buds of embryos expressing empty sgRNA vector “EV” (O), *rcan2* sgRNA “KO” (P), mCherry mRNA with *rcan2* sgRNA “KO+mCherry” (Q), mutated rescue *rcan2* mRNA with *rcan2* sgRNA “KO+*rcan2*” (R). **(S)** Ratios of fin bud areas to eye areas of embryos of the indicated experimental groups. **(T)** FLIM measurements of intracellular K^+^ in the pectoral fin bud cells in control and CRISPR-Cas9 knockout of *rcan2* in embryos. Experiments were repeated 3 or more time (*N* ≥ 3). For FLIM, we measured 2 or 3 locations in each tissue of 1 fin bud per embryo. Each measured value is represented as a data point (C, D, N, T). For the fin bud size measurements, we measured 1 fin bud per embryo (M) and 2 fin buds per embryo (S). For the qRT-PCR experiments, we collected 80 fin bud samples per isolation. Three or more isolations were measured. Each isolation was measured in duplicate or triplicate. Each isolation is represented as a data point. Each in situ repeat contained 6–12 embryos per replicate. *P* values represent statistical analysis by Student’s two-tailed *t* test. *P* values >0.05 are designated as “not significant” (NS). Scale bars represent 50 μm (E–G, I–L, O–R). Numerical data used in this figure are included in S3 Data. FLIM, Fluorescence Lifetime Microscopy; hpf, hours post fertilization; RA, retinoic acid.

The decrease in intracellular K^+^ during pectoral fin bud growth suggested that K^+^ levels might be responsive to developmental signals that regulate growth. One such signal is RA [30–32]. Therefore, we examined whether RA stimulation influenced intracellular K^+^ levels in developing fin buds at 32 hpf by FLIM measurements of the KIRIN1 transgene 6 h after its heat-shock induction. Compared to treatment with the solvent DMSO (**Fig 3A and 3C**), we observed decreases in K^+^ after 6 h of treatment with 200 nM RA (**Fig 3B and 3C**), both in ectoderm and mesenchyme cells (**Fig 3D**). To test whether the changes in intracellular K^+^ were specific to RA, we assessed the effect of thyroid hormone (TH), another nuclear hormone receptor mechanism. Treatment with TH showed no significant differences in K^+^ levels compared to DMSO-treated controls (**S5C Fig**). These data indicated that RA-mediated signaling is sufficient to decrease intracellular K^+^ in fin bud tissues and that this effect is not induced by all nuclear hormones.

RA regulates gene transcription via specific intracellular receptors [33]. Consequently, the relative reduction in K^+^ by RA suggests that RA regulates the transcription of one or more K^+^ channels. Because Kcnk5b is involved in adult fin scaling [9], we performed in situ hybridization experiments to determine whether *kcnk5b* is present in growing fin buds of 48 hpf embryos. We observed expression primarily in distal pharyngeal pouches (**Fig 3Ea**), and the mesenchyme of the growing fin buds (**Fig 3Ea and 3Eb**). We subsequently assessed *kcnk5b* expression during fin bud growth at 34 hpf, at 56 hpf, and at 72hpf, a time point in which bud growth has already ceased. We observed *kcnk5b* expression primarily in the mesenchyme at 32 hpf (**Fig 3Fa**) and at 56 hpf (**Fig 3Fb**), but by 72 hpf, the channel expression was difficult to detect in the bud (**Fig 3Fc**). Because treatment with RA decreases intracellular K^+^ levels, we reckoned that RA may be enhancing *kcnk5b* transcription. Therefore, we tested whether RA-mediated decrease in intracellular K^+^ was related to an up-regulation of *kcnk5b* expression. We did not observe an increase in RA-treated embryos by qRT-PCR (**S5A Fig**) despite a significant increase in a known RA-activated gene (**S5B Fig**) [34].

We previously showed that inhibition of calcineurin increases the activity of Kcnk5b to decrease intracellular K^+^ and enhance the scale of the adult fin [11]. Since Kcnk5b is present in the embryonic pectoral fin bud (**Fig 3E and 3F**), we hypothesized that RA may decrease intracellular K^+^ by posttranslational regulation of Kcnk5b through the inhibition of calcineurin. RCAN proteins are well-documented in vivo inhibitors of calcineurin [35–37]. From in situ hybridization experiments, we detected expression at 32 hpf (**Fig 3Ga**), at 34 hpf (**Fig 3Gb**), and 48 hpf (**Fig 3Gc**). However, *rcan2* expression reduced by 56 hpf (**Fig 3Gd**). Because RA treatment decreased intracellular K^+^ and Rcan2 inhibits calcineurin, which subsequently could suppress calcineurin-mediated inhibition of Kcnk5b, we tested whether *rcan2* expression is altered by RA. We observed that compared to DMSO controls, RA treatment increased *rcan2* expression by qRT-PCR (**Fig 3H**), and from in situ hybridization experiments, we observed staining in DMSO-treated buds (**Fig 3I**) that became more intense after RA treatment (**Figs 3J and S5D**). We also observed similar phenomena in the adult zebrafish fins: RA treatment decreased intracellular K^+^ levels (**S5G Fig**) and increased *rcan2* transcription (**S5H Fig**). Furthermore, we detected *rcan2* transcripts and protein in the blastemas and distal regenerating interray tissue of regenerating fins (**S5I–S5K Fig**), where growth occurs. We did not observe protein expression in fin rays immediately after amputation when regenerative growth has not yet commenced (**S5J and S5K Fig**).

The ability of RA to up-regulate *rcan2*, which inhibits a phosphatase that suppresses the K^+^-leak channel Kcnk5b (present and scales fin buds and adult fins [9,11]) led us to hypothesize that Rcan2 may enhance proportional growth of fin buds. To test whether increasing Rcan2 enhances pectoral fin bud growth, we generated a fish line harboring the *hsp70*:*rcan2*-mCherry transgene to overexpress *rcan2* by heat-shock induction (**S5L–S5O Fig**). Compared to heat-shocked non-transgenic control siblings (**Fig 3K and 3M**) and overexpressed mCherry (**Fig 3M**), induction of *rcan2* enhanced the growth area of the fin buds (**Fig 3L and 3M**). We also observed enhanced growth by transgenic overexpression of *rcan2* during adult fin regeneration (**S5P–S5V Fig**). To determine whether *rcan2*-enhanced growth correlated with reduced intracellular K^+^, we generated double-transgenic fish that harbored *hsp70*:*rcan2*-mCherry and *hsp70*:KIRIN1 transgenes. After heat-shock induction of the transgenes, we observed that *rcan2* expression decreased intracellular K^+^ levels compared to controls (**Fig 3N**). Together, these data showed that induction of *rcan2* expression was sufficient to enhance growth in the fin bud and adult fin as well as reduce their intracellular K^+^ levels in vivo.

We then assessed whether targeting *rcan2* by CRISPR-Cas9 (**S5W–S5Z Fig**) affected fin bud size and intracellular K^+^. Compared to control embryos (**Fig 3O and 3S**), we observed that *rcan2*-targeted embryos displayed reduced fin bud growth (**Fig 3P, 3Q and 3S**), which could be rescued by overexpression of *rcan2* mRNA (**Fig 3R and 3S**) that harbored mutations in its wobble bases to impair interactions between the overexpressed mRNA and the genome-targeting sgRNA (**S5Za and S5Zb Fig**). We also assessed caudal fins of CRISPR-Cas9-targeted juvenile fish, which also displayed shorter fins compared to wild type (**S5AA and S5BB Fig**). When we assessed the effect of targeting *rcan2* on intracellular K^+^ levels by targeting *rcan2* in Tg[*hsp70*:KIRIN1] embryos, we observed that targeting *rcan2* increased intracellular K^+^ (**Fig 3T**) and rescue with *rcan2* mRNA returned intracellular K^+^ back to control levels (**Fig 3T**). Together, these results from embryonic fin buds and post-metamorphosis caudal fins indicated the involvement of *rcan2* in K^+^-channel-mediated scaling of embryonic and adult appendages.

### Rcan2-mediated scaling requires Kcnk5b activity via its Serine 345

**Fig 4:**
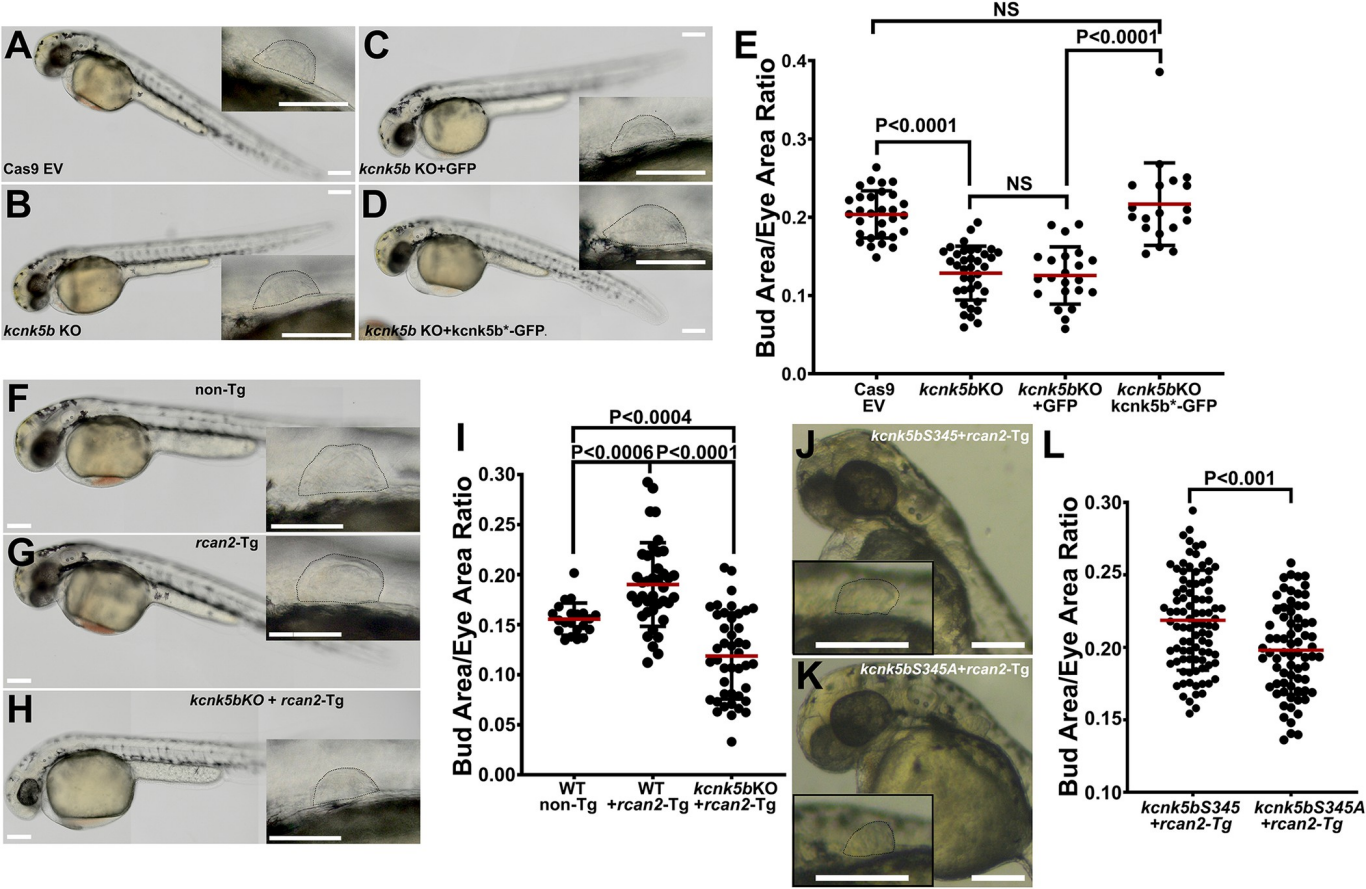
Rcan2-mediated decrease in intracellular K^+^ involves Kcnk5b. **(A–D)** Representative 48 hpf embryos and enlarged panels of fin buds of control Cas9 and empty sgRNA vector (EV) embryo (A), CRISPR-targeted *kcnk5b* embryo (B), CRISPR-targeted *kcnk5b* embryo overexpressing GFP (C), CRISPR-targeted *kcnk5b* embryo overexpressing kcnk5b*-GFP that harbors altered wobble bases to impair interaction with targeting sgRNA (D). **(E)** Fin bud-to-eye area ratios of the indicated genotypes. **(F–I)** Representative 48 hpf embryos and enlarged panels of fin buds of control Cas9 and empty sgRNA vector (EV) non-Tg embryo (F), *rcan2*-mCherry-expressing embryo and enlarged panel of the fin bud (G) CRISPR-targeted *kcnk5b* embryo overexpressing *rcan2*-mCherry (H). **(I)** Fin bud-to-eye area ratios of the indicated genotypes. **(J, K)** Representative 48 hpf embryos and enlarged panel of the fin buds expressing wild-type *kcnk5bS345*-GFP and *rcan2*-mCherry (J), or expressing calcineurin-dephosphorylated mimic *kcnk5bS345A*-GFP and *rcan2*-mCherry (K). **(L)** Fin bud-to-eye ratios show that *rcan2*-mediated enlargement of fin buds is impaired by *kcnk5bS345A* mutant. Experiments were repeated 3 or more time (*N* ≥ 3). For the fin bud size measurements, we measured 1 fin bud (E, I) or 2 fin buds (L) per embryo at 48 hpf and not at a later time points to avoid incorporating measurements of the finfold growth that start around 56 hpf. Each measured value is represented as a data point. *P* values represent statistical analysis by Student’s two-tailed *t* test. *P* values >0.05 are designated as “not significant” (NS). Scale bars equal 100 μm (**A–D, F–H, J, K**). Numerical data used in this figure are included in S4 Data. hpf, hours post fertilization.

Calcineurin limits fin proportional growth by inhibiting Kcnk5b through serine 345 [11]. Since Rcan2 inhibits calcineurin [35–37], and *kcnk5b* is expressed in the developing pectoral fin buds (**Fig 3E and 3F**), we first assessed the importance of *kcnk5b* in fin bud growth by CRISPR-targeting *kcnk5b* (**S6A–S6G Fig**). Compared to controls (**Fig 4A** and **4E**), fin buds of *kcnk5b*-targeted embryos were reduced in size by 48 hpf (**Fig 4B and 4E**). This phenotype was rescued by overexpression of *kcnk5b* mRNA (**Fig 4D and 4E**) that was mutated to impair interaction with the targeting sgRNA (**S6H–S6J Fig**). When we overexpressed *rcan2* in *kcnk5b*-targeted embryos, we observed that while *rcan2* overexpression in non-targeted embryos enhanced fin bud growth (**Fig 4F, 4G and 4I**), *rcan2* required *kcnk5b* to enhance growth (**Fig 4H and 4I**). We previously showed that calcineurin inhibits Kcnk5b via serine 345 in the cytoplasmic tail of the channel [11], so we tested whether the *kcnk5bS345A* (the mutant with the lowest K^+^-leak activity) blunts *rcan2* enhancement of fin bud growth in vivo. Compared to control embryos overexpressing *rcan2* with the wild-type channel (*kcnk5bS345*) (**Fig 4J and 4L**), the calcineurin-dephosphorylated mimic *kcnk5bS345A* channel reduced *rcan2*-mediated fin bud growth (**Fig 4K and 4L**). Together, these data indicated that *rcan2*-enhanced growth requires Kcnk5b and that the Kcnk5b channel that mimics calcineurin-mediated inhibition of the channel prevents the enhanced growth caused by overexpression of the endogenous calcineurin inhibitor *rcan2*.

### Kcnk5b-enhanced growth involves cell depolarization

**Fig 5:**
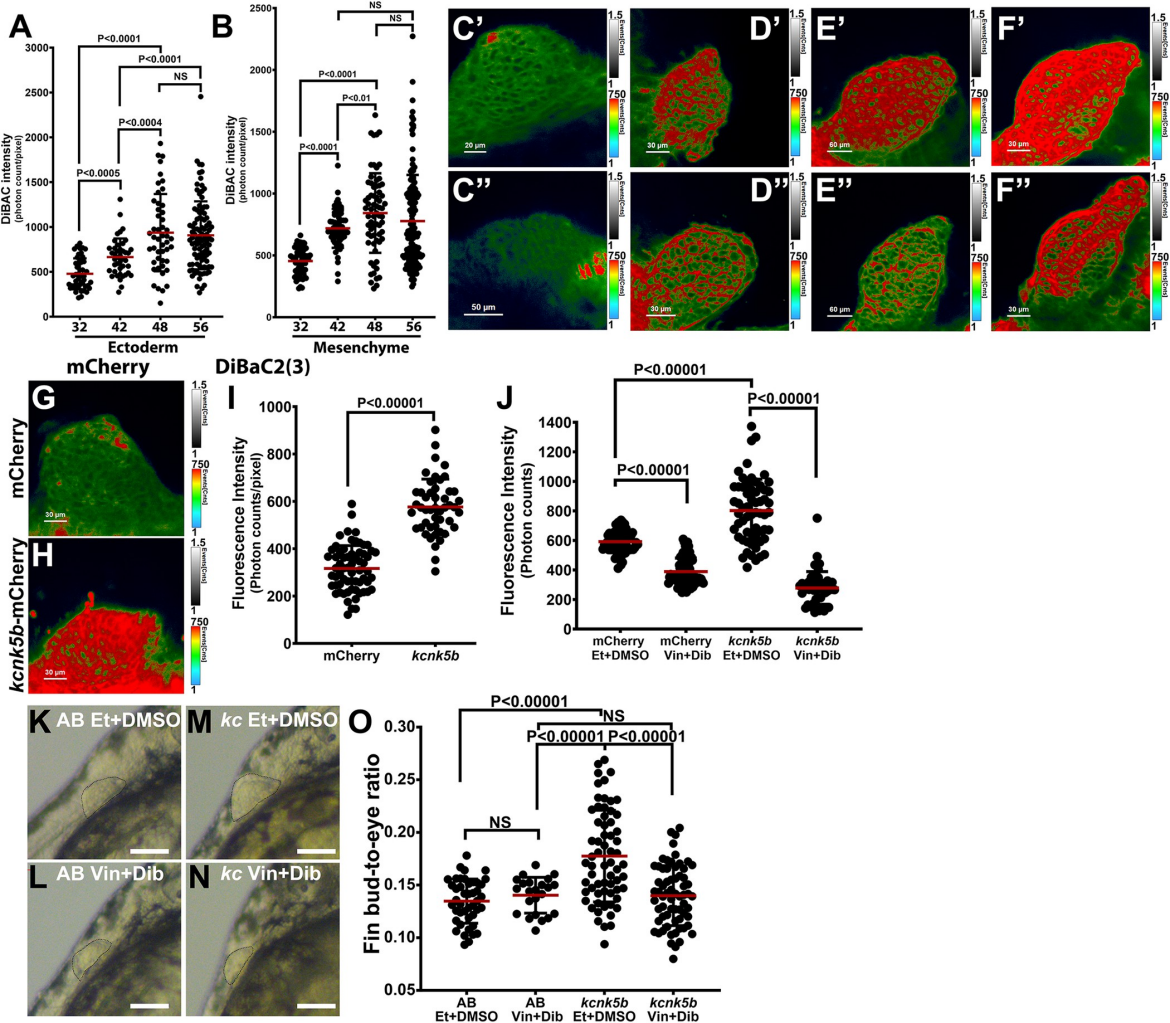
Kcnk5b enhanced depolarization is required for enhanced fin bud growth. **(A, B)** DiSBAC_2_(3) fluorescence measurements of the ectoderm (A) and mesenchyme cells (**B**) of developing pectoral fin buds at 32, 42, 48, and 56 hpf. **(C–F)** Confocal images of developing fin buds displaying the intensities fluorescence as photon counts per pixel at 32 hpf (**C**), 42 hpf (**D**), 48 hpf (**E**), and 56 hpf (**F**). Colors represent the range of counted photons per pixel. Blue representing the lowest level of counted photons, and red representing their highest counts (up to 750 photons or more). Images representing high photon count (**C’, D’, E’, F’**) and low photon count (**C”, D”, E”, F”**). The total exposure range was set at 1,500 counts (1.5). **(G)** Distribution of counted photons from DiSBAC_2_(3) in the confocal plane of the representative fin bud at 32 hpf. **(H)** Distribution of counted photons from DiSBAC_2_(3) in the confocal plane of the representative fin bud at 32 hpf. **(I)** Assessment of DiSBAC_2_(3) fluorescence intensity as counted photons for fin buds expressing mCherry or *kcnk5b*-mCherry**. (J)** Assessment of DiSBAC_2_(3) fluorescence intensity of mCherry-expressing or *kcnk5b*-mCherry fin buds at 32 hpf after treating for 4 h as indicated: ethanol (Et), DMSO, 10 μm vinpocetine (vin), 40 μm dibucaine (dib). **(K)** Representative image of pectoral fin bud of non-transgenic 48 hpf AB fish after heat shock at 32 hpf and start of treatment at 36 hpf with the drug solvents Ethanol (Et) and DMSO. **(L)** Representative image of pectoral fin buds of 48 hpf AB fish heat shocked at 32 hpf and start of treatment with 10 μm Vinpocetine (Vin) and 40 μm Dibucane (Dib) at 36 hpf. **(M)** Representative image of pectoral fin buds of 48 hpf transgenic Tg[*hsp70*:*kcnk5b*-GFP] after heat shock at 32 hpf and start of treatment with EtOH and DMSO at 36 hpf. **(N)** Representative image of pectoral fin buds of 48 hpf transgenic Tg[*hsp70*:*kcnk5b*-GFP] after heat shock at 32 hpf and start of treatment with 10 μm Vin and 40 μm Dib at 36 hpf. **(O)** Assessment of pectoral fin bud growth at 48 hpf expressing either AB and *kcnk5b*-GFP in the indicated treatment groups. Experiments were repeated 3 or more time (*N* ≥ 3). Each repeat contained 6 or more embryos; one fin bud was measured per embryo. For the DiSBAC_2_(3) fluorescence measurements, 6 independent points were measured from different 4 locations in each fin bud, distal, anterior, posterior, and proximal, and then averaged to represent a data point (A, B, I, J). For the fin bud size measurements, we measured 1 fin bud and eye per embryo. We measured at least 15 embryos per repeat, and each measurement is 1 data point (O). *P* values represent statistical analysis by Student’s two-tailed *t* test. *P* values >0.05 are designated as “not significant” (NS). Scale bars equal 100 μm. Numerical data used in this figure are included in S5 Data. hpf, hours post fertilization.

Kcnk5b is a two-pore K^+^ leak channel whose activity alters the electrical membrane potential at the plasma membrane of cells. The decrease in intracellular K^+^ during fin bud growth (**Fig 1**) and the importance of Kcnk5b for growth (**Fig 4**) suggest there are important K^+^-associated changes in membrane potential during fin bud development. To determine whether membrane potential alters during fin bud development, we used DiSBAC_2_(3), a dye that increases its fluorescence as it enters cells when channels open to depolarize the cells [38]. We assessed DiSBAC_2_(3) fluorescence using time-correlated single photon counting photodetectors (same used for FLIM) that count the emitted photons per pixel in a confocal plane. From measurements at different locations in confocal planes of several fin buds, we observed the least amount of depolarization at 32 hpf in the ectoderm (**Fig 5A**) and the mesenchyme (**Fig 5B**). Afterward, depolarization increased by 42 hpf with average highest levels at 48 hpf and 56 hpf (**Fig 5A and 5B**). While the averages increased, these averages represented a broad distribution of depolarization levels particularly at 48 hpf and 56 hpf. To visualize the distribution of relative differences in membrane potential at the selected time points, we depicted the photon-count information of each pixel in mid-level confocal planes of the fin buds using a rainbow color scale in which red represented the highest levels of DiSBAC_2_(3) fluorescence (depolarization) and green to blue depicted lower levels. We observed that 32 hpf consistently showed the lowest levels of depolarization (**Fig 5C’ and 5C”**). DiSBAC_2_(3) fluorescence incrementally increased throughout the fin buds at 42 hpf (**Fig 5D’**), 48 hpf (**Fig 5E’**), and 56 hpf (**Fig 5F’**). As indicated by the variance in our measurements, we also observed fin buds at these time points that displayed lower levels of fluorescence or variegated patterns high and low fluorescence (**Fig 5D”, 5E” and 5F”**). We posit that the observed variation in relative membrane potential values after 32 hpf may stem from oscillations around the average membrane potential, although we can not rule out differences in dye penetration. In any case, the combined measurement data show a collective increase in depolarization.

To assess the effect of Kcnk5b-mediated decrease in intercellular K^+^ on the membrane potential, we overexpressed *kcnk5b*-mCherry or mCherry at 32 hpf, the time point with the lowest depolarization levels (**Fig 5A–5C**), and then measured membrane potential. We observed that compared to mCherry-expressing fin buds (**Fig 5G and 5I**), overexpression of *kcnk5b*-mCherry significantly increased depolarization (**Fig 5H and 5I**). These results indicated that Kcnk5b activity promotes depolarization.

The observed increase in depolarization caused by *kcnk5b*, a leak channel that should promote hyperpolarization, suggested that other channels whose activity causes depolarization, such as Na^+^ channels, were involved. We therefore examined whether Kcnk5b-mediated depolarization required Na^+^-channel activity. We expressed either control mCherry or *kcnk5b*-mCherry and then treated these fish with the Na^+^-channel inhibitors Vinpocetine (a broad voltage-gated sodium channel inhibitor, including the TTX-insensitive channels) and Dibucane (broad sodium channel inhibition) (**S7E–S7J Fig**). We observed that inhibition of Na^+^ channels decreased depolarization in the fin bud as well as prevented Kcnk5b-induced increase in depolarization (**Fig 5J**). To test whether Na^+^-channel-mediated depolarization is required for *kcnk5b*-enchanced growth, we overexpressed *kcnk5b*-GFP and assess the effect of impairing depolarization using the Na^+^-channel inhibitors. Compared to the enhanced fin bud sizes of control-treated *kcnk5b*-GFP-transgenic fish (**Fig 5M and 5O**), treatment of *kcnk5b*-GFP-expressing fish with the Na^+^-channel inhibitors impaired Kcnk5b-induced fin bud growth (**Fig 5N and 5O**). Together, these data indicated that Kcnk5b activity promotes depolarization via Na^+^ channels and that Kcnk5b-induce depolarization is required for Kcnk5b-enhanced growth.

### IP_3_R-mediated Ca^2+^ release is required for Kcnk5b-induced *shha* expression and fin bud scaling

**Fig 6:**
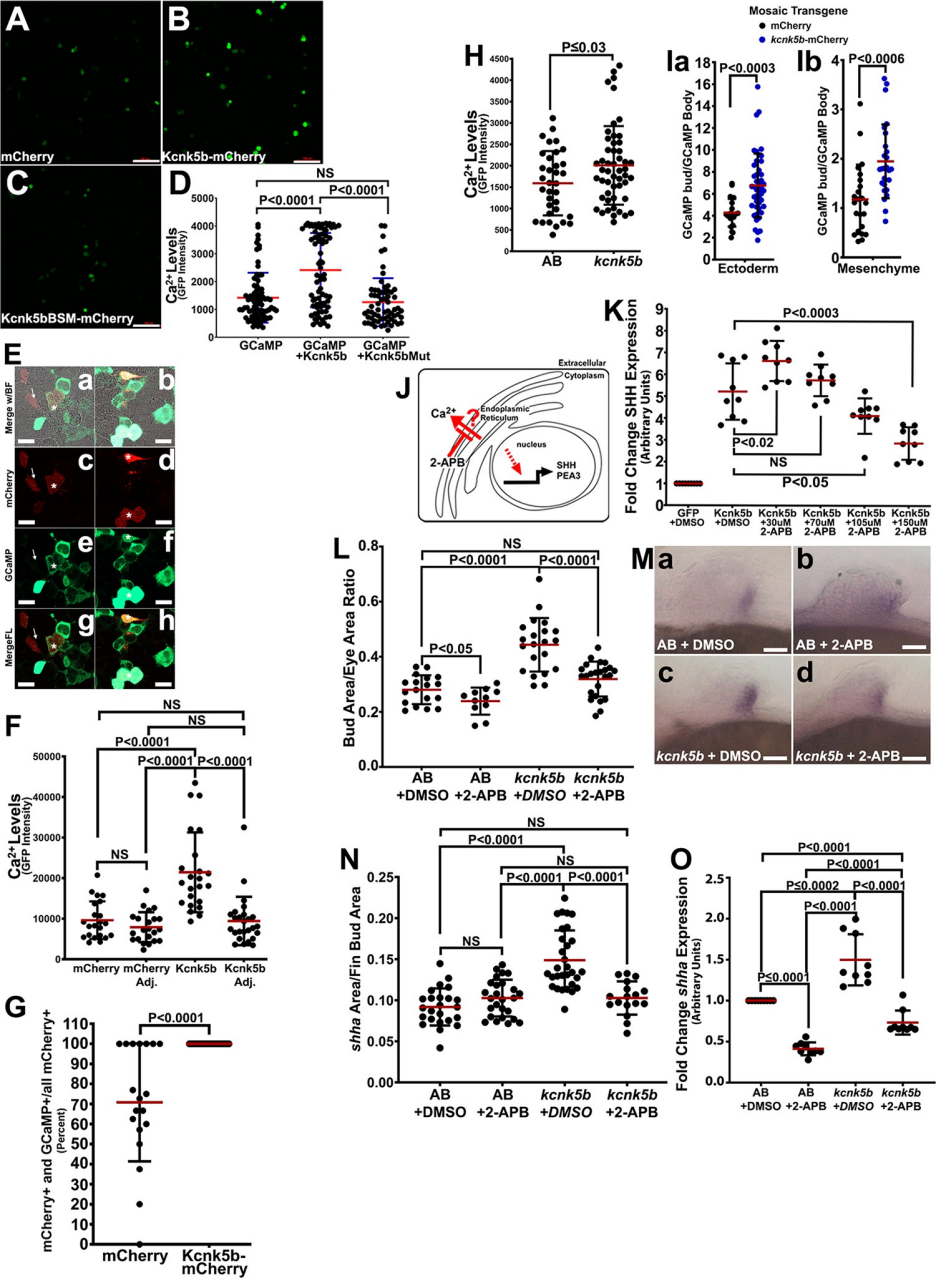
Kcnk5b activity induces IP_3_R-mediated Ca^2+^ release from the ER. **(A)** GCaMP6s fluorescence in HEK293 cells. **(B)** GCaMP6s fluorescence in HEK293 cells expressing Kcnk5-mCherry. **(C)** GCaMP6s fluorescence in HEK293 cells expressing Kcnk5BSM-mCherry. **(D)** Fluorescence measurements of indicated groups. **(E)** Representative confocal images of mCherry-transfected, GCaMP6s-transfected cells showing brightfield with merged fluorescence from mCherry and GCaMP6s (a) or mCherry (c) or GCaMP6s (e) or merged mCherry-GCaMP6s (g), and Kcnk5b-mCherry-transfected, GCaMP6s-transfected cells: brightfield with merged fluorescence from mCherry and GCaMP6s (b) or Kcnk5b-mCherry (d) or GCaMP6s (f) or merged mCherry-GCaMP6s (h). **(F)** Measurements of GCaMP6s fluorescence intensity from the indicated experimental transfection groups. **(G)** Percents of mCherry-GCaMP6s-double positive over the total number of mCherry-positive cells in each group relate the frequency of GCaMP6s-positive cells in the mCherry or Kcnk5b-mCherry groups. **(H)** GCaMP6s fluorescence in the pectoral fin buds of transgenic fish harboring both Tg[Cca.actb:GCaMP6s] and Tg[*hsp70*:*kcnk5b*-mCherry]. **(I)** GCaMP6s fluorescence in embryos harboring the stable transgenic fish line Tg[Cca.actb:GCaMP6s] that mosaically express patches of mCherry or *kcnk5b*-mCherry (designated mCherry+) for the ectoderm (**a**) and mesenchyme (**b**). **(J)** Diagram of IP_3_R inhibition by 2-APB. **(K)** qRT-PCR of SHH expression in HEK293 cells transfected either with GFP or Kcnk5b-GFP after 20-h treatment with 2-APB at the indicated concentrations. **(L)** Assessment of pectoral fin bud size at 48 hpf after 4 h of treatment with 13 μm 2-APB. **(M)** Expression of *shha* in pectoral fin buds of heat-shocked non-transgenic sibling embryos after 4-h treatment with DMSO (**a**) or 13 μm 2-APB (**b**), of heat-shocked transgenic Tg[*hsp70*:*kcnk5b*-mCherry] siblings after 4-h treatment with DMSO (**c**) or 13 μm 2-APB (**d**). **(N)** Pixel area of in situ staining of *shha* in the indicated treatment groups. **(O)** qRT-PCR of *shha* expression in isolated fin buds of the indicated groups. Experiments were repeated 3 or more time (*N* ≥ 3). For cell culture experiments, each repeat contained duplicate or triplicate wells, and 10 or more cells were measured per well. Each data point represents 1 cell (D, F, G). For fin bud fluorescence measurements, we measured 2 or 3 locations in each tissue of 1 fin bud per embryo (H, I). For fin bud area measurements, we measured the area of 1 fin bud and eye per embryo. We measured at least 4 embryos per repeat (L). For the qRT-PCR experiments, we collected 80 fin bud samples per isolation. Three or more isolations were measured. Each isolation was measured in duplicate or triplicate. Each measured value is represented as a data point. *P* values represent statistical analysis by Student’s two-tailed *t* test. *P* values >0.05 are designated as “not significant” (NS). Scale bars equal 100 μm (A–C), 10 μm (E), 0.5 μm (M). Numerical data used in this figure are included in S6 Data. ER, endoplasmic reticulum; hpf, hours post fertilization.

Our observations that K^+^-leak channels increase the expression of important morphogens suggested that the channels are doing so through one or more signaling mechanisms. Since the intracellular accumulation of second messengers is involved in many signaling mechanisms, we assessed whether Kcnk5b activity alters the levels of particular second messengers. We used HEK293 cells for an initial assessment, because we previously observed that Kcnk5b induces SHH in these cells [11], a morphogen important for the development of early fin/limb buds [39]. To determine whether cAMP or cGMP levels change in response to Kcnk5b, we used FLIM with an established FRET-based sensor for each [40,41]. Compared to controls groups of unstimulated cells or cells stimulated with forskolin that produces cAMP (**S8A Fig**) or cells stimulated with SNAP to produce cGMP (**S8B Fig**), Kcnk5b did not significantly alter the intracellular levels of cAMP or cGMP (**S8A and S8B Fig**). We then assessed intracellular Ca^2+^ using the GCaMP6s sensor [42], and we observed that compared to cells transfected with control plasmid (**Fig 6A and 6D**), transfection with Kcnk5b led to significant increases in Ca^2+^ (**Fig 6B and 6D**). An inactive mutant version of the Kcnk5b channel, Kcnk5bMut (**S8C Fig**), also did not increase GCaMP6s activity (**Fig 6C and 6D**). To further assess the relationship between Kcnk5b and intracellular Ca^2+^, we transfected cells with GCaMP6s and either with Kcnk5b-mCherry or with mCherry. We subsequently cultured the cells for 24 h, FACS sorted both transfection groups for mCherry fluorescence, and then plated each group of sorted cells at 100% confluency (**Fig 6Ea and 6Eb**). mCherry-transfected cells that did not consistently display GCaMP6s fluorescence (**Fig 6Ea, 6Ec, 6Ee, 6Eg arrow versus asterisk and 6F**), while Kcnk5b-mCherry+ cells always showed high GCaMP6s activity (**Fig 6Eb, 6Ed, 6Ef, 6Eh asterisks and 6F**). We observed this phenomenon 100% of the time in all confocal images (**Fig 6G**). Together, these results indicate that Kcnk5b activity promotes a rise in intracellular Ca^2+^.

To determine whether Kcnk5b has the same effect on intercellular Ca^2+^ levels in vivo, we generated double-transgenic fish that harbored Tg[*hsp70*:*kcnk5b*-mCherry] and a CGaMP6s Ca^2+^ reporter under the control of the β-actin promoter Tg[Cca.actb:GCaMP6s] [43]. We assessed intracellular Ca^2+^ in the fin buds of heat-shocked transgenic *kcnk5b*-mCherry embryos at 48 hpf and their non-transgenic siblings as controls. We observed that Ca^2+^ levels were higher in *kcnk5b*-mCherry compared to non-transgenic embryos (**Fig 6H**). We observed similar results when we assessed mosaic embryos harboring the stable transgenic fish line Tg[Cca.actb:GCaMP6s] that mosaically express patches of mCherry or *kcnk5b*-mCherry (**Fig 6Ia and 6Ib**). Together, these data indicated that Kcnk5b normally promotes the increase in intracellular Ca^2+^ levels in vivo, and they suggested Ca^2+^ mediates the growth-inducing effect of Kcnk5b. Increases in Ca^2+^ caused by the decrease in intracellular K^+^ could occur from extracellular sources via Ca^2+^ channels in the plasma membrane and/or from intracellular sources such as the endoplasmic reticulum (ER) [44]. To determine the importance of these 2 sources, we tested whether inhibiting release of Ca^2+^ from each source impaired Kcnk5b-induced transcription of SHH in HEK293 cells. After inhibiting T- and L-type Ca^2+^ channels on the plasma membrane (**S8D Fig**), we did not detect a significant effect on Kcnk5b-induced SHH transcription (**S8E and S8F Fig)**. However, after inhibiting IP_3_R-mediated release of Ca^2+^ from the ER (**Fig 6J**), we observed decreased Kcnk5b-induced SHH transcription dose-dependently (**Fig 6K**).

To determine whether IP_3_R-mediated Ca^2+^ release was required for the Kcnk5b-enhanced growth of pectoral fin buds, we inhibited IP_3_R activity for 4 h and assessed bud growth in heat-shocked non-transgenic and transgenic Tg[*hsp70*:*kcnk5b*-GFP] siblings. We observed that the IP_3_R inhibitor blocked the growth phenotype caused by expression of *kcnk5b* at 48 hpf (**Fig 6L**). This decrease in growth was associated with the impairment of Kcnk5b-induced increase of *shha* expression domains (**Fig 6M and 6N**) and qRT-PCR (**Fig 6O**).

**Fig 7:**
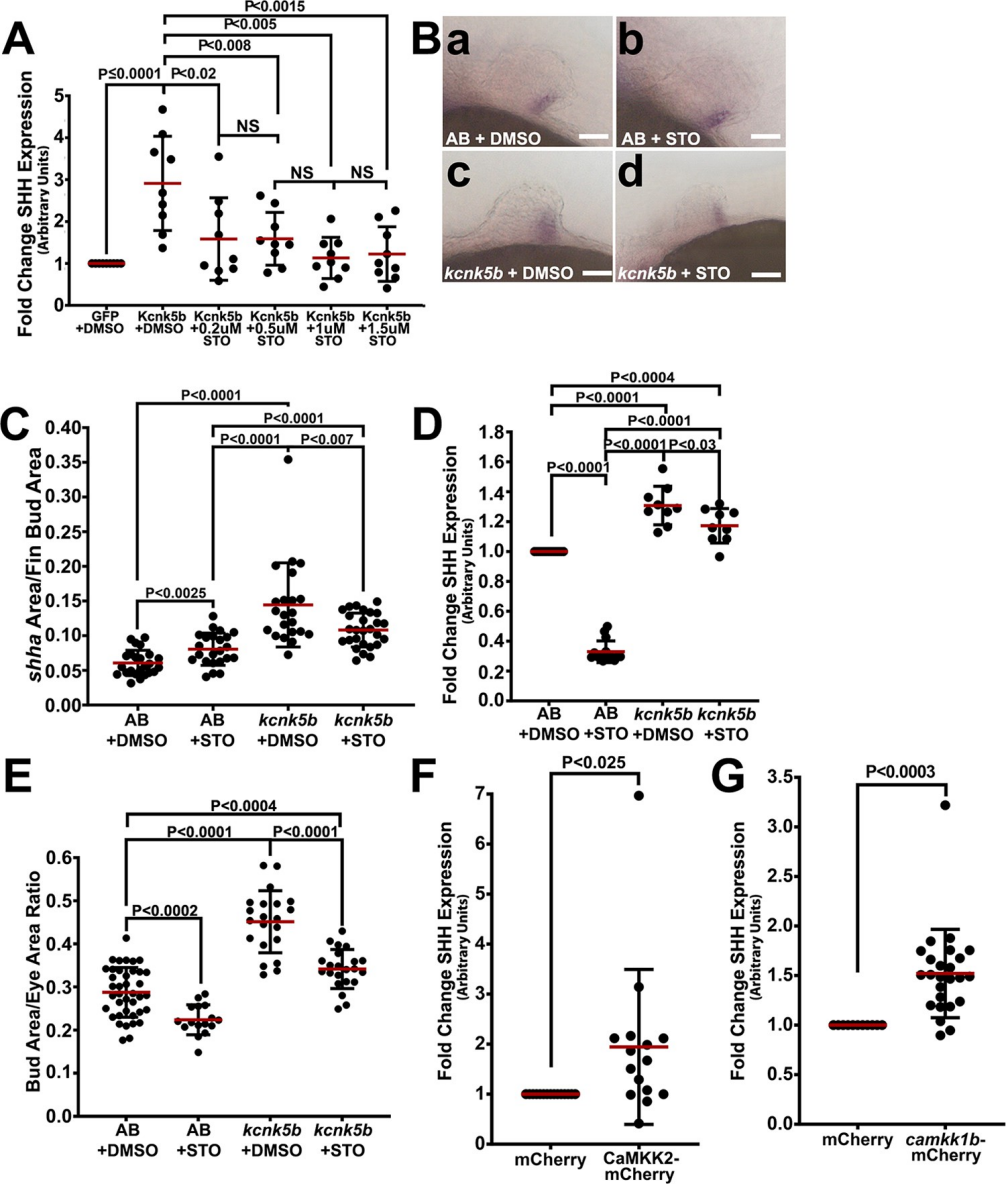
Kcnk5b requires CaMKK for growth and SHH/*shha* expression. **(A)** SHH expression in HEK293 cells transfected with GFP or Kcnk5b-GFP after 20-h treatment at the indicated concentrations of the CaMKK inhibitor STO-609 at the indicated concentrations. **(B)** Expression of *shha* in pectoral fin buds from heat-shocked non-transgenic siblings after 6-h treatment with DMSO (**a**) or 24 μm STO-609 (**b**) and from transgenic Tg[*hsp70*:*kcnk5b*-mCherry] siblings after treatment with DMSO (**c**) or STO-609 (**d**). **(C)** The ratios of each in situ staining area of *shha* to the area of its corresponding fin bud for the indicated groups. **(D)** qRT-PCR of *shha* expression in isolated fin buds in the indicated control and experimental groups. **(E)** Graphed assessment of fin-bud-area-to-eye-area measurement ratios. **(F, G)** Difference in SHH expression in HEK293 cells transfected with mCherry or human CaMKK2-mCherry (F) or *camkk1b*-mCherry (G). Experiments were repeated 3 or more time (*N* ≥ 3). For cell qRT-PCR experiments, each RNA isolation per well was measured in duplicate or triplicate. Each measured value is represented as a data point (A, F, G). For fin bud fluorescence measurements, we measured 2 or 3 locations in each tissue of 1 fin bud per embryo. For fin bud area measurements, we measured the area of 1 fin bud and eye per embryo. We measured at least 4 embryos per repeat (E). For fin bud qRT-PCR experiments, we collected 80 fin bud samples per isolation. Three or more isolations were measured. Each isolation was measured in duplicate or triplicate samples (D). Each measured value is represented as a data point. *P* values represent statistical analysis by Student’s two-tailed *t* test. *P* values >0.05 are designated as “not significant” (NS). Scale bars equal 0.5 μm (B). Numerical data used in this figure are included in S7 Data.

The importance of Ca^2+^ release for *shha* expression and fin bud growth indicated that one or more Ca^2+^-dependent kinases were involved. Therefore, we tested which of the Ca^2+^-activated CaM kinases were required for Kcnk5b-enhanced expression of SHH in HEK293 cells. While we observed no significant effects by inhibiting CaMKII and CaMKIV (**S9A Fig**), we did observe that inhibition of CaMKK impaired Kcnk5b-enhanced SHH expression (**Fig 7A**). We then tested whether inhibiting CaMKK in the fin buds has similar effects on *shha* expression in vivo. We observed that inhibiting CaMKK decreased the expression of *shha* by in situ (**Fig 7B and 7C**) and by qRT-PCR (**Fig 7D**). Inhibiting CaMKK also impaired Kcnk5b-enhanced growth (**Fig 7E**). Furthermore, overexpressing the human CaMKK2 (**Fig 7F**) or the zebrafish *camkk1b* (**Fig 7G**) was sufficient to increase SHH expression in HEK cells. Together, these results indicated that Ca^2+^ is required for Kcnk5b-induced transcription of *shha* and that CaMKK is an important part of Ca^2+^ regulation of *shha* expression and pectoral fin bud growth.

## Discussion

### K^+^-channel activity as a mechanism for regulating growth of vertebrate fin/limb buds

The fish mutants *longfin*, *another long fin*, *kcn13j*, and *rapunzel* demonstrate that increasing the expression or the posttranslational activity of a K^+^ channel (Kcnh2a, Kcn13j, Kcnk5b, or Kcc4a) induces allometric growth of adult fin [9,10,12,13]. The observations that different K^+^ channels enhance growth support the conclusion that changes in intracellular K^+^ is what alters scaling. While Kcnk5b and other K^+^-leak channels reduce intracellular K^+^ levels [45], Kcn13j K^+^-rectifying channels facilitate K^+^ entry into the cells [46]. An explanation for why channels that release K^+^ and a channel that restores intracellular K^+^ both promote growth is current evidence that suggests that K^+^ leak channels act directly in tissues of the fin, while the K^+^-rectifying channel acts in the dermomyotome of somites to affect the later growth of adult fins [13]. The value of our in vivo FLIM measurements of intracellular K^+^ is that they provide direct evidence of a decrease in relative intracellular K^+^ levels in pectoral fin bud tissues during outgrowth (**Fig 1D–1I**), which supports the conclusion that growth of the pectoral fin buds involves an overall reduction of intracellular K^+^ in bud tissues. This conclusion is also supported by multiple observations: (1) the enhanced proportions from transgenic overexpression of different K^+^-leak channels (**Fig 2**); (2) the expression of Kcnk5b during the fin bud outgrowth and its down regulation as fin bud development ceases (**Fig 3E and 3F**); (3) the smaller fin bud size in CRISPR-targeted *kcnk5b* embryos (**Fig 4A–4E**); (4) the decrease in intracellular K^+^ by RA treatment (**Fig 3A–3D**); (5) the enhanced bud size (**Fig 3K–3M**); (6) the decreased K^+^ (**Fig 3N**) from *rcan2* overexpression (which enhances growth); (7) *rcan2*-induced enhanced growth requires *kcnk5b* (**Fig 4**); and (8) CRISPR targeting of *rcan2* both decreased bud size and increased K^+^ (**Fig 3S and 3T**). We also observed *rcan2* expression and its similar effects on scaling and regulating intracellular K^+^ in adult caudal fins.

The endogenous expression profiles of *kcnk5b* (**Fig 3E and 3F**) and *rcan2* (**Fig 3G**) show that both are present during the growth of the fin bud. Rcan2 is also present in the adult fin blastema where it can regulate Kcnk5b and enhance growth (**S5I–S5K Fig**). While the accumulated data indicate that the expression of *rcan2* coincides with *kcnk5b* in fin bud and fin growth, between 32 hpf and 34 hpf, we observed an increase in intracellular K^+^ (**Fig 1E**) despite the presence of *rcan2* and *kcnk5b*. We posit that there is another mechanism regulating Kcnk5b or another K^+^ channel or K^+^ pumps that facilitates the increase in K^+^. We propose that the increase in K^+^ between 32 hpf and 34 hpf (**Fig 1E**) explains the pause in bud growth during this period (**Fig 1F**). We suspect the *kcnk5b* expression at 56 hpf (**Fig 3Fb**) means that this channel is involved in other phenomena such as early tissue differentiation, while the decreased *rcan2* at 56 hpf (**Fig 3Gd**) indicates that the RA-Rcan2 mechanism no longer promotes Kcnk5b activity and, consequently, intracellular K^+^ levels increase.

Zebrafish fin bud development occurs within a 24-h period, by which time it has transitioned into finfold growth, which initiates out of the distal-most region of the bud to form the pectoral fins of the larval fish. Because this transition between the conserved vertebrate fin/limb bud developmental stage, we primarily focused our characterizations to 48 hpf in order to define the contribution of intracellular K^+^ regulation in the scaling of the conserved embryonic bud structure. Thus, the changes in fin bud size caused by knockout or overexpression of *kcnk5b* or *rcan2* are not large, but they are biologically significant. We attribute the variance that we observed at the measured time points to natural variations in growth within the narrow window of pectoral fin bud development.

There are several signaling centers/zones within the developing fin buds [22], yet we observed few distinctions in the distribution of relative intracellular K^+^ levels in the early buds (**Figs 1G–1I and S3**). We propose this is due to gap junctions. Gap junctions are important for limb bud development and fin growth [47–49], and the shared distribution of relatively similar K^+^ levels via gap junctions can explain the coordinated regulation of each morphogen throughout the bud. We observed that the primary difference in relative levels of intracellular K^+^ exists between the mesenchyme and the ectoderm (**Fig 1D and 1E**). These differences may be due to an ECM barrier between the tissues, fewer gap-junctional connections and/or differences in molecular signals that influence K^+^ channel expression/activity. Because cells use intracellular K^+^ to control resting membrane potential [45,50], any differences in K^+^ levels between these tissues suggest that they have distinct electrophysiological properties.

In regards to the in vivo electrophysiology, since K^+^-leak channels generally result in the outward flow of K^+^ ions, this should hyperpolarize the membrane potential as the cytoplasmic side becomes more negatively charged from the outflow of positive-charged ions. However, we observed that depolarization increases during outgrowth (**Fig 5A–5F**) while intracellular K^+^ decreases (**Fig 1E**). Furthermore, when we overexpress *kcnk5b* in the fin buds, we increased depolarization (**Fig 5G–5I**). We propose that the increased K^+^-channel activity first hyperpolarizes the membrane potential, and this increase in membrane potential immediately activates Na^+^ channels to depolarize the cells. This hypothesis fits with our findings that we impair the increase in depolarization (**Fig 5J**) as well as Kcnk5b-enhanced growth with Na^+^ channel inhibitors (**Fig 5K–5O**). We posit that the rise in intracellular K^+^ by 56 hpf directly facilitates the observed depolarization from the accumulation of K^+^ at the membrane.

### Integration of Kcnk5b into a mechanism of fin bud development and growth

The coordinated changes in intracellular K^+^ (**Fig 1**) argue that there are common mechanisms regulating K^+^ dynamics. We identified retinoic acid-regulated signaling as one such mechanism (**Fig 3**), since RA was sufficient to decrease intracellular K^+^ in the ectoderm and mesenchyme (**Fig 3A–3D**). We previously showed that calcineurin inhibition induces allometric growth of adult fins [51] by increasing Kcnk5b activity [11]. Our findings that *kcnk5b* and the endogenous calcineurin inhibitor *regulator of calcineurin 2* (*rcan2*) are present in pectoral fin buds (**Fig 3E–3G)** and that both are important in determining the size of the fin buds (**Figs 3 and 4**) along with our finding that RA increases *rcan2* expression (**Fig 3H–3J**) suggest a mechanism that involves calcineurin-mediated antagonism of Kcnk5b activity to scale the buds (**Figs 3 and 4**). Our results support this model: (1) overexpression of the calcineurin inhibitor *rcan2* decreased intracellular K^+^ and increases growth of fin buds (**Fig 3M and 3N**) and of adult fins (**S5AA and S5BB**); (2) overexpression of *kcnk5b* (which decreases intracellular K^+^) enhanced the developmental transcription and growth of the fin buds (**Fig 2B–2W**) and of adult fins [11]; and (3) a reduction in Kcnk5b activity by knockout or by point mutation impairs *rcan2*-induced bud growth (**Fig 4F–4L**).

The expression patterns of *rcan2* and *kcnk5b* were primarily in the mesenchyme of the buds (**Fig 3E–3G**). We interpreted these findings to mean that intracellular K^+^ is higher in the ectoderm because of very low *rcan2* and *kcnk5b* levels or because of the absence of these 2 genes and the presence of another RA-regulated K^+^ channel in the ectoderm. Differences in the expression of K^+^ channels between the ectoderm and the mesenchyme can explain their differences in K^+^ levels (**Fig 1D and 1E**). RA does regulate the expression other morphogens and growth factors in fin/limb buds, so the linked changes in intracellular K^+^ between the ectoderm and mesenchyme can be coupled through alternative RA-mediated activities that affect the activity or expression of other K^+^-channels in the ectoderm.

The coordinated regulation of intracellular K^+^ had some specificity, since RA did regulate intracellular K^+^ while a different nuclear hormone (thyroid hormone) did not have any effect (**S5C Fig**). We interpret these results to mean that TH3 signaling does not directly regulate the Kcnk5b-mediated growth mechanism even though thyroid hormones in other biological contexts can induce Rcan2 and/or promote growth [52,53]. We propose that any thyroid-mediated growth occurs via another molecular mechanism and not this electrophysiological one. Alternatively, there are endogenous factors present that limit the effects of this hormone, since it can promote metamorphosis [54], which needs occur later as the fish ends its larval stage [55]. Another possibility is that in early embryonic fin bud and homeostatic growth of the adult fin, the binding sites that thyroid nuclear hormone receptors require are either not accessible or are absent from the regulatory regions of the zebrafish *rcan2*. Considering the importance of thyroid hormone-mediated expression of *rcan2* in the differentiation of osteoblasts [56], and considering that growth of the fin bud and regenerating fin needs cell proliferation before tissue differentiation, our findings may highlight one of these possibilities.

### K^+^-leak channels scale using Ca^2+^

An important question is how do K^+^-leak channels scale fin buds. Part of the answer involves IP_3_R-induced Ca^2+^ release from the ER. However, our previous findings showed that inhibition of the Ca^2+^-dependent phosphatase calcineurin increases the activity of Kcnk5b [11]. While this finding appears incongruent with our current finding that IP_3_R-mediated increase in intracellular Ca^2+^ is required for growth, the observed changes in SHH expression from our IP_3_R inhibition experiments (**Fig 6K**) offer an explanation: we observed that milder inhibition of IP_3_R (**Fig 6K**, 30 μm 2-APB**)** enhances Kcnk5b-induced expression of SHH, which we posit as reducing the pool of Ca^2+^ needed for calcineurin’s inhibition of Kcnk5b, while greater IP_3_R inhibition decreases SHH (**Fig 6K, ≥**105 μm 2-APB, **and 6M–6O**) by impairing other Ca^2+^-dependent enzymes needed for SHH expression, such as CaMKK (**Fig 7**).

Our findings are in line with other evidence that point to the importance of Ca^2+^ in the growth of appendages. The L-type Ca^2+^ channel Ca_v_1.2 can cause syndactyly, in which the bones of the digits improperly fuse, when mutations cause this channel to stay open longer and increase Ca^2+^ in the sarcoplasmic reticulum (muscle ER) [57]. Conversely, knock-out of Ca_v_1.2 in the limb mesenchyme leads to shorter limbs due to impaired skeletal development [58]. In *Drosophila* wing discs, disruption of proteins that maintain ER Ca^2+^ stores—such as the Serca2 Ca^2+^ pump, the Orai Ca^2+^ channel in the plasma membrane, the Best2 Cl^-^ channel in the ER membrane, or Stim, a scaffold protein that colocalizes Orai and Best2—leads to mispatterned, stunted adult wings [59].

The ER has the largest intracellular Ca^2+^ store, and the release of Ca^2+^ from the ER into the cytoplasm and active pumping of Ca^2+^ back into the ER occurs at regulated frequencies to generate oscillating cytoplasmic waves [60]. Ca^2+^ oscillations coordinate mesenchyme cell movement in the developing buds of feathers [61], and a similar phenomenon may regulate the growth of the fin buds [12,62]. Our observation that K^+^ is shared between cells (**Fig 1K–1M**) prompts questions about which ions are involved in the coordinated control of genes and allometric growth (**Fig 2**). We propose that the sustained transcription that is needed for prolonged allometric growth involves a sustained stimulus. Based on our observations, the decrease in intracellular K^+^ remains relatively constant as the fin bud grows (**Fig 1E and 1F**). Decreases in K^+^ could increase intracellular Ca^2+^ by increasing the amplitude or the duration of Ca^2+^ release from the ER. We posit that it enhances duration, since CaMKK activity is required, and this enzyme needs sustained durations of Ca^2+^ that achieve its two-step activation process: Ca^2+^ must be present long enough to interact with calmodulin and then allow the Ca^2+^-calmodulin complex to activate CaMKK. We conclude that K^+^ is an overarching long-term signal that adjusts IP_3_R-mediated Ca^2+^ release to regulate growth.

### Intracellular K^+^ in coordinated regulation of morphogens during development

An important question is why use K^+^ channels to scale structures. One possibility is that specific K^+^ channels have interactions with specific growth-regulating receptors. The Thromboxane receptor interacts with the K^+^ miniK channel to regulate the channel’s activity [63]. Trimeric GPCRs and other membrane-associated signaling molecules interact with channels to impact channel function [64–66]. However, we currently believe that specific channel–receptor interactions do not explain our observations, because the similar allometric growth phenotypes can be induced by different K^+^ channels (**Figs 2C and S4D–S4F**) [9–12] that likely do not interact with the same growth-promoting receptors. A second possibility is that changing intracellular K^+^ levels alters the electrophysiology of cells to promote growth. Changes in intracellular K^+^ are known to alter the electrophysiological properties of cells [45], and such changes could alter the activities of pro-growth transmembrane receptors or membrane-associated signaling cascade components without direct interactions. A related possibility is that intracellular K^+^ is distributed throughout the cytoplasm, so changes in intracellular K^+^ could influence factors beyond the plasma membrane, such as the IP_3_R. Changes in K^+^ could alter activities of other transduction cascade components by influencing ionic interaction with charged amino acids in proteins or between them. Such a K^+^-mediated regulatory mechanism would not necessarily be an on-off switch, but could be an amplifier that augments the activities of signaling components that enhance the expression of existing developmental signals (**Fig 2D–2W**). The ionic “amplifier” mechanism fits with the observation that CRISPR-targeting of *kcnk5b* did not prevent growth of the fin buds; instead, it just reduced their proportions (**Fig 4A–4E**).

### K^+^ channels in the broader context of development and disease

In a broader context of development, there are several findings that link different K^+^ outward-flow channels to human syndromes that harbor limb defects. The voltage-gated KCNH1 (Kv10.1), the two-pore channel KCNK4 (TRAAK/TREK) and the small-conductance Ca^2+^-activated KCNN3 (SK3/KCa2.3) are all K^+^ channels whose increase in activities can lead to hypoplasia/aplasia of the distal phalanges, as well as lead to alterations in cranial-facial features and neuropathies [8,67–70]. Conversely, mutations that impair KCNK9 activity produce bilateral hand contractures and talipes equinovarus feet [71,72]. Comparison between these findings and our findings shows the diversity in the physiological activities of K^+^ channels that decrease intracellular K^+^. While the different phenotypes can manifest from growth defects, we suspect that the differences are due to tissue-specific activities, since even the same channel in different cells can facilitate the transcription of different genes [11].

In addition to channels involved in outward K^+^ flow, inwardly rectifying K^+^ channels are important for physiology. While many discoveries link their importance to behavioral phenomena [73], mutations in KCNJ6 (GIRK2) or KCNJ13 (Kir7.1) result in severe cranial-facial malformations along with intellectual disabilities [74,75]. However, it is unclear how many of the detrimental phenotypes are due to KCNKJ6’s dysfunctional K^+^ flow, since the only characterized mutation that causes the loss of K^+^ selectivity, also gains Ca^2+^ permeability [76]. In regards to growth, increased expression of Kcnj2 is linked to hypoplasia of distal digit structures [77], and increased activity of another inward rectifying channel Kcnj13 has also been linked to enhanced growth of the adult fins, although it appears to initiate this defect via its embryonic activities [13].

K^+^ channels are also linked to tumor formation and cancer. Several different cancers harbor up-regulated expression and/or activity of K^+^-leak channels. Expression and activity of KCNK5 is up-regulated in some breast cancer cell lines. Signaling from Estrogen Receptor-α (ERα) has been found to promote breast cancer [78]. ERα signaling can up-regulate the expression of KCNK5, and blocking KCNK5 activity impairs the cell proliferation caused by activated ERα [79]. KCNK9 expression is also elevated in a number of breast cancer tumors, and experimentally overexpressing this channel promotes tumor formation in vivo [80]. A link between KCNN4 up-regulation and cell proliferation has been shown in smooth muscle cells of the vasculature [81]. Several cancer cell lines harbor elevated expression of KCNH1, and increasing this channel’s expression in cells can transform them into cancer-like cells [82]. KCNH5 is highly up-regulated in medulloblastomas, and targeted down-regulation of this channel reduced blastoma growth in vivo [83].

Tumor tissues and cell lines can also have reduced expression of the potassium channel regulator KCNRG [84], which reduces K^+^ currents across the plasma membrane [85,86] and reduces cell proliferation [84]. It is hypothesized that KCNRG reduces K^+^ channel expression, thereby limiting the release of intracellular K^+^ through in the plasma membrane [86]. Along these lines, decreased expression of Kv1.3, a rectifying K^+^ channel involved in restoring intracellular K^+^ levels, was observed to be down-regulated in breast adenocarcinoma cell line MCF-7 [87].

Our data are consistent with other finding that show that K^+^ channels that reduce intracellular K^+^ to promote growth. However, ours and others’ findings also indicate that K^+^ channel activity regulates more than cell proliferation, since K^+^ channel activity regulates the transcription of several morphogens in different regions of the developing buds and adult fins [11,88]. If Kcnk channels acted solely as oncogenes in the fin buds and adult fins, then they would likely produce tumors rather than foster coordinated allometric growth of the entire anatomical structures [9,11] or reversed polarity of regenerative outgrowth in adult fins [88]. Furthermore, the coordinated patterning alterations in craniofacial and limb structures by defective K^+^ channels also suggest that these channels regulate more than cell proliferation.

The findings that individual dysfunctional K^+^ channels produce compound defects suggest that K^+^ channels have diverse activities via different molecular mechanisms. However, in the majority of cases, it remains unclear how these channels are involved. Ultimately, to get a better understanding for how specific K^+^ channels regulate the formation and growth of specific tissues and what regulates their channel contributions, future tissue-specific targeting of specific K^+^ channels is needed along with in vivo assessments of their electrophysiological activities.

**Fig 8:**
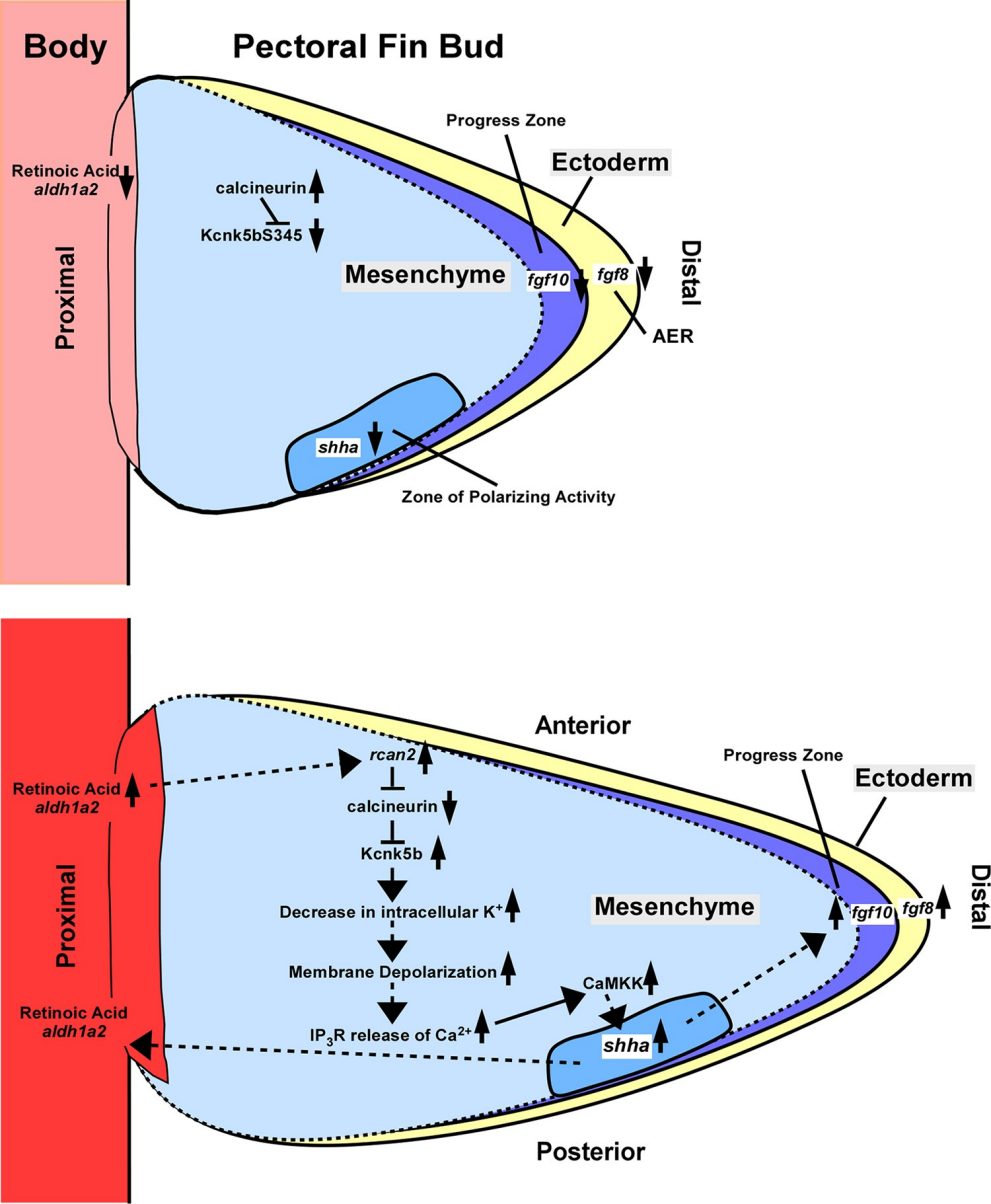
Model. RA signaling induces the transcription of *rcan2*, an endogenous inhibitor of calcineurin, to alleviate calcineurin inhibition of Kcnk5b channel activity. Kcnk5b decreases intracellular K^+^ levels in the mesenchyme that increase depolarization to promote IP_3_R-mediated Ca^2+^ release from the ER. The increase in intracellular Ca^2+^ activates CaMKK, and both are required for the increased transcription of *shha* and enhanced growth of the pectoral fin buds by Kcnk5b. ER, endoplasmic reticulum; RA, retinoic acid.

In summary, we propose that RA regulates intracellular K^+^ via an Rcan2-mediated increase in Kcnk5b activity to promote fin bud growth. The resulting decrease in intracellular K^+^ levels causes IP_3_R-mediated Ca^2+^ release that enhances *shha* transcription and other morphogens either directly or through Shha production (**Fig 8**). Thus, our observations integrate K^+^-channel-regulated scaling into known molecular controls of fin/limb bud development. While our proposed mechanism describes how one K^+^-leak channel is linked into developmental signals, details of the mechanisms still need to be defined. Given the diversity of ion channels and the importance of K^+^ in regulating the electrophysiological properties of cells, our findings may have broader implications in organ scaling and diseases that are caused by the loss of proportional growth. Continued work will determine the extent and diversity in how the electrochemical properties of cells interface with the molecular controls that govern organ development and proportional growth.

## Materials and methods

### Cloning

Constructs were designed either by standard restriction enzyme or by homologous recombination methods. KIRIN1 was synthesized (Genewiz) and cloned into MCS region of pcDNA6-myc-6xHIS-tag plasmid (Invitrogen, V22120) or pBluescript (VWR, 95040–830) harboring the *hsp70* zebrafish promoter by 2 miniTol2 sites (transgenic vector). We cloned kcnk5b-GFP, GFP, *kcnk10a*-GFP, kcnk5b-mCherry, CaMKK2-mCherry, *camkk1b*-mCherry, and mCherry, *rcan2*-mCherry into pcDNA-myc-6xHIS-tag or pBluescript II vector (Invitrogen AM1344) for expression in cells or fish. We cloned kcnk5b-GFP, *rcan2*-mCherry and mCherry into pXT7 vector (Addgene, #32995) for mRNA injection.

### Zebrafish husbandry

AB strain fish were raised in 10 L tanks with constantly flowing water, 26°C standard light-dark cycle [89] HaiSheng aquarium system. Fish embryos and larva were raised in 1× E3 medium (5 mM NaCl, 0.17 mM KCl, 0.33 mM CaCl2, 0.33 mM MgSO4, 10%–5% Methylene Blue) until 10 to 12 dpf, then transferred to aquarium water tanks to grow. Transgenic lines established by screening for GFP, CFP, and mCherry expression after heat shock. Experiments used male and female fish equally. In general, embryos were immobilized by adding Tricane (MESAB) to E3 at a final concentration of 4 μm for 2 min (or 200 nM blebbistatin for 30 min where indicated) and then embedded in 1% low-melting agarose for 10 to 30-min imaging sessions. Where indicated, embryos were immobilized with Blebbistatin in 200 nM final concentration for 30 min instead of Tricane.

### Ethics statement

Fish experiments were compliant to the general animal welfare guidelines and protocols (#20200903003) approved by legally authorized animal welfare committee, ShanghaiTech Animal Welfare Committee.

### Generation of transgenic lines

Zebrafish embryos were collected at one-cell stage for plasmid injection. Transgenic lines harboring the *hsp70*:KIRIN1, *hsp70*:GFP *hsp70*:*kcnk5b*-GFP, *hsp70*:*kcnk5b*-mCherry, *hsp70*:mCherry, *hsp70*:*kcnk10a*-mCherry, or *hsp70*:*rcan2*-mCherry transgene plasmids were created by injecting 300 μg/μl of each construct together with mRNA of Tol2 transposase [90]. Positive embryos were screened after 37°C heat shock for 1 h. Positive embryos were brought up to adult fish and screened by crossing with wild-type fish. Then, the positive F1 and successive generations were screened by heat-shocking and identifying fluorescence expression. Adult fish were 6 months to a year old, unless indicated differently in the text. Embryonic stages that were used are indicated in the text.

### Growth measurements

Embryos were first staged by the hour until 30 hpf and subsequently by the published head to body angles as the embryos continued develop [30 hpf (angle 85°), 32 hpf (90°), 34 hpf (97.5°), 36 hpf (105°), 38 hpf (110°), 40 hpf (115°), 44 hpf (125°), 46 hpf (130°), until 48 hpf (135°) [91]. Embryos were subsequently measured every 2 h. At each designated stage, the embryos were imaged using a Zeiss Stereoscope. The fin bud areas, eye areas, and otic vesicle areas were measured using Zen 3.4 (Blue edition) software by outlining each anatomical structure with the program’s contour tool and measuring the encompassed area.

### Heat-shock induction of transgenes

Parent fish of heat-shock-driven transgenic lines were either outcrossed (*rcan2*, *kcnk5b*) as hemizygosity to same-strain wild-type fish or in-crossed (KIRIN1, GCaMP6s) for homozygosity. Progeny were collected in 1xE3 and raised at 28°C. All embryos were screen for their respective transgenes by a single heat shock at 37°C for 1 h at 24 hpf and selected for fluorescence. We determined that transgene expression was highest at 6 h post heat shock, so we designed our heat shock regimens accordingly. For FLIM imaging, transgenic lines Tg[*hsp70*:KIRIN1] embryos were heat shocked for 1 h at 37°C imaged 4 to 6 h later for each time point of the time course experiments. Tg[*hsp70*:*kcnk5b*-GFP] embryos and non-transgenic siblings were heat shocked twice: once at 36 hpf and again at 42 hpf for 1 h at 37°C to maximize target gene expression and then collected for the in situ and qRT-PCR experiments at 48 hpf. For pectoral fin bud growth experiments, Tg[*hsp70*:*kcnk5b*-GFP] and Tg[*hsp70*:*rcan2*-mCherry] embryos and non-transgenic siblings were heat shocked at 36 hpf and again at 42 hpf and then imaged at 48 hpf to maximize their expression over a significant portion of the pectoral fin bud growth period. For depolarization inhibitor treatments and subsequent DiSBaC_2_(3) experiments, Tg[*hsp70*:*kcnk5b*-GFP] embryos and non-transgenic siblings were heat shocked at 26 hpf and imaged at 32 hpf. For depolarization inhibitor treatments and subsequent measurements of fin bud size, Tg[*hsp70*:*kcnk5b*-GFP] embryos and non-transgenic siblings were heat shocked at 32 hpf once to avoid the heart problems induced by Na^+^ channel inhibition. For adult Tg[*hsp70*:*rcan2*-mCherry] and non-transgenic fish during caudal fin regeneration, the whole fish was incubated in 38.5°C aquarium water for 12 min once a day for the duration of each specific experiment.

### In situ hybridization

mRNA probes were made from RT-PCR products isolated from 2 dpf zebrafish embryos or caudal fins from 6-month-old adults. The primer sequences for generating the published probes [11,92] are listed in Table 1. mRNA probes were made from RT-PCR products from fish larva (5 dpf) or regenerating (3 dpa) adult caudal fins. In vitro transcription reagents were from Promega. Embryos and tail fins were incubated in 4% PFA in 1xPBS at 4°C overnight with gentle rocking. Samples were dehydrated by incubation for 15 min in methanol at room temperature and then incubated in 100% methanol for ≥2 h at −20°C. Samples were then rehydrated using the reversed dehydration series of methanol/1xPBS solutions (75%, 50%, 25%). Samples were then incubated more than 4× in 1xPBS to remove all methanol, and subsequently incubated in 10 μm Proteinase K for 10 min at RT. Samples were then incubated 20 min in 4% PFA/1xPBS to inactivate the Proteinase K. Samples were incubated in 1xPBS 6× 10 min to remove the PFA, then incubated in pre-hybridization buffer for 3 h at 65°C. Samples were subsequently incubated in the hybridization solution containing 200 ng/ml of each mRNA probe ≥14 h at 65°C. Samples then were washed with successive wash steps to remove unbound probe and prepare for antibody incubation: once 2xSSC/75% deionized formalin at 65°C, once 2xSSC/50% deionized formalin at 65°C, once 2xSSC/25% deionized formalin at 65°C. 2xSSC at 65°C, twice 0.2xSSC at 65°C, 6 times 1xPBST (1xPBS with Tween-20), once in blocking solution [2% bovine albumin (Sigma-Aldrich, A3294-100G), 2% Sheep Serum (Meilunbio, M134510)] at RT for 4 h. Samples were incubated with Anti-digoxigenin-AP Fab Fragment (Sigma-Aldrich, 11093274910, RRID: AB_514497) in blocking solution ≥14 h at 4°C. Samples were then washed 6 times with 1xPBST, subsequently incubated in [0.1 M Tris-HCl (pH 9.5), 0.1 M NaCl, 0.05 M MgCl_2_] 3 times for 30 min, and then in Nitro Blue Tetrazolium (Sigma-Aldrich, N6639-1G) and 5-bromo-4-chloro-3-indolyl phosphate (Sigma-Aldrich, 136149) in [0.1 M Tris-HCl (pH 9.5), 0.1 M NaCl, 0.05 M MgCl_2_] at RT ≥8 h. Samples images under VS120-S6-W (OLYMPUS). For signal areas, images of the *shha* in situs were transferred to Fiji ImageJ (RRID SCR_003070). The stained regions were traced using “free ROI” and then quantitated using the “measure” function under the “analysis” menu to calculate the number of pixels contained in the stained area. For signal intensity analysis, the signal region and adjacent unstained regions were measured by “free ROI” selection and the mean intensity pixel values were determined from the “measure” function under the analysis menu by subtracting the mean value of the unstained region from the mean value of the signal region for each fin bud.

**Table 1:** Materials table.

Reagents and resources	Source	Identifier
**Antibodies**		
Anti-Rcan2 Rabbit Polyclonal Antibody	Proteintech	12900-1-AP-50UL
Anti-digoxigenin-AP Fab Fragment	Roche	11093274910,
**Chemicals, peptides, and recombinant proteins**		
DMEM medium	Gibco	1199506
Bovine albumin	Abcone	AA23088
Sheep Serum	Meilunbio	M134510
Nitro Blue Tetrazolium	Sigma-Aldrich	N6639-1G
5-bromo-4-chloro-3-indolyl phosphate	Sigma-Aldrich	136149
Trizol reagent	CWBio	03917
Lipofectamine 2000 Transfection Reagent	Invitrogen	11668030
Cas9 protein (4000ng/ul)	Genscript	Cat. #Z03469
Retinoic acid	Abcone	R26255
DiSBAC2(3)	Invitrogen	B413
Vinpocetine	MedChemExpress	HY-13295
Dibucaine	MedChemExpress	HY-B0552
2-APB	ENZO	ALX-400-045-M100
STO-609	MedChemExpress	HY-19805
Verapamil	MedChemExpress	HU-A0064
KN-62	MedChemExpress	HY-13290
Mibefradil	MedChemExpress	HY-15553A
Forskolin	MedChemExpress	HY-15371
SNAP	Tocris	1561
FBS	Gibco	1009914
penicillin streptomycin	Gibco	15140122
Xba1 restriction enzyme	Anza thermo scientific	#ER0682
NovoRec plus One-step PCR Cloning Kit	Novoprotein	NR005-01B
Protease Inhibitor Mini Tablets, EDTA free	Thermo/Pierce	88666
DMSO	MDBio	D015-100ml
Ethanol	Guo Yao Hu Shi	10009228
Methanol	Guo Yao Hu Shi	10014128
Thyroid hormone	MedChemExpress	HY-A0070A
Skim milk	Yi Li (伊利)	1142309
PTU (1-phenyl 2-thiourea)	Sigma	P7629
**Commerical assays**		
qRT-PCR reagent	Thermofisher	
ECL Chemiluminescence	Perkin Elmer	NEL105001EA
5x HiScript III qRT SuperMix	Vazyme	L/N 7E350C9
2x ChamQ Universal SYBR qRT-PCR Master Mix	Vazyme	L/N TE342F9
T7 MESSAGE MACHINE	Invitrogen	#AM1344
BCA method kit	MDbio	KT054-200rxn
T7 high-yield RNA transcription kit	Vazyme	#TR101-01
**Experimental models: Cell lines**		
HEK293	ATCC Cell Products	www.atcc.org
**Experimental models: animal lines**		
Tg[*hsp70*:GFP]	generated in lab	
Tg[hsp70:kcnk10a-GFP]	generated in lab	
Tg[*hsp70*:*kcnk5b*-GFP]	generated in lab	
Tg[*hsp70*:*rcan2*-mCherry]	generated in lab	
Tg[*hsp70*:Kirin1]	generated in lab	
Tg[*hsp70*: *kcnk5b*-mCherry]	generated in lab	
Tg[Cca.actb:GCaMP6s]	Zebrafish Stock Center Wuhan, China	CZ1282 doi.org/10.3390/ijms22115551
**Oligonucleotides**		
primer sequences	company purchase	
MGSO-tgcaccatctgtcactctgttaacctc	https://www.sangon.com/	
GPO-3-gggagcaaacaggattagataccct	https://www.sangon.com/	
CRISPR knockout *rcan2* for sequencing F: caacacactctctggctttcag	https://www.sangon.com/	
CRISPR knockout *rcan2* for sequencing R: tgaactgcatagttgagatggg	https://www.sangon.com/	
Rcan2 antisense probe F: taatacgactcactatagggctgggccaagcttggcaac	https://www.sangon.com/	
Rcan2 antisense probe R: gaaatgtttgaagcgctcttcc	https://www.sangon.com/	
CRISPR knockout *kcnk5b* for sequencing F: atcaccagaaacttgggagtgt	https://www.sangon.com/	
CRISPR knockout *kcnk5b* for sequencing R: gcttttgcgtgagaactaccat	https://www.sangon.com/	
In situ *kcnk5b* F:atggcagataaaggacctattttgac	https://www.sangon.com/	
In situ *kcnk5b* R: taatacgactcactataggccctcaaccaaagcttttcctcttccc	https://www.sangon.com/	
In situ *shha* F:tgcggcttttgacgagagtgc	https://www.sangon.com/	
In situ *shha* R: ggtaatacgactcactatagggtttcccgcgctgtctgccg	https://www.sangon.com/	
In situ *fgf8a* F:atgagactcataccttcacg	https://www.sangon.com/	
In situ *fgf8a* R: ggtaatacgactcactatagggtttgagaaagtctctggct	https://www.sangon.com/	
In situ *fgf10a* F:atgtgcaaatggaaagtgac	https://www.sangon.com/	
In situ *fgf10a* R: ggtaatacgactcactataggtctttcctcagtgcagttaa	https://www.sangon.com/	
In situ *aldh1a2* F:gggaaaaaaacccaaccg	https://www.sangon.com/	
In situ *aldh1a2* R: ggtaatacgactcactataggttaccactttaccccaccaa	https://www.sangon.com/	
In situ *bmp4* F:atgattcctggtaatcgaatgctg	https://www.sangon.com/	
In situ *bmp4* R: ggtaatacgactcactatagggtaagagtctccgtttagcggcagc	https://www.sangon.com/	
In situ *msx2b* F:agttgactgccaaacctgctctgc	https://www.sangon.com/	
In situ *msx2b* R: ggtaatacgactcactataggcatgactacaggtatgtcaac	https://www.sangon.com/	
qRT-PCR zebrafish *shha* F:ccactacgagggaagagctg	https://www.sangon.com/	
qRT-PCR zebrafish *shha* R:gagcaatgaatgtgggcttt	https://www.sangon.com/	
qRT-PCR zebrafish *aldh1a2* F: aaccactgaacacggacctc	https://www.sangon.com/	
qRT-PCR zebrafish *aldh1a2* R:ctccagtttggctccttcag	https://www.sangon.com/	
qRT-PCR zebrafish *fgf8a* F:catatgttgcgagtcacagtgtgga	https://www.sangon.com/	
qRT-PCR zebrafish *fgf8a* R: tggttacctgagcatagtagcaaaacg	https://www.sangon.com/	
qRT-PCR zebrafish *fgf10a* F:ctacggggcgagggatttc	https://www.sangon.com/	
qRT-PCR zebrafish *fgf10a* R:ggcttctcagtcttgcacct	https://www.sangon.com/	
qRT-PCR zebrafish *ef1a* F:ctggaggccagctcaaacat	https://www.sangon.com/	
qRT-PCR zebrafish *ef1a* R: atcaagaagagtagtaccgctagcattac	https://www.sangon.com/	
qRT-PCR zebrafish *cyp26a1* F: gccagcatcagtgagaaccat	https://www.sangon.com/	
qRT-PCR zebrafish *cyp26a1* R: cctgctggatcacgggaatgta	https://www.sangon.com/	
qRT-PCR human SHH F:ccgagcgatttaaggaactcacc	https://www.sangon.com/	
qRT-PCR human SHH R:agcgttcaacttgtccttacacc	https://www.sangon.com/	
qRT-PCR human GAPDH F: gtctcctctgacttcaacagcg	https://www.sangon.com/	
qRT-PCR human GAPDH R: gtctcctctgacttcaacagcg	https://www.sangon.com/	
qRT-PCR zebrafish *bmp4* F:cgcagccctaaacaaagag	https://www.sangon.com/	
qRT-PCR zebrafish *bmp4* R: tgattggtggagttgagatgat	https://www.sangon.com/	
qRT-PCR zebrafish *msx2b* F: gactgagatcagggattcttgc	https://www.sangon.com/	
qRT-PCR zebrafish *msx2b* R: gtgaggttgtgaatggtgtacg	https://www.sangon.com/	
qRT-PCR zebrafish *kcnk5b* F: atcactctcctcgtctgcaacg	https://www.sangon.com/	
qRT-PCR zebrafish *kcnk5b* R: gagtcccatgcacaacgtgcag	https://www.sangon.com/	
qRT-PCR zebrafish *rcan2-1* F: tgcagttgccaagcttggccca	https://www.sangon.com/	
qRT-PCR zebrafish *rcan2-1* R: tgtgagactggcaggtctgg	https://www.sangon.com/	
qRT-PCR zebrafish *rcan2-2* F: agagcttcagacgtgtacgaat	https://www.sangon.com/	
qRT-PCR zebrafish *rcan2-2* R: ctgttgccaaccgactgga	https://www.sangon.com/	
qRT-PCR zebrafish βactin2 F:gcagaaggagatcacatccctggc	https://www.sangon.com/	
qRT-PCR zebrafish βactin2 R: cattgccgtcaccttcaccgttc	https://www.sangon.com/	
sgRNAs and rescue mRNAs		
*rcan2* [in exon 4] aaccgactggaggtgagcca	IDT	
*rcan2* mRNA for *rcan2* KO rescue: atccaaccggtgggcttccg	IDT	
*kcnk5b* target2[in exon 1] tgcgatattccaaatcctcgagg	IDT	
*kcnk5b* target14 [in exon 1] ggtcttgggcgaaattattgagg	IDT	
*kcnk5b* mRNA for *kcnk5b* KO rescue-target2 tgcaatctttcagatactagaag	IDT	
*kcnk5b* mRNA for *kcnk5b* KO rescue-target14 ggtattaggagagatcatcgaag	IDT	
CRISPR scaffold gttttagagctagaaatagcaagttaaaataaggctagtccgttatcaacttgaaaaagtggcaccgagtcggtgct	https://www.sangon.com/	
**Software**		
ChopChop	(http://chopchop.cbu.uib.no/)/Computational Biol.Unit, Dept. of Informatics, Uni. of Bergen	version 3/10.1093/nar/gkz365
image J	NIH	FIJI
SymPhoTime64 ver 2.4	PicoQuant	RRID: SCR_016263
ZEN 3.4 (blue edition)	Zeiss	version 3.4
Prism9	Graphpad.com	version 9.5.1
**Other**		
2-photon-confocal Hyperscope	Scientifica	N/A
PMT-hybrid 40 MOD 5 photon detectors	PicoQuant	N/A
2-photon Chameleon Ultra II Ti Sapphire laser	Scientifica	N/A
Compound microscope VS120-S6-W	OLYMPUS	N/A
QuantStudio3 machine	Thermofisher	N/A
incubators	Thermofisher	FORMA STERI-CYCLE i160
Zeiss LSM 980	Zeiss	N/A
Amersha Imager 600 Chemiluminescence imaging system	General Electric	AI600

### qRT-PCR

For transgenic expression in fish embryos, the embryos were heat-shocked twice at 6-h intervals at 32 hpf and 42 hpf to maximize chronic transgene effects on developmental gene expression. Fin buds were isolated by using tip of a micropipette to gently remove the yolk of the 48 hpf embryos. The heads were removed using the small lancet iris knife (Zhongyuan Healthcare Equipment, Cat # hyl10.31372714). The main body of the embryo was removed by using tweezers to fix the body (neural tube facing up) and cutting the fin buds and residual yolk membrane from the body on both side with a small lancet iris knife. The fin buds and residual yolk membrane were collected in the TRIZOL (CWBio, 03917). Over 40 embryos (80 fin buds) were collected for each sample group. For adult caudal fin tissues, we heat shocked adult fish once for 12 min at 38.5°C 6 h before the tissue isolation. After anesthetizing adult fish with 0.4% Tricane, we surgically isolated the distal caudal fin tissues from the fin tip including the distalmost bone bifurcation. For qRT-PCR experiments from HEK293 cells, we isolated RNA from 90% confluent 6 cm well per replicate. The mRNA from fish or cultured HEK293 cells was isolated using Trizol (CWBio, 03917). Then, 1 μg mRNA was used for the reverse transcription to cDNA using 4× gDNA wiper Mix, 5× HiScript III qRT SuperMix (Vazyme, L/N 7E350C9). qRT-PCR was performed using 2× ChamQ Universal SYBR qPCR Master Mix (Vazyme, L/N TE342F9) with QuantStudio3 machine (Thermo Fisher). The cycle procedure was at 50.0°C for 2 min, 95°C for 10 min in the stage 1; 95°C for 15 s, 55.0°C for 20 s for 40 routine in the stage 2; 95°C for 15 s, 60°C for 1 min, 95°C for 15 s in the Melt Curve. Samples were standardized using the detected *ef1a* expression for fish mRNA isolation and GAPDH for human cell line mRNA isolations. Please see the “Table 1” in this section for the primer sequences of each gene.

### Protein extraction and western blot

Zebrafish were anesthetized with 0.4% Tricaine and fin tips (fin tissues from the distalmost edge of each fin to the 2 distalmost bone segments of the bone rays) were surgically isolated and homogenized in RIPA buffer with protease inhibitors (Thermo/Pierce), then incubated at 4°C for 1 h under constant shaking. Lysates were centrifuge at 13,000r for 15 min at 4°C. Protein concentration was determined using the BCA method kit (MDbio). Proteins were denatured (incubated at 95°C for 10 min in loading buffer and then transferred to ice immediately prior to loading (50 μg/lane) in a 10% SDS-PAGE Acrylamide-Bis (29:1) gel). After electrophoresis, gel contents were transferred onto 0.2 μm PVDF membrane. After transfer, the membrane incubated in 5% skim milk/0.5% TBST for 1.5 h at room temperature and then washed 3× 5 min room temperature. Blots were then incubated with Rcan2 primary antibody (1:1,000) overnight at 4°C. Blots were then wash in 1xTBST 3× 5 min and then incubated in secondary antibody (1:1,000) in blocking buffer for 2 h at room temperature. Blots were then washed 3× 5 min. Protein bands were detected using ECL chemiluminescence detection reagent (Perkin Elmer) and luminescence was detected using an Amersham Imager 600 chemiluminescence imager (General Electric).

### Cell culture

HEK293 cell cultures (from ATCC.org) were incubated at 37°C, 5% CO2, 95% humidity in incubators (Thermo Fisher, FORMA STERI-CYCLE i160) in DMEM medium (Gibco,1199506) with 10% FBS (Gibco,1009914) and 1% penicillin streptomycin (Gibco, 15140122). The identity of the cell lines was not authenticated, and mycoplasma was not detected. Cells were split to 50% density and transfected with Lipofectamin (Invitrogen, 11668–019) 12 h later. Expression for the transfected constructs was evaluated by expression of fluorescent marker. All cultures tested negative for mycoplasma, which we tested by collecting 1 ml of DMEM cultured over 24 h with HEK293 cells, and centrifuge at 12,000 rpm for 1 min. Then, do the PCR as following primers: MGSO-TGCACCATCTGTCACTCTGTTAACCTC, GPO-3-GGGAGCAAACAGGATTAGATACCCT.

### FRET-FLIM detection and analysis

HEK293 cells were transfected with 1 μg of pcDNA-kcnk5b-GFP; pcDNA6-mT2-CUTie-YFP [40] or pcDNA-CFP-cGi500-YFP [41]. The transgenic fish [*hsp70*:KIRIN1] were heat shock at 37°C for 1 h for the sensor to induce peak transgene expression 6 h later. Embryos were incubated in 250 μm PTU (1-phenyl 2-thiourea, Sigma) to prevent pigmentation starting at 24 hpf. Ten minutes before imaging, embryos were anesthetized in 4 μm MESAB (or 200 nM blebbistatin for 30 min, where indicated) and then embedded in 1% agarose before imaging. For other imaging experiments, the time points were indicated in the text or figure legends. For FLIM measurements of the adult caudal fins, adult fish (approximately 6 months old) were anesthetized with 1% MESAB and then placed on wet plastic Petri dish and imaged for 5 min and then placed back into aquarium water to regain consciousness. Measurements were from 3 different locations in the mesenchyme of caudal fins: 1 at each of the 2 lobe tips and 1 in the distal midline between the 2 lobes. Each measurement is 1 data point. For the embryonic developmental time course of fluorescence lifetime imaging (FLIM-FRET) measurements, each embryo was imaged at 32 hpf, 35 hpf, 38 hpf, 42 hpf, 48 hpf, and 56 hpf. Fluorescence lifetime imaging measurements were made by photon counting the fluorescence emission of either CFP (KIRIN1, cAMP, cGMP) using a 2-photon-confocal Hyperscope (Scientifica, United Kingdom) and PMT-hybrid 40 MOD 5 photon detectors (Picoquant, Germany). We determined exposure times for each plane based on the number of photons detected individual pixels. In each fin bud, we generally measured 3 regions within the mesenchyme and ectoderm by selecting regions of interest at distal, proximal, and central locations within the buds. Each region contained several pixels of that ranged from 1 to 3 cells in size. For each measurement, we used thresholds of several hundred photons per pixel to overcome background photon emissions and prevent any influence in the FLIM measurements. The lifetime curves were analyzed from graphically selected regions or cells in the confocal plane. The lifetime curves were determined (curve fitting) from the decay of the counted photon emissions after single laser pulses (2-photon Chameleon Ultra II Ti Sapphire laser) using the algorithm

$$y(t)=\sum_{i=0}^{n-1}IRF⊗|bkgrIRF|ShiftIRF\;A[i](t/t[i])+\sum_{i=0}^{n-1}A[i]\exp((-t+Period-IRFCenter)/\tau [i])+BkgrDECISum=\sum_{k=0}^{n-1}I[k]$$

for base computations of multi-exponential reconvolution with corrections for instrument response time (instrument response factor, IRF) and removal of background signals <100/pixel in the program SymPhoTime 64, version 2.4 (Picoquant, Germany, RRID: SCR_016263). The fitted curves represented 2 component (fluorophores FRET at <10 nm and fluorophores no FRET >10 nm) equations with χ^2^ values of 1 ± 0.19 for accuracy. To visualize differences in spatial distribution of each pixel lifetime value, individual lifetime values were distributed along a rainbow scale in which boundary lifetime values were assigned: blue lowest boundary and red highest boundary. FRET efficiency (on which our FLIM measurements are based) using our donor-only (CFP) and sensor-based donor + acceptor (CFP-YFP) K^+^-detection data in Fig 1A. The resulting average efficiency was 37.9%. We entered this calculated efficiency in a standardized equation [R_DA_ = R_0_ x ^6^√(1-Efficiency)2/Efficiency)] to calculate the effective distance between the 2 fluorophores for FRET from our measurements, (4.59 Å) and then to compare this to the reference distance of maximal FRET of CFP and YFP (4.7 Å) [93]. R_DA_ is calculated distance between donor and acceptor fluorophores. R_0_ is the distance required for 50% efficiency. The closeness our calculated and reference distances indicated meaningful efficiency of the K^+^-sensor in our in vivo measurements.

### KIRIN1 sensor

As previously published, KIRIN1 sensor was generated by fusing CFP and YFP to the N- and C-termini of the K^+^-binding protein (Kbp) from *E*. *coli* [28]. The Kbp protein normally maintains an elongated conformation that keeps the donor and acceptor fluorophores apart, which prevents FRET between the fluorophores. When K^+^ binds to the bacterial OsmY (BON) domain in the N-terminus and the Lysin motif (LysM) in the C terminus, it brings the 2 fluorophores together to allow FRET [28]. This results in a reduction in the lifetime of the donor (CFP) fluorophore, which can be detected by FLIM.

### DiSBAC_2_(3) staining and measurements

DiSBAC_2_(3) was diluted to a 10 μm concentration in 1× E3 medium that contained 250 μm concentration of PTU (1-phenyl 2-thiourea, Sigma, P7629). Embryos were put into PTU at 24 hpf to prevent the pigmentation of the embryos. Three hours before imaging, embryos were incubated in diluted DiSBAC_2_(3) at RT under gentle rocking. They were immobilized by adding blebbistatin to a final concentration of 200 nM for 30 min before imaging, embedded in 1% low-melting agarose gel, and then imaged under a 2-photon-confocal Hyperscope (Scientifica, UK) at 900 nm to maximize the detection of the fluorescence from the DiSBAC_2_(3) dye and minimize fluorescence of mCherry under 2-photon stimulation (www.fpbase.org). Photon counts were detected using PMT-hybrid 40 MOD 5 photon detectors (Picoquant, Germany) that detect photons (provide a numerical count) for each pixel. We used blebbistatin, because it inhibits muscle movement by inhibiting myosin activity and does not target any ion channel. We imaged each embryo for 3 min and measured 6 pixels in specific regions in each fin bud (distal mesenchyme, anterior mesenchyme, proximal mesenchyme, and posterior mesenchyme). The 6 pixels from each region were averaged and each average represents a data point in the graph; 32 hpf were chosen for imaging the effect of *kcnk5b* overexpression, because of the low levels of depolarization at this stage (**Fig 5A–5C**).

### CRISPR knockouts for rcan2 and kcnk5b

The ChopChop online tool (http://chopchop.cbu.uib.no/) was used to design sgRNAs to limit predicted off-target sgRNA cutting. The fourth exon of *rcan2* was targeted by the sgRNA sequence is 5′-AACCGACTGGAGGTGAGCCA-3′ ordered from IDT with Alt-R gRNA modification. The first exon in *kcnk5b* was targeted by the sgRNA sequence 5′-TGCGATATTCCAAATCCTCG-3′ and 5′-GGTCTTGGGCGAAATTATTG-3′ ordered by IDT with Alt-R gRNA modification, and 1 μl sgRNAs (400 ng/μl) and Cas9 (500 ng/μl) protein (Genscript, Cat. #Z03469) were co-injected into single-cell zebrafish embryos. After imaging for experiments, each embryo was numbered and raised separately to 5 dpf. Genomic DNA was isolated from each 5 dpf larva individually. After genomic DNA extraction by alkaline lysis buffer, the genomic region surrounding the target sites was PCR amplified using primers forward: 5′-CAACACACTCTCTGGCTTTCAG-3′ and reverse: 5′-TGAACTGCATAGTTGAGATGGG-3′ for *rcan2* or forward: 5′-ATCACCAGAAACTTGGGAGTGT-3′ and reverse: 5′-GCTTTTGCGTGAGAACTACCAT-3′ for *kcnk5b* and set for sequencing (Genewiz.com.cn).

### In vivo mRNA overexpression experiments

Mutated *rcan2*-P2A-mcherry, *kcnk5b*-GFP and *kcnk5bS345A*-GFP were cloned into pXT7 vector (from Lin Haifan and Bao Baolong lab) and either linearized with Xba1 (Anza Thermo Scientific #ER0682) or amplified by PCR to make templates. For *rcan2*, the original target sequence 5′-AACCGACTGGAGGTGAGCCA-3′ was mutated to 5′-ATCCAACCGGTGGGCTTCCG-3′. For *kcnk5b*, the original target-2 sequence 5′-TGCGATATTCCAAATCCTCGAGG-3′ was mutated to 5′-TGCAATCTTTCAGATACTAGAAG-3′, and the original target-14 sequence 5′-GGTCTTGGGCGAAATTATTGAGG-3′ was mutated to 5′-GGTATTAGGAGAGATCATCGAAG-3′ to impair interaction between the sgRNA targeting sequences and the rescue mRNAs without changing coded amino acids. The linear template was transcribed to mRNA using T7 MESSAGE MACHINE (Invitrogen, #AM1344). Concentrations injected into embryos: 1 μl Cas9 protein, 1 μl sgRNA, 1 μl *rcan2*-P2A-mCherry mRNA/*kcnk5b*-P2A-GFP (700 ng/1 μl), mCherry mRNA (375 ng/1 μl), 1 μl empty sgRNA (T7-sgRNA scaffold, 2,000 ng/μl) or *kcnk5bS345*-GFP/*kcnk5bS345A*-GFP (200 ng/μl).

### Mosaic analyses in fin buds

Double mosaic embryos were created by injecting 120 μg/μl of *hsp70*:*kcnk5b*-mCherry and 400 ng/μl into Tg[*hsp70*:KIRIN1] separately into 1–4 cell AB embryos. Embryos only expressing *kcnk5b*-mCherry mosaically were injected with *hsp70*:*kcnk5b*-mCherry into the transgenic Tg[Cca.actb:GCaMP6s] line. Injected embryos and larva were raised in 1xE3 medium (5 mM NaCl, 0.17 mM KCl, 0.33 mM CaCl_2_, 0.33 mM MgSO_4_, 10%–5% Methylene Blue). The mosaic larvae were selected by screening for mCherry and CFP expression for *kcnk5* and KIRIN1 transgenes or mCherry and GFP expression for *kcnk5b* and GCaMP6s transgenes in the fin buds 4 to 6 h after heat-shock induction.

### Small molecule treatments

All inhibitors (except for Vinpocetine) were dissolved in DMSO: Retinoic acid (Abcone, R26255) 10 mM stock concentration, 2-APB (IP_3_R inhibitor, ENZO, ALX-400-045-M100) 75 mM stock concentration, STO-609 (CaMKK inhibitor, MedChemExpress, HY-19805) 0.5 mM stock concentration, Verapamil (T-/L-type Ca^2+^ channel inhibitor, MedChemExpress, HU-A0064) 100 mM stock concentration, KN-62 (CaMKII/IV inhibitor, MedChemExpress, HY-13290) 1 mM stock concentration, or in sterile water Mibefradil (T-/L-type Ca^2+^ channel inhibitor, MedChemExpress, HY-15553A) 2.5 mM stock concentration, Forskolin (adenylyl cyclase activator, MedChemExpress, HY-15371) 25 mM, SNAP (guanylyl cyclase activator, Tocris, 1561) 100 mM stock concentrations. HEK293 cells were incubated in DMEM cell culture medium (Gibco, 1199506) containing 10% FBS (Gibco, 1009914) and 1% penicillin streptomycin (Gibco, 15140122) at 37°C, 5% CO_2_. Ten hours after transfection, the drugs were added to the medium to their final concentrations (as indicated in the figure legends or the specific methods section). Cells were then trypsinized and RNA was isolated as indicated for qRT-PCR. For fish embryos, we added the small molecules mentioned above to E3 to the final concentrations (200 nM RA, 13 μm 2-APB, or 24 μm STO unless indicate otherwise) and incubated the embryos 28°C. We determined the treatment times and concentrations (provided in the figure legends or text) to be the most effective on the fin bud growth and have the least effect on embryonic development. Adult fish were treated with 100 μm RA for 6 h, since we determined this concentration showed reproducible effects (S5F Fig). Vinpocetine (MedChemExpress, HY-13295) was dissolved in ethanol to make a 10 mM stock, Dibucaine (MedChemExpress, HY-B0552) was dissolved in DMSO to make a 500 mM stock. For assessing the effects of these inhibitors on depolarization, we treated embryos with 10 μm Vinpocetine and 40 μm Dibucaine for 4 h before assessment, since these concentrations showed continued reproducible inhibitory effects. For fin bud growth experiments, 10 μm Vinpocetine and 40 μm Dibucaine treatment started at 36 hpf for 12 h to maximize the effects of the inhibitor on fin bud growth by 48 hpf.

### GCaMP6s measurements

HEK293 cells were transfected with 1 μg of CMV-GCaMP6s, 1 μg of pcDNA-kcnk5b-MCherrry or pcDNA-mCherry or pcDNA-Kcnk5bMut. The pcDNA-Kcnk5bMut harbors 2 mutations: one changing the LEEP sequence in the first transmembrane region to VKKA and substituting S345 in the C-terminal tail with alanine. This generated a dead channel. HEK293 cells for Fig 6E were transfected and sorted by SORP ARIA Fusion (BD Biosciences) for mCherry. GCaMP6s transgenic fish Tg[Cca.actb:GCaMP6s] [43] were purchased from Zebrafish Stock Center Wuhan, China (CZ1282 doi.org/10.3390/ijms22115551). One-celled embryos from the established Transgenic fish Tg[Cca.actb:GCaMP6s] [43] were injected with Tg[*hsp70*-*kcnk5b*-mCherry] and were heat shocked at 37°C for 1 h at 42 hpf and imaged at 48 hpf. All the samples were imaged by Zeiss LSM 980 upright and analyzed by ZEN-Blue (Zeiss). To standardize intensity measurements and limit influence of differences in expression, we standardized all measurements to GCaMP6s cells lacking mCherry expression in the spinal cord (body) away from the fin bud. The results are provided as GFP bud/GFP body.

### Patch clamping

Transfected HEK293T cells were seeded on glass coverslips (Fisher Brand) and incubated in cell culture medium at 37°C, 5% CO_2_, 95% relative humidity for 8 to 10 h. The seeded coverslips were transferred into Tyrode’s solution (138 mM NaCl, 4 mM KCl, 2 mM CaCl_2_, 1 mM MgCl_2_, 0.33 mM NaH_2_PO4, 10 mM Glucose, 10 mM HEPES). Cells were assessed in the ruptured-patch whole-cell configuration of the patch-clamp technique using and EPC9 or EPC10 amplifier (HEKA) with borosilicate glass pipettes (Sutter Instruments) with 3 to 6 MΩ resistance when filled with pipette solution (130 mM glutamic acid, 10 mM KCl, 4 mM MgCl_2_, 10 mM HEPES, 2 mM ATP, pH to 7.2). For detecting potassium current, after gigaseal formation, cells were voltage-clamped at −80 mV. Potassium conductance was elicited by test pulses from −100 mV to 70 mV (in 10 mV increments) of 600 ms duration at a cycle length of 10 s. The resulting tracings were converted into itx files by the ABF Software (ABF Software, RRID: SCR_019222) and then analyzed using Clampfit Software (Molecular Devices, RRID: SCR_011323). Currents were measured at the end of the test pulses.

### Quantification and statistical analyses

To select for accurate two-component decay curve fittings of the FRET sensors, we used Chi-square analyses limiting fits 0.8>χ2<1.2 values, which was calculated within the SymPhoTime 64 software, version 2.4 (Picoquant, Germany, RRID: SCR_016263). Graphs were assembled and statistical analyses were done using Microsoft Excel (Microsoft.com) or Prism9 (Graphpad.com). All statistical analyses for graphs were done using the Students *t* test to assess the reproducibility of any differences between 2 groups within the data sets of each experiment. *P* values differences of >0.05 were indicated to be not significant “NS”; otherwise, the calculated *P* values were provided in the figure.

## Supporting information

S1 Data Meta data to Fig 1. (XLSX)

S2 Data Meta data to Fig 2. (XLSX)

S3 Data Meta data to Fig 3. (XLSX)

S4 Data Meta data to Fig 4. (XLSX)

S5 Data Meta data to Fig 5. (XLSX)

S6 Data Meta data to Fig 6. (XLSX)

S7 Data Meta data to Fig 7. (XLSX)

S8 Data Meta data to S1 Fig. (XLSX)

S9 Data Meta data to S2 Fig. (XLSX)

S10 Data Meta data to S3 Fig. (XLSX)

S11 Data Meta data to S4 Fig. (XLSX)

S12 Data Meta data to S5 Fig. (XLSX)

S13 Data Meta data to S8 Fig. (XLSX)

S14 Data Meta data to S9 Fig. (XLSX)

S1 Fig FLIM-FRET measurements with KIRIN1 sensor detects relative changes in intracellular K^+^. **(Aa)** Patch-clamp experiments show increased K^+^ currents from K^+^ leaking out of cells expressing CMV:*kcnk5b*-mCherry (blue) compared to cells expressing the control CMV:mCherry plasmid (red). **(Ab)** FLIM-FRET measurements of the KIRIN1 sensor expressed in the same cells that were patched in Aa detected decreases in intracellular K^+^ in cells expressing CMV:*kcnk5b*-mCherry (blue) compared to cells lacking the expression of the channel (CMV:mCherry, red). **(B)** FLIM-FRET measurements for intracellular K^+^ levels in HEK293 cells transfected with the CMV:KIRIN1 sensor and either CMV:mCherry or CMV:Kcnk5b-mCherry. Cells were either treated with DMSO or FK506. **(C)** FLIM-FRET measurements for intracellular K^+^ levels in HEK293 Cells transfected with the CMV:KIRIN1 sensor and either CMV:Kcnk5bS345A-mCherry, CMV:Kcnk5bWT-mCherry, or CMV:Kcnk5bS345E-mCherry. The measurements were converted to fold difference by dividing their lifetime measurements with the lifetime measurements of the control cells expressing the KIRIN1 sensor and mCherry. Each experiment was repeated at least 3 times (*N* = 3). For cell patch clamping and FLIM measurements, each data point represents 1 cell (A, B, C). For fish embryo FLIM imaging, we measured 2 or 3 locations in each tissue of 1 fin bud per embryo. *P* values represent statistical analysis by Student’s two-tailed *t* test. Numerical data used in this figure are included in S8 Data.
(TIF)

S2 Fig FLIM-FRET measurements not affected by changes in expression levels or sedation method. **(A)** Heat-shock method for inducing expression of the transgenic K^+^ sensor at the indicated time points for subsequent FLIM-FRET measurements. **(B)** Fluorescent image of non-transgenic sibling 6 h after heat shock at 48 hpf. **(C)** Fluorescent image of transgenic Tg[*hsp70*:KIRIN1] 6 h after heat shock at 48 hpf. **(D)** Representative confocal plane through a developing pectoral fin bud of a Tg[*hsp70*:KIRIN1] transgenic fish shows expression of the K^+^-sensor transgene in cells except for the nuclei. **(E)** Illustration of different decay (lifetime) curves and that despite differences in initial excitation levels of the donor fluorophore (arrows), the decay rates are similar (orange decay curves) unless energy is transferred from the donor to the acceptor fluorophore by FRET in the presence of K^+^, which will reduce the lifetime (blue decay curve). These specific differences in lifetime can be represented by specific colors along a rainbow scale to produce an image that relates the lifetime value of each pixel in the confocal plane to provide a spatial representation of the distribution of relative K^+^ levels in the fin bud. **(F)** 3D images of density map for illumination intensity (**a, b**) of pectoral fin buds at 32 hpf. Comparison between 2D plane of density map for illumination at a region of high intensity (**c**) and the lifetime assessment of the same region in the ectoderm (**d**). Comparison between 2D plane of density map for illumination at a region of low intensity (**e**) and the lifetime assessment of the same region in the ectoderm (**f**). Comparison between 2D plane of density map for illumination at a region of high intensity (**g**) and the lifetime assessment of the same region in the mesenchyme (**h**). Comparison between 2D plane of density map for illumination at a region of low intensity (**i**) and the lifetime assessment of the same region in the ectoderm (**j**). **(k)** Graph of lifetime values of each region measured (**d, f, h, j**) shows that high and low differences in intensity (y-axis) do not significantly alter the lifetime values (x-axis) of the ectoderm and the mesenchyme. **(G)** 3D images of density map for illumination intensity (**a, b**) of pectoral fin buds at 48 hpf. Comparison between 2D plane of density map for illumination at a region of high intensity (**c**) and the lifetime assessment of the same region in the ectoderm (**d**). Comparison between 2D plane of density map for illumination at a region of low intensity (**e**) and the lifetime assessment of the same region in the ectoderm (**f**). Comparison between 2D plane of density map for illumination at a region of high intensity (**g**) and the lifetime assessment of the same region in the mesenchyme (**h**). Comparison between 2D plane of density map for illumination at a region of low intensity (**i**) and the lifetime assessment of the same region in the ectoderm (**j**). **(k)** Graph of lifetime values of each region measured (**d, f, h, j**) shows that high and low differences in intensity (y-axis) do not significantly alter the lifetime values (x-axis) of the ectoderm and the mesenchyme. **(H)** 3D images of density map for illumination intensity (**a, b**) of pectoral fin buds at 56 hpf. Comparison between 2D plane of density map for illumination at a region of high intensity (**c**) and the lifetime assessment of the same region in the ectoderm (**d**). Comparison between 2D plane of density map for illumination at a region of low intensity (**e**) and the lifetime assessment of the same region in the ectoderm (**f**). Comparison between 2D plane of density map for illumination at a region of high intensity (**g**) and the lifetime assessment of the same region in the mesenchyme (**h**). Comparison between 2D plane of density map for illumination at a region of low intensity (**i**) and the lifetime assessment of the same region in the ectoderm (**j**). **(k)** Graph of lifetime values of each region measured (**d, f, h, j**) shows that high and low differences in intensity (y-axis) do not significantly alter the lifetime values (x-axis) of the ectoderm and the mesenchyme. **(I)** Representative FLIM-FRET image of the distribution of the lifetimes of a fin bud from a 32 hpf embryo immobilized with 200 nM Blebbistatin. **(J)** Representative FLIM-FRET image of the distribution of the lifetimes of a fin bud from a 32 hpf embryo immobilized with 4 μm MESAB (Tricane). **(K)** Representative FLIM-FRET image of the distribution of the lifetimes of a fin bud from a 48 hpf embryo immobilized with 200 nM Blebbistatin. **(L)** Representative FLIM-FRET image of the distribution of the lifetimes of a fin bud from a 48 hpf embryo immobilized with 4 μm MESAB. **(M)** Representative FLIM-FRET image of the distribution of the lifetimes of a fin bud from a 56 hpf embryo immobilized with 200 nM Blebbistatin. **(N)** Representative FLIM-FRET image of the distribution of the lifetimes of a fin bud from a 56 hpf embryo immobilized with 4 μm MESAB. **(O)** Graphed FLIM-FRET measurements of several fin buds of embryos immobilized either with MESAB or Blebbistatin from the indicated time points shows no significant differences (*P* > 0.05) between the 2 treatments. For the FLIM measurements of Blebbistatin-treated and MESAB-treated embryos, experiments were repeated at least 3 times (*N* = 3), and each repeat contained 3 or more embryos. We measured 2 or 3 locations in each tissue of 1 fin bud per embryo. Each measured value is represented as a data point (O). *P* values represent statistical analysis by Student’s two-tailed *t* test. *P* values ≥0.05 are designated as “not significant” (NS). Scale bars equal 1 mm (B, C), 20 μm (D), 30 um (Ha-i), or as indicated in the panels. Numerical data used in this figure are included in S9 Data.
(TIF)

S3 Fig Confocal planes of FLIM-FRET images in fin buds at different developmental time points. **(A)** Confocal planes in fin bud from a 32 hpf embryo. The distance between the first plane and last plane is 15.2 μm. **(B)** Confocal planes in a fin bud from a 48 hpf embryo. The distance between the first plane and last plane is 18.36 μm. **(C)** Confocal planes in a fin bud from a 56 hpf embryo. The distance between the first plane and last plane is 10.3 μm. Numbers in lower right of each panel indicate the order of the indicated confocal plane through the Z-stack. **(D)** FLIM measurements in fin buds of 56 hpf embryos of indicated cell categories from the transgenic KIRIN1 fish line Tg[*hsp70*:KIRIN1] mosaically expressing mCherry or *kcnk5b*-mCherry. “Adj” indicates cells adjacent to mCherry-positive (mCherry+) or *kcnk5b*-mCherry-positive (*kcnk5b*+) cells. Each experiment was repeated at least 3 times (*N* = 3). For fish embryo FLIM imaging, we measured 2 or 3 locations in each tissue of 1 fin bud per embryo. Each measured value is represented as a data point (D). Numerical data used in this figure are included in S10 Data.
(TIF)

S4 Fig Overexpression of K^+^-leak channels and their effects on pectoral fin bud growth. **(A)** qRT-PCR for *kcnk5b*-GFP expression after single heat shock pulse of non-transgenic siblings and Tg[*hsp70*:*kcnk5b*-GFP] siblings. Each RNA sample was isolated from fin buds of 40+ embryos at 48 hpf. **(B)** Expression of *kcnk5b*-GFP in the body (**a**) and in the fin bud (**b**) of a representative Tg[*hsp70*:*kcnk5b*-GFP] 56 hpf embryo 6 h after heat shock. **(C)** Expression of *kcnk10a*-GFP in the body (**a**) and in the fin bud (**b**) of a representative Tg[*hsp70*:*kcnk10a*-GFP] 56 hpf embryo 6 h after heat shock. **(D)** Brightfield image of thorax region of a post-heat-shocked 48 hpf non-transgenic embryo. The area of the fin bud (highlighted by a black-dotted line). The otic vesicle was used as a size standard (highlighted by a red-dotted line). **(E)** Brightfield image of thorax region of a post-heat-shocked 48 hpf *hsp70*:*kcnk5b*-GFP transgenic embryo with the fin bud (black-dotted line) and otic vesicle (red-dotted line) used as a size standard. **(F)** Measurements of pectoral fin bud areas of heat-shocked groups of non-transgenic (Non-Tg) and Tg[*hsp70*:GFP] (GFP-Tg) as controls and Tg[*hsp70*:*kcnk5b*-GFP] transgenic fish line as well as the Tg[*hsp70*:*kcnk10a*-GFP] transgenic fish line. Each measured bud area was standardized to the otic vesicle area in the same embryo and each measurement is represented as a ratio of bud-area–to–otic-vesicle area. Each experiment was repeated at least 3 times (*N* = 3) and each repeat contained 3 for more fish. Each data point represents 1 fin bud measurement per embryo. *P* values represent statistical analysis by Student’s two-tailed *t* test. *P* values ≥0.05 are designated as “not significant” (NS). Numerical data used in this figure are included in S11 Data.
(TIF)

S5 Fig Effects of nuclear hormone treatments on *kcnk5b*, *rcan2*, and control genes in pectoral fin buds and adult fins and sufficiency/necessity experiments for *rcan2*. **(A)** qRT-PCR measurements of the transcription of *kcnk5b* in the developing pectoral fin bud with and without retinoic acid stimulation. **(B)** qRT-PCR measurements of *cyp26a* expression in pectoral fin buds with or without 200 nM retinoic acid treatment, a gene known to be induced by retinoic acid treatment. **(C)** FLIM measurements from Ectoderm (Ecto) and Mesenchyme (Mesen) cells of buds at 32 hpf treated either with DMSO or 200 nM thyroid hormone for 6 h. **(D)** In situ staining intensity measurements from the in situs of *rcan2* expression in the embryonic fin buds after DSMO or RA treatment. **(E)** qRT-PCR for expression of *rcan2* and *dio3b* in adult caudal fin after treatment with DMSO or 500 nM thyroid hormone for 24 h. *dio3b* is a gene known to be up-regulated by thyroid hormone stimulation in the adult caudal fin. **(F)** RT-PCR for expression of *cyp26a* treated either with DMSO or 100 nM RA for 6 h in adult caudal fin. **(G)** FLIM measurements of intracellular K^+^ levels in adult caudal fin cells after 6 h treated either with DMSO or 100 nM RA. **(H)** qRT-PCR measurements using 2 different primer sets for *rcan2* in adult caudal fins of indicated treatment groups. **(I)** Whole-mount in situ hybridization of a caudal fin 3 day post amputation shows *rcan2* expression in the distal blastemal but absent from the proximal blastema (a). Arrowheads indicate amputation plane. Cryo cross sections through the 3 day post amputation, distal tip of a regenerating adult caudal fin in the ray (b) and interray tissues (c) after in situ hybridization for *rcan2*. The blue color indicates *rcan2* expression. **(J)** Representative western blot for Rcan2 and beta-actin proteins from lysates of regenerating adult caudal fins at the indicated days post amputation (dpa). **(K)** Measurements of Rcan2 protein expression after standardization to beta-actin expression at the indicated time points. **(L)** Representative brightfield image of non-transgenic sibling 6 h after heat-shock stimulation. **(M)** Representative mCherry fluorescence image of non-transgenic sibling 6 h after heat-shock stimulation. **(N)** Representative brightfield image of transgenic Tg[*hsp70*:*rcan2*-mCherry] sibling 6 h after heat-shock stimulation. **(O)** Representative mCherry fluorescence image of transgenic Tg[*hsp70*:*rcan2*-mCherry] sibling 6 h after heat-shock stimulation. **(P–R)** Representative amputated caudal fin of heat-shocked AB non-transgenic (non-Tg) fish at time 0 days post amputation (dpa) (P) and regenerated 56 dpa (Q) together with its representative fluorescence image for mCherry (R). **(S–U)** Representative caudal fins of Tg[*hsp70*:*rcan2*-mCherry] transgenic fish (*rcan2*-Tg) at time 0 dpa (S) and regenerated 56 dpa (T) together with its representative fluorescence image for mCherry (U). **(V)** Fin-to-body length ratios of non-transgenic and Tg[*hsp70*:*rcan2*-mCherry] fish at the indicated time points during one-heat-shock per day regimen. **(W)** Wild-type 48 h post fertilization (hpf) larva (a). Sequence of *rcan2* gene in exon 4. (b) The sgRNA target site is indicated by the orange box. Of the total number of injected embryos (45) assessed, only 9% showed wild-type sequence. **(X)** 48 hpf larva of large deletion group (a). The majority of targeted embryos (64%) harbored a similar large deletion: indicated by the gap in sequence. The downstream sequence also showed scrambled nucleotide order (b). **(Y)** 48 hpf larva of small deletion group (a). Over a quarter of the targeted embryos (27%) harbored similar small deletions as well as scrambled downstream sequence (b). **(Z)** Representative mCherry fluorescent image of CRISPR rescue expression of rcan2*-mCherry mRNA (a) and CRISPR-targeted *rcan2* allele from an rcan2* mRNA-mCherry-expressing embryo (b). rcan2*-mCherry mRNA has mutated wobble-position nucleotides of codons to impair interaction with the sgRNA to continue sgRNA-mediated disruption of the *rcan2* alleles while maintaining the integrity of the transgenic Rcan2 protein. **(AA)** Representative images of control (a) and CRISPR-targeted *rcan2* KO (b) juvenile fish 76 days post fertilization. **(BB)** Caudal fin-to-body length ratios between control AB and *rcan2* KO juvenile fish. Scale bars equal 20 μm (I), 100 μm (L–NO, W–Z), 1 mm (P–U) and 500 μm (AA). For qRT-PCR experiments from fin buds, each experiment was repeated at least 3 time (*N* = 3) and each repeat contained duplicate or triplicate samples. Each sample contained 80+ fin bud isolations (A, B). For the FLIM measurements of embryos, we made 3 measurements per tissue in each fin bud. At least 3 embryos were measured per group per experiment, and each experiment was repeated 3 times. Each data point represents a measurement (C). For FLIM measurements from adult fins, we measured 3 locations in the mesenchyme of each unamputated adult caudal fin: one at each of the 2 lobe tips, and one in the midline between the 2 lobes at the midline of the fin. Each data point represents 1 measurement (G). For qRT-PCR and western blots experiments with adult caudal fins, each point represents a separate sample group from an isolation of the distal tips with 2 segments from 30+ fish (H, I). For fin bud and adult fin growth measurements, each experiment was repeated 3 times. The adult fin measurements in S5V contained 10+ fish per group each time. The measurements in S5BB, each point represents 1 fish. *P* values represent statistical analysis by Student’s two-tailed *t* test. *P* values ≥0.05 are designated as “not significant” (NS). Numerical data used in this figure are included in S12 Data.
(TIF)

S6 Fig CRISPR *kcnk5b* KO strategy, targeting efficiency of endogenous locus and Kcnk5b mutants. **(A)** Wild-type 48 hours post fertilization (hpf) larva (**a**), and sequence of *kcnk5b* gene in exon 1 (**b**). Of 100 embryos targeted, 12% do not show gene defects. **(B)** 48 hpf embryo (**a**) harboring a large deletion in the *kcnk5b* exon 1 (**b**), and 44% harbor large deletions. **(C)** 48 hpf embryo (**a**) harboring small deletions and sequence changes (**b**), and 44% harbor such small deletions. The sgRNA target sites are indicated by the gray box labeled “target.” **(D, E)** Representative embryo with *kcnk5b* CRISPR knockout (*kcnk5b* KO) brightfield (D) and lack of green fluorescence (E) of CRISPR targeted embryos. **(F, G)** Representative embryo *kcnk5b* KO brightfield (F) and GFP from control GFP mRNA. **(H, I)** Representative embryo *kcnk5b* KO rescued with expression of kcnk5b*-GFP mRNA, brightfield (H) and green fluorescence (I). kcnk5b*-GFP mRNA has mutated wobble-position nucleotides of codons to impair interaction with the sgRNA to continue sgRNA-mediated disruption of the *kcnk5b* alleles while maintaining the integrity of the transgenic Kcnk5b protein. **(J)** Representative disrupted *kcnk5b* allele sequence after CRISPR-targeting and kcnk5b*-GFP-expressing embryo. **(K, L)** Wild-type, uninjected embryo for GFP (K) and absence of mCherry (L) fluorescence. **(M, N)** Representative transgenic embryo for *kcnk5bS345*-GFP (M) and *rcan2*-mCherry (N) mRNAs. **(O, P)** Representative transgenic embryo for *kcnk5bS345A*-GFP mutant (O) and *rcan2*-mCherry (P) mRNAs. Scale bars equal 100 μm (A–C, D–I, K–P).
(TIF)

S7 Fig Development of embryos incubated with Vinpocetine, Dibucane, and DiSBAC2(3). **(A)** Representative image of a 48 hpf AB non-transgenic fish after 1 heat shock at 32 hpf and treated only with the solvents ethanol (EtOH) and DSMO for 12 h starting at 36 hpf. **(B)** Representative image of 48 hpf AB non-transgenic fish after 1 heat shock at 32 hpf and treated with 10 μm Vinpocetine (Vin) and 40 μm Dibuciane (Dib) for 12 h starting at 36 hpf. **(C)** Representative image of a 48 hpf Tg[*hsp70*:*kcnk5b*-GFP] fish heat-shocked once at 32 hpf to induce expression of *kcnk5b*-GFP and treated for 12 h with the solvents EtOH and DMSO at 36 hpf. **(D)** Representative image of a 48 hpf Tg[*hsp70*:*kcnk5b*-GFP] fish heat-shocked once at 32 hpf to induce expression of *kcnk5b*-GFP and treated with 10 μm Vinpocetine (Vin) and 40 μm Dibuciane (Dib) for 12 h starting at 36 hpf. **(E–J)** Embryos incubated in 10 μm DiSBAC_2_(3) dye for 3 h show dye penetration in the embryos. mCherry fluorescence of embryo treated with EtOH and DMSO before DiSBAC_2_(3) incubation (**E**). Brighfield image of embryo treated with EtOH and DMSO after DiSBAC_2_(3) incubation (**F**). Fluorescence of DiSBAC_2_(3) in embryo treated with 10 μm Vin and 40 μm Dib before DiSBAC incubation (**I**). mCherry fluorescence of embryo treated with Vin and Dib before DiSBAC_2_(3) incubation (**H**). Brighfield image of embryo treated with Vin and Dib after DiSBAC_2_(3) incubation (**I**). Fluorescence of DiSBAC_2_(3) in embryo treated with Vin and Dib after DiSBAC_2_(3) incubation (**J**).
(TIF)

S8 Fig Effects of drug treatments on culture cells and embryos. **(A)** FLIM-FRET measurements of intracellular cAMP levels. Forskolin used as positive control for cAMP production. **(B)** FLIM-FRET measurements of intracellular cGMP levels. SNAP used as positive control for cGMP production. **(C)** Patch-clamp experiment measuring the K+ leak from HEK293T cells transfected either with Kcnk5b-GFP (blue) or a mutated Kcnk5bMut-GFP (red) that displayed almost no channel activity. **(D**) Diagram for T- and L-type Ca^2+^ channel inhibition by verapamil or mibefradil. **(E)** qRT-PCR of SHH expression in HEK293 cells after treatment with verapamil at the indicated concentrations. **(F)** qRT-PCR of SHH expression in HEK293 cells after treatment with Mibefradil at the indicated concentrations. **(G)** Brightfield images of HEK293 cells transfected with CMV-mCherry and treated with DMSO (**a**) or transfected with CMV-Kcnk5b-mCherry and treated with DMSO (**b**) or with verapamil, a L-/T-channel inhibitor, at 50 μm (**c**), 100 μm (**d**), 140 μm (**e**), 200 μm (**f**). **(H)** Fluorescence of HEK293 cells transfected with CMV-mCherry and treated with DMSO (**a**), or transfected with CMV-Kcnk5b-mCherry and treated with DMSO (**b**) or with verapamil at 50 μm (**c**), 100 μm (**d**), 140 μm (**e**), 200 μm (**f**). **(I)** Brightfield images of HEK293 cells transfected with CMV-mCherry and treated with DMSO (**a**) or transfected with CMV-Kcnk5-mCherry and treated with DMSO (**b**) or with Mibefradil, a L-/T-channel inhibitor, at 3.5 μm (**c**), 5 μm (**d**). **(J)** Fluorescent images of HEK293 transfected with CMV-mCherry and treated with DMSO (**a**) or transfected with CMV-Kcnk5b-mCherry and treated with DMSO (**b**) or with Mibefradil at 3.5 μm **(c)** 5 μm (**d**). **(K)** Brightfield images of HEK293 cells transfected with CMV-GFP and treated with DMSO (**a**) or transfected with CMV-Kcnk5b-GFP and treated with DMSO (**b**) or with the IP_3_ receptor inhibitor 2-APB at 30 μm (**c**), 75 μm (**d**), 105 μm (**e**), 150 μm (**f**). **(L)** Fluorescence images of HEK293 cells transfected with CMV-GFP and treated with DMSO **(a)** or transfected with CMV-Kcnk5b-GFP and treated with DMSO (**b**) or the IP_3_ receptor inhibitor 2-APB at at 30 μm (**c**), 75 μm (**d**), 105 μm (**e**), 150 μm (**f**). Each experiment was repeated at least 3 times (*N* = 3) and each repeat contained duplicate or triplicate samples. *P* values represent statistical analysis by Student’s two-tailed *t* test. *P* values ≥0.05 are designated as “not significant” (NS). Scale bars equal 20 μm (B–E, M, O) and 100 μm (F–K). Numerical data used in this figure are included in S13 Data.
(TIF)

S9 Fig Effects of drug treatments on culture cells and embryos. **(A)** qRT-PCR for SHH in HEK293 cells transfected either with GFP or Kcnk5b-GFP and treated with DMSO or the CaMKII,IV inhibitor KN-62 at the indicated concentrations. **(B)** Brightfield images of HEK293 cells transfected with CMV-mCherry and treated with DMSO (**a**) or transfected with CMV-Kcnk5b-mCherry and treated with DMSO (**b**) or treated with KN-62 an inhibitor for CaMKII and CaMKIV at 2 μm (**c**) and 3 μm (**d**). **(C)** Fluorescence images of HEK293 cells transfected with CMV-mCherry and treated with DMSO (**a**) or transfected with CMV-Kcnk5b-mCherry and treated with DMSO (**b**) or treated with KN-62 an inhibitor for CaMKII and CaMKIV at 2 μm (**c**) and 3 μm (**d**). **(D)** Brightfield images of cells treated transfected with CMV-GFP and treated with DSMO (**a**) or transfected with CMV-Kcnk5b-GFP and treated with DSMO (**b**) or STO-609 an inhibitor of CaMKK at 0.2 μm (**c**), 0.5 μm (**d**), 1 μm (**e**), 1.5 μm (**f**). **(E)** Fluorescent images of cells treated transfected with CMV-GFP and treated with DSMO (**a**) or transfected with CMV-Kcnk5b-GFP and treated with DSMO (**b**) or STO-609 an inhibitor of CaMKK at 0.2 μm (**c**), 0.5 μm (**d**), 1 μm (**e**), 1.5 μm (**f**). **(F)** Representative 48 hpf *kcnk5b* transgenic Tg[*hsp70*:*kcnk5b*-GFP] embryo 12 h post heat shock and 6 h treatment in solvent concentration of DMSO. **(G)** Enlarge view of *kcnk5b*-Tg embryo (F) shows some heart edema associated with heat-shock-induced expression of *kcnk5b*. **(H)** Representative 48 hpf *kcnk5b* transgenic embryo 12 h post heat shock and 4 h treatment in the IP_3_R inhibitor 13 μm 2-APB. **(I)** Enlarge view of *kcnk5b* transgenic embryo in (H). **(J)** Representative 48 hpf *kcnk5b* transgenic embryo 12 h post heat shock and 6 h treatment in 24 μm STO. **(K)** Enlarge view of *kcnk5b* transgenic embryo in (J). **(L, M)** Cells transfected with CMV-*camkk1b*-mCherry in representative brightfield (**L**) and fluorescence (**M**) images. Each experiment was repeated at least 3 times (*N* ≥ 3) and each repeat contained duplicate or triplicate samples. *P* values represent statistical analysis by Student’s two-tailed *t* test. *P* values ≥0.05 are designated as “not significant” (NS). Scale bars equal 20 μm (D–I, K, M, N, P, Q, M, O) and 100 μm (R–W). Numerical data used in this figure are included in S14 Data.
(TIF)

## Abbreviations

AER: apical ectodermal ridge
ER: endoplasmic reticulum
FLIM: Fluorescence Lifetime Microscopy
FRET: Förster Resonance Energy Transfer
hpf: hours post fertilization
IRF: instrument response factor
RA: retinoic acid
TH: thyroid hormone
ZPA: zone of polarizing activity
