# Supplementary figures and images for: The scale of zebrafish pectoral fin buds is determined by intercellular K+ levels and consequent Ca2+-mediated signaling via retinoic acid regulation of Rcan2 and Kcnk5b
Source: PLoS Biol. 2024 Mar 25;22(3):e3002565. doi: 10.1371/journal.pbio.3002565 (PMC11018282; doi:10.1371/journal.pbio.3002565)

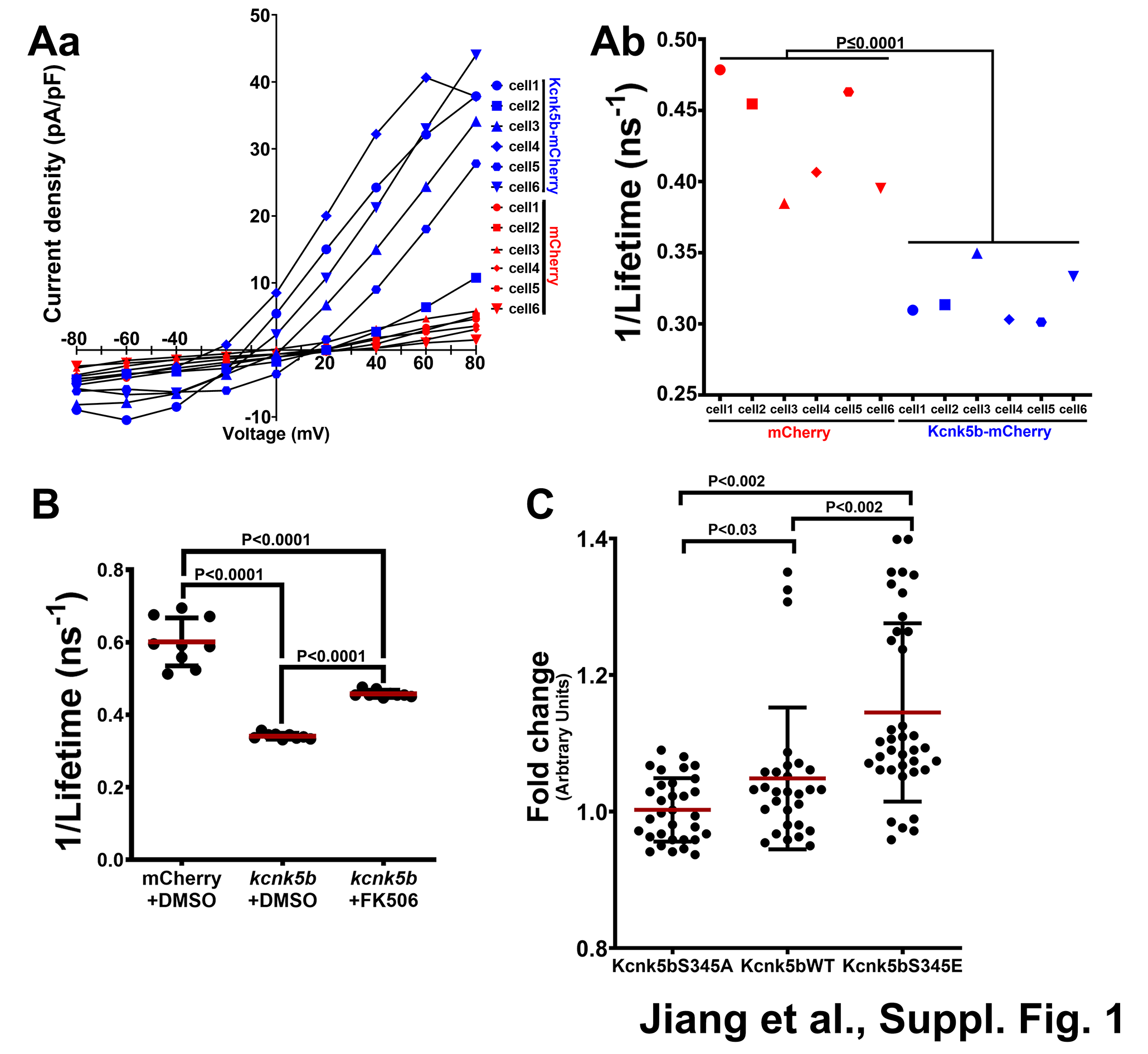

Supplement: S1 Fig — (Aa) Patch-clamp experiments show increased K+ currents from K+ leaking out of cells expressing CMV:kcnk5b-mCherry (blue) compared to cells expressing the control CMV:mCherry plasmid (red). (Ab) FLIM-FRET measurements of the KIRIN1 sensor expressed in the same cells that were patched in Aa detected decreases in intracellular K+ in cells expressing CMV:kcnk5b-mCherry (blue) compared to cells lacking the expression of the channel (CMV:mCherry, red). (B) FLIM-FRET measurements for intracellular K+ levels in HEK293 cells transfected with the CMV:KIRIN1 sensor and either CMV:mCherry or CMV:Kcnk5b-mCherry. Cells were either treated with DMSO or FK506. (C) FLIM-FRET measurements for intracellular K+ levels in HEK293 Cells transfected with the CMV:KIRIN1 sensor and either CMV:Kcnk5bS345A-mCherry, CMV:Kcnk5bWT-mCherry, or CMV:Kcnk5bS345E-mCherry. The measurements were converted to fold difference by dividing their lifetime measurements with the lifetime measurements of the control cells expressing the KIRIN1 sensor and mCherry. Each experiment was repeated at least 3 times (N = 3). For cell patch clamping and FLIM measurements, each data point represents 1 cell (A, B, C). For fish embryo FLIM imaging, we measured 2 or 3 locations in each tissue of 1 fin bud per embryo. P values represent statistical analysis by Student’s two-tailed t test. Numerical data used in this figure are included in S8 Data. (TIF) [file pbio.3002565.s015.tif]

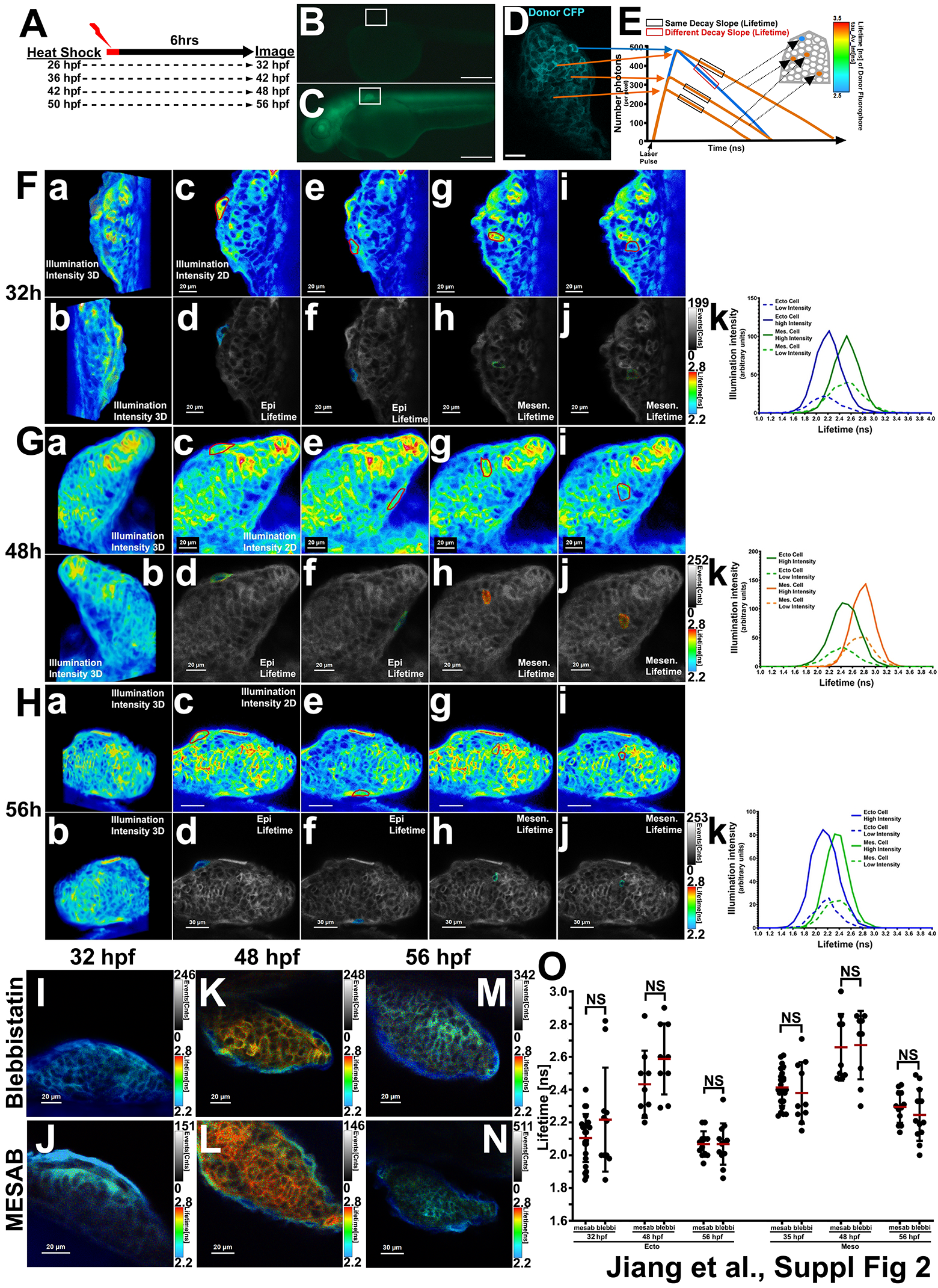

Supplement: S2 Fig — (A) Heat-shock method for inducing expression of the transgenic K+ sensor at the indicated time points for subsequent FLIM-FRET measurements. (B) Fluorescent image of non-transgenic sibling 6 h after heat shock at 48 hpf. (C) Fluorescent image of transgenic Tg[hsp70:KIRIN1] 6 h after heat shock at 48 hpf. (D) Representative confocal plane through a developing pectoral fin bud of a Tg[hsp70:KIRIN1] transgenic fish shows expression of the K+-sensor transgene in cells except for the nuclei. (E) Illustration of different decay (lifetime) curves and that despite differences in initial excitation levels of the donor fluorophore (arrows), the decay rates are similar (orange decay curves) unless energy is transferred from the donor to the acceptor fluorophore by FRET in the presence of K+, which will reduce the lifetime (blue decay curve). These specific differences in lifetime can be represented by specific colors along a rainbow scale to produce an image that relates the lifetime value of each pixel in the confocal plane to provide a spatial representation of the distribution of relative K+ levels in the fin bud. (F) 3D images of density map for illumination intensity (a, b) of pectoral fin buds at 32 hpf. Comparison between 2D plane of density map for illumination at a region of high intensity (c) and the lifetime assessment of the same region in the ectoderm (d). Comparison between 2D plane of density map for illumination at a region of low intensity (e) and the lifetime assessment of the same region in the ectoderm (f). Comparison between 2D plane of density map for illumination at a region of high intensity (g) and the lifetime assessment of the same region in the mesenchyme (h). Comparison between 2D plane of density map for illumination at a region of low intensity (i) and the lifetime assessment of the same region in the ectoderm (j). (k) Graph of lifetime values of each region measured (d, f, h, j) shows that high and low differences in intensity (y-axis) do not s [file pbio.3002565.s016.tif]

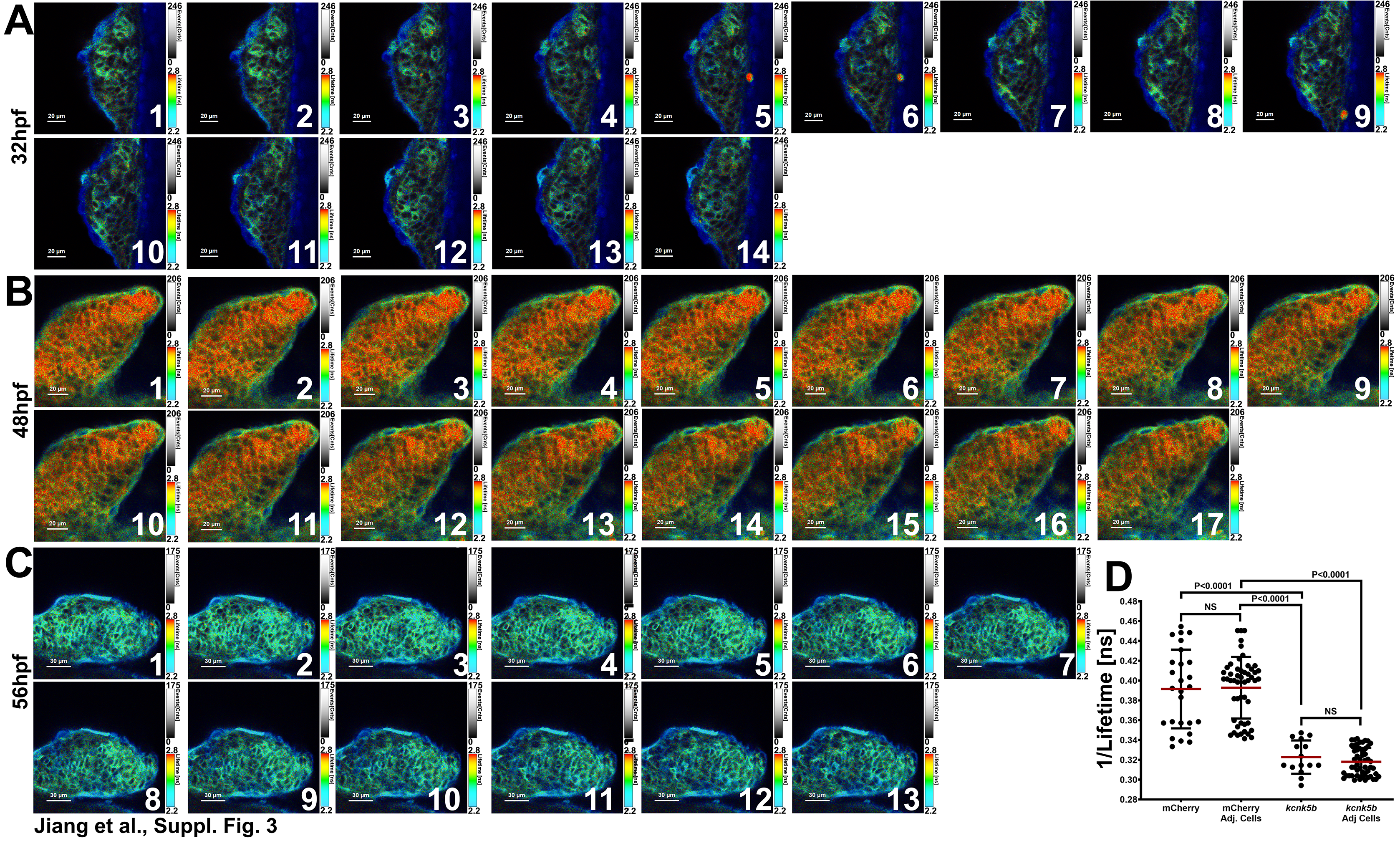

Supplement: S3 Fig — (A) Confocal planes in fin bud from a 32 hpf embryo. The distance between the first plane and last plane is 15.2 μm. (B) Confocal planes in a fin bud from a 48 hpf embryo. The distance between the first plane and last plane is 18.36 μm. (C) Confocal planes in a fin bud from a 56 hpf embryo. The distance between the first plane and last plane is 10.3 μm. Numbers in lower right of each panel indicate the order of the indicated confocal plane through the Z-stack. (D) FLIM measurements in fin buds of 56 hpf embryos of indicated cell categories from the transgenic KIRIN1 fish line Tg[hsp70:KIRIN1] mosaically expressing mCherry or kcnk5b-mCherry. “Adj” indicates cells adjacent to mCherry-positive (mCherry+) or kcnk5b-mCherry-positive (kcnk5b+) cells. Each experiment was repeated at least 3 times (N = 3). For fish embryo FLIM imaging, we measured 2 or 3 locations in each tissue of 1 fin bud per embryo. Each measured value is represented as a data point (D). Numerical data used in this figure are included in S10 Data. (TIF) [file pbio.3002565.s017.tif]

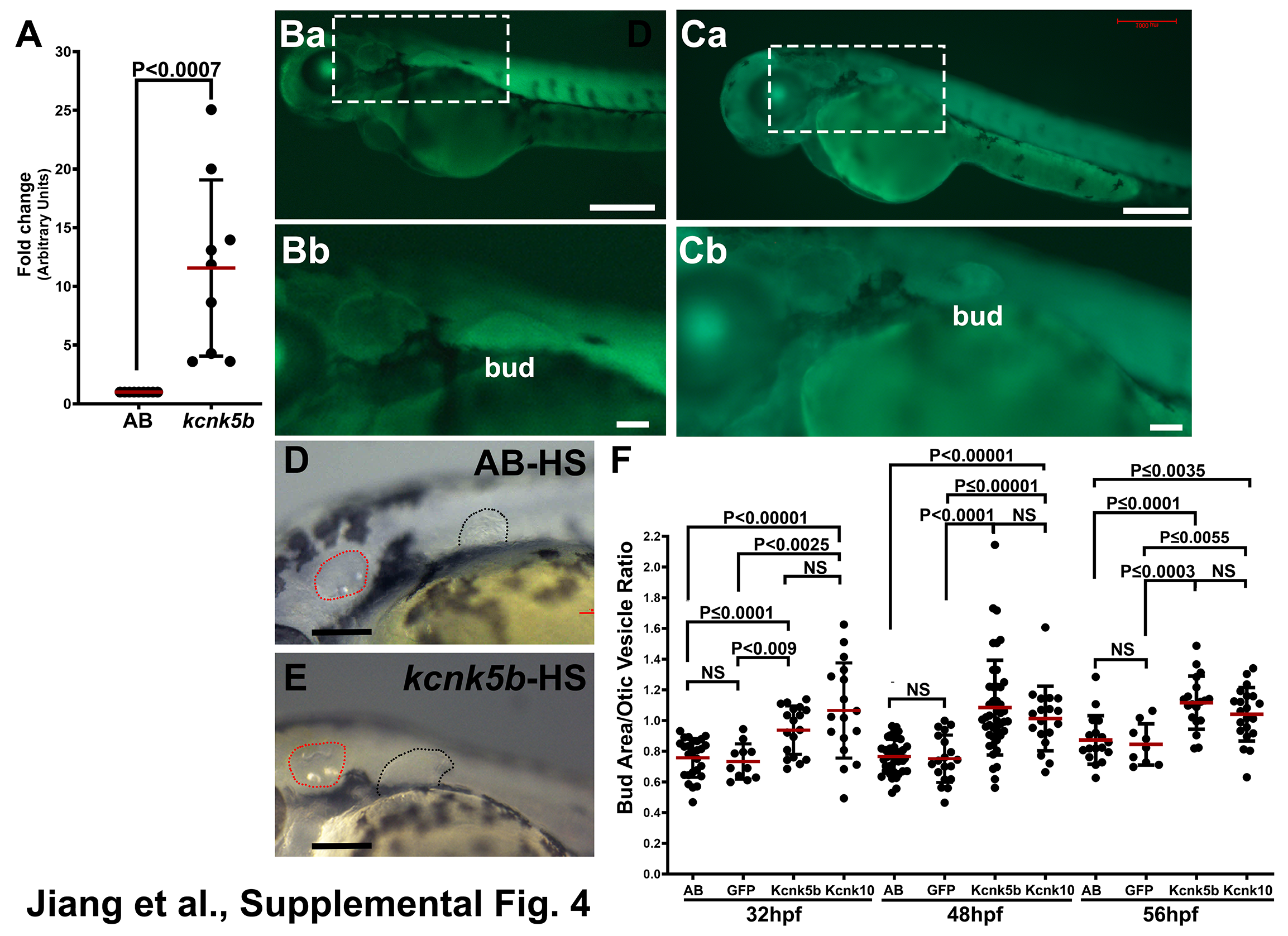

Supplement: S4 Fig — (A) qRT-PCR for kcnk5b-GFP expression after single heat shock pulse of non-transgenic siblings and Tg[hsp70:kcnk5b-GFP] siblings. Each RNA sample was isolated from fin buds of 40+ embryos at 48 hpf. (B) Expression of kcnk5b-GFP in the body (a) and in the fin bud (b) of a representative Tg[hsp70:kcnk5b-GFP] 56 hpf embryo 6 h after heat shock. (C) Expression of kcnk10a-GFP in the body (a) and in the fin bud (b) of a representative Tg[hsp70:kcnk10a-GFP] 56 hpf embryo 6 h after heat shock. (D) Brightfield image of thorax region of a post-heat-shocked 48 hpf non-transgenic embryo. The area of the fin bud (highlighted by a black-dotted line). The otic vesicle was used as a size standard (highlighted by a red-dotted line). (E) Brightfield image of thorax region of a post-heat-shocked 48 hpf hsp70:kcnk5b-GFP transgenic embryo with the fin bud (black-dotted line) and otic vesicle (red-dotted line) used as a size standard. (F) Measurements of pectoral fin bud areas of heat-shocked groups of non-transgenic (Non-Tg) and Tg[hsp70:GFP] (GFP-Tg) as controls and Tg[hsp70:kcnk5b-GFP] transgenic fish line as well as the Tg[hsp70:kcnk10a-GFP] transgenic fish line. Each measured bud area was standardized to the otic vesicle area in the same embryo and each measurement is represented as a ratio of bud-area–to–otic-vesicle area. Each experiment was repeated at least 3 times (N = 3) and each repeat contained 3 for more fish. Each data point represents 1 fin bud measurement per embryo. P values represent statistical analysis by Student’s two-tailed t test. P values ≥0.05 are designated as “not significant” (NS). Numerical data used in this figure are included in S11 Data. (TIF) [file pbio.3002565.s018.tif]

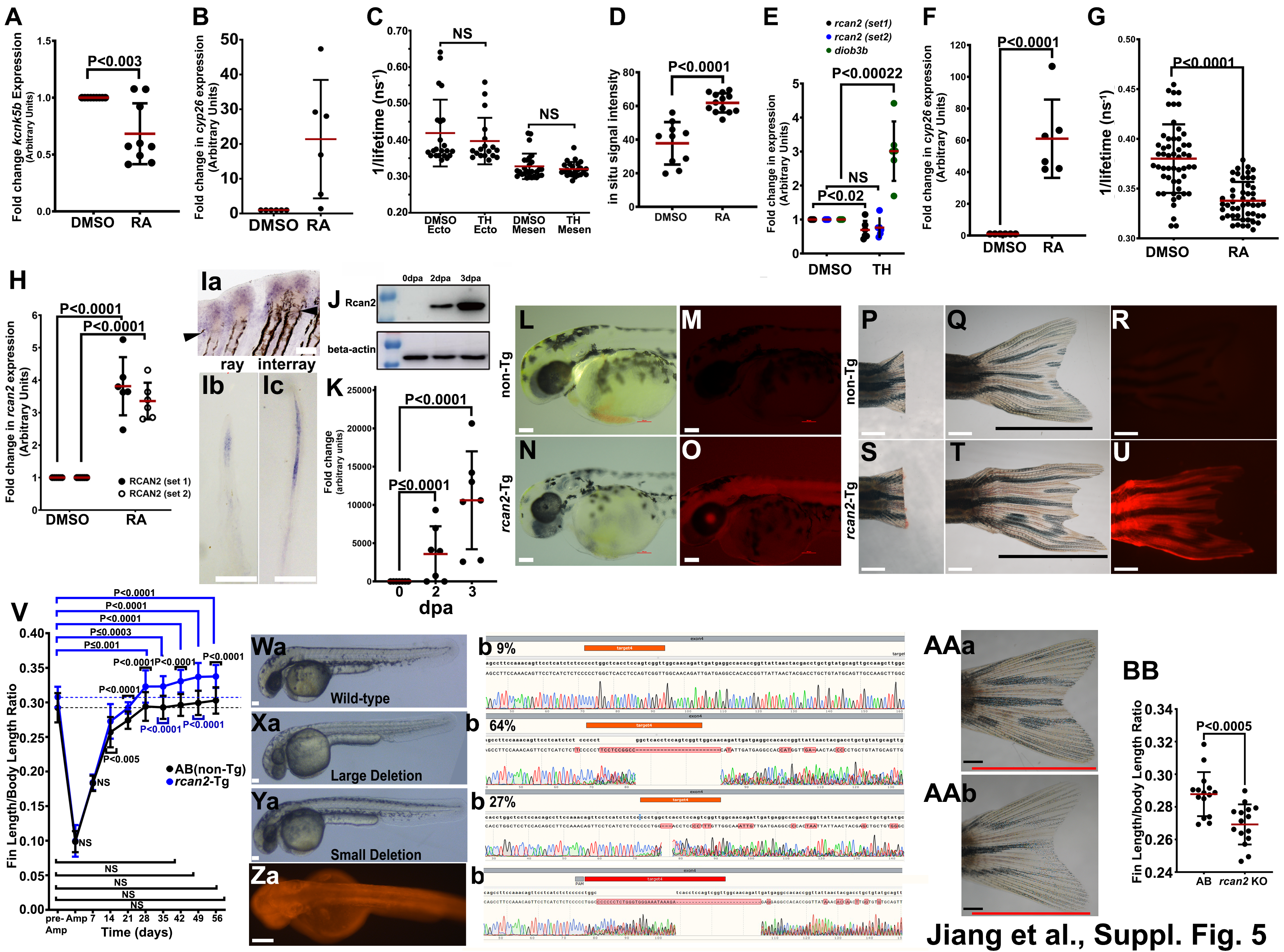

Supplement: S5 Fig — (A) qRT-PCR measurements of the transcription of kcnk5b in the developing pectoral fin bud with and without retinoic acid stimulation. (B) qRT-PCR measurements of cyp26a expression in pectoral fin buds with or without 200 nM retinoic acid treatment, a gene known to be induced by retinoic acid treatment. (C) FLIM measurements from Ectoderm (Ecto) and Mesenchyme (Mesen) cells of buds at 32 hpf treated either with DMSO or 200 nM thyroid hormone for 6 h. (D) In situ staining intensity measurements from the in situs of rcan2 expression in the embryonic fin buds after DSMO or RA treatment. (E) qRT-PCR for expression of rcan2 and dio3b in adult caudal fin after treatment with DMSO or 500 nM thyroid hormone for 24 h. dio3b is a gene known to be up-regulated by thyroid hormone stimulation in the adult caudal fin. (F) RT-PCR for expression of cyp26a treated either with DMSO or 100 nM RA for 6 h in adult caudal fin. (G) FLIM measurements of intracellular K+ levels in adult caudal fin cells after 6 h treated either with DMSO or 100 nM RA. (H) qRT-PCR measurements using 2 different primer sets for rcan2 in adult caudal fins of indicated treatment groups. (I) Whole-mount in situ hybridization of a caudal fin 3 day post amputation shows rcan2 expression in the distal blastemal but absent from the proximal blastema (a). Arrowheads indicate amputation plane. Cryo cross sections through the 3 day post amputation, distal tip of a regenerating adult caudal fin in the ray (b) and interray tissues (c) after in situ hybridization for rcan2. The blue color indicates rcan2 expression. (J) Representative western blot for Rcan2 and beta-actin proteins from lysates of regenerating adult caudal fins at the indicated days post amputation (dpa). (K) Measurements of Rcan2 protein expression after standardization to beta-actin expression at the indicated time points. (L) Representative brightfield image of non-transgenic sibling 6 h after heat-shock stimulation. (M) Representative mCherry fluoresce [file pbio.3002565.s019.tif]

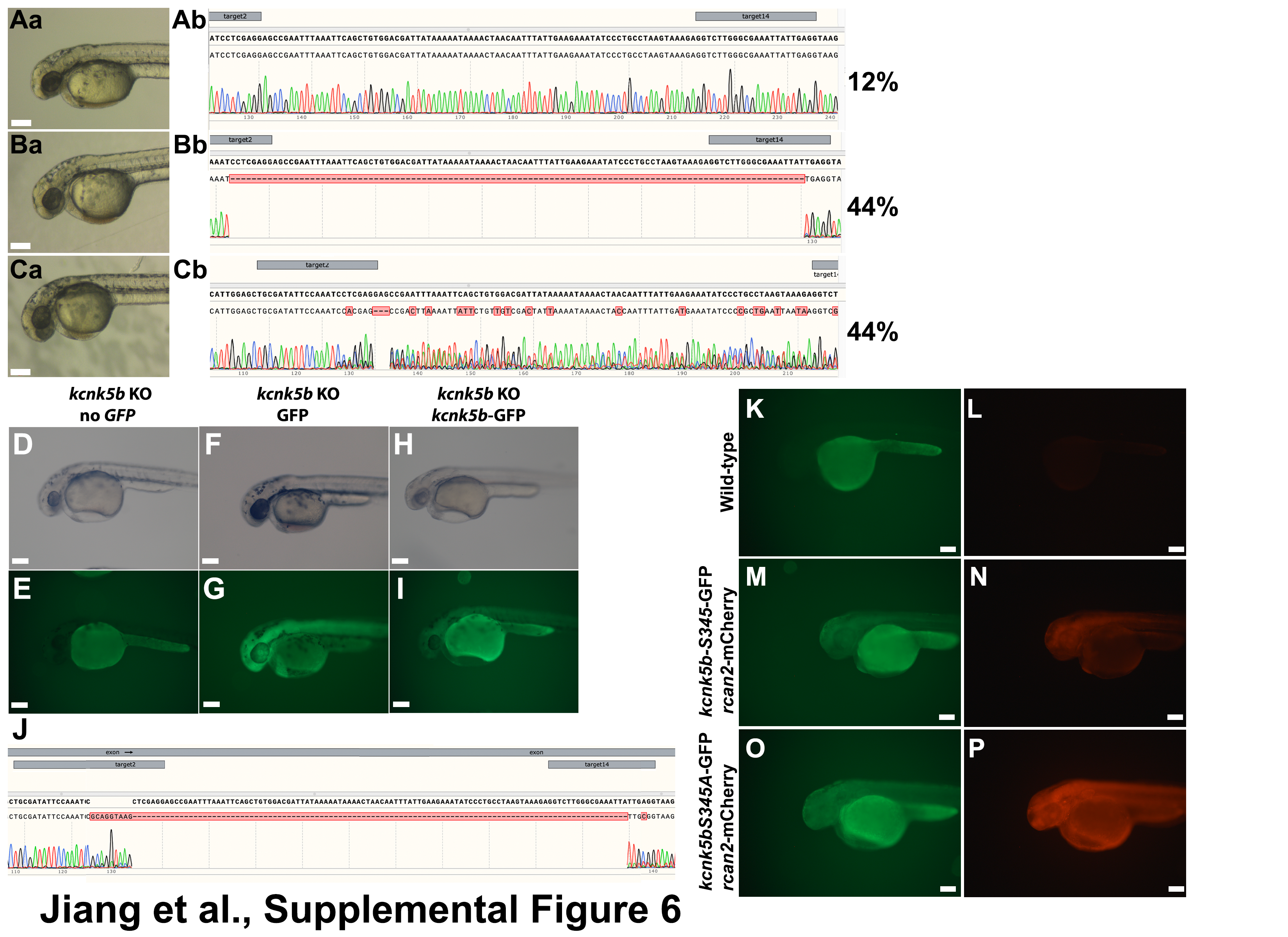

Supplement: S6 Fig — (A) Wild-type 48 hours post fertilization (hpf) larva (a), and sequence of kcnk5b gene in exon 1 (b). Of 100 embryos targeted, 12% do not show gene defects. (B) 48 hpf embryo (a) harboring a large deletion in the kcnk5b exon 1 (b), and 44% harbor large deletions. (C) 48 hpf embryo (a) harboring small deletions and sequence changes (b), and 44% harbor such small deletions. The sgRNA target sites are indicated by the gray box labeled “target.” (D, E) Representative embryo with kcnk5b CRISPR knockout (kcnk5b KO) brightfield (D) and lack of green fluorescence (E) of CRISPR targeted embryos. (F, G) Representative embryo kcnk5b KO brightfield (F) and GFP from control GFP mRNA. (H, I) Representative embryo kcnk5b KO rescued with expression of kcnk5b*-GFP mRNA, brightfield (H) and green fluorescence (I). kcnk5b*-GFP mRNA has mutated wobble-position nucleotides of codons to impair interaction with the sgRNA to continue sgRNA-mediated disruption of the kcnk5b alleles while maintaining the integrity of the transgenic Kcnk5b protein. (J) Representative disrupted kcnk5b allele sequence after CRISPR-targeting and kcnk5b*-GFP-expressing embryo. (K, L) Wild-type, uninjected embryo for GFP (K) and absence of mCherry (L) fluorescence. (M, N) Representative transgenic embryo for kcnk5bS345-GFP (M) and rcan2-mCherry (N) mRNAs. (O, P) Representative transgenic embryo for kcnk5bS345A-GFP mutant (O) and rcan2-mCherry (P) mRNAs. Scale bars equal 100 μm (A–C, D–I, K–P). (TIF) [file pbio.3002565.s020.tif]

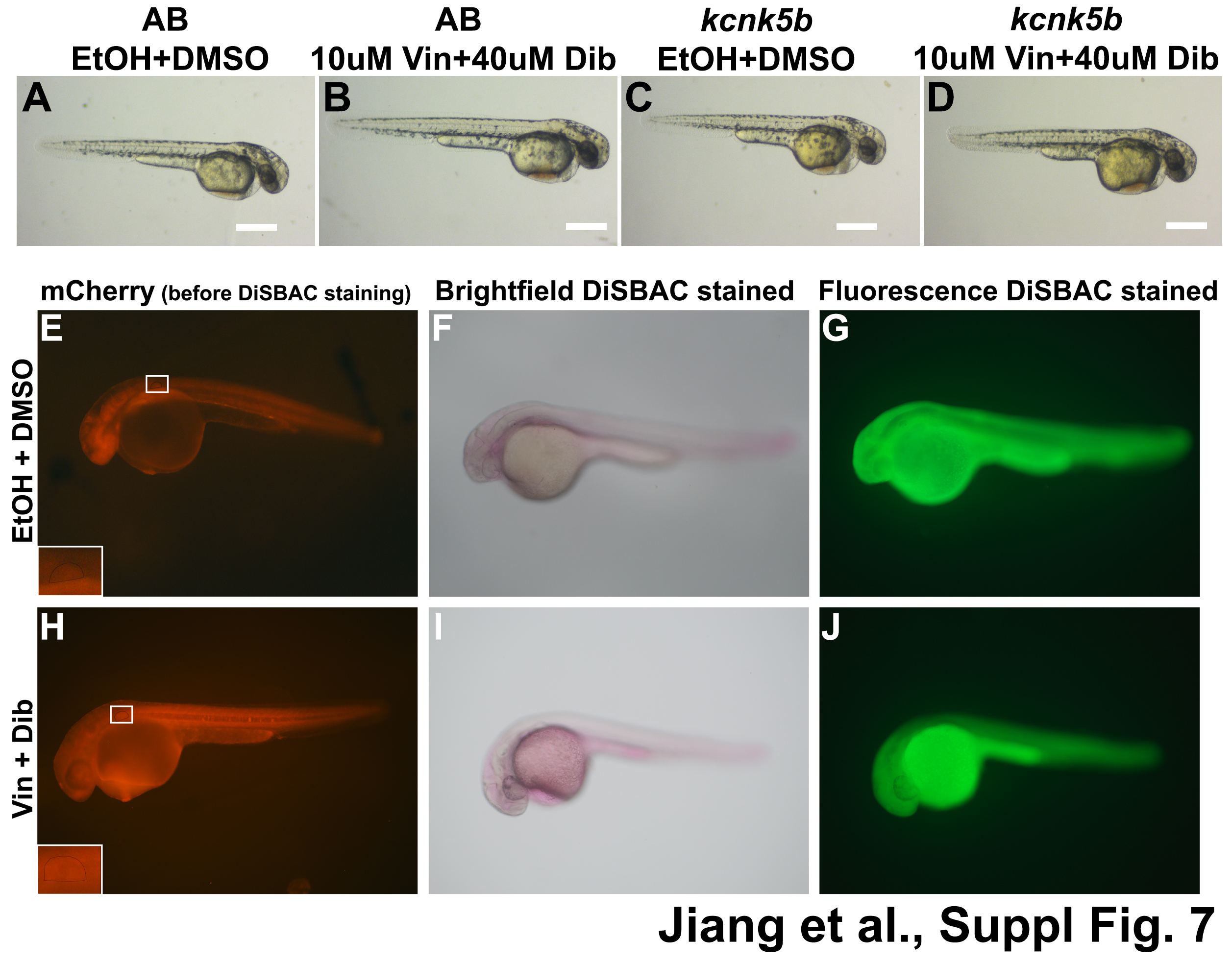

Supplement: S7 Fig — (A) Representative image of a 48 hpf AB non-transgenic fish after 1 heat shock at 32 hpf and treated only with the solvents ethanol (EtOH) and DSMO for 12 h starting at 36 hpf. (B) Representative image of 48 hpf AB non-transgenic fish after 1 heat shock at 32 hpf and treated with 10 μm Vinpocetine (Vin) and 40 μm Dibuciane (Dib) for 12 h starting at 36 hpf. (C) Representative image of a 48 hpf Tg[hsp70:kcnk5b-GFP] fish heat-shocked once at 32 hpf to induce expression of kcnk5b-GFP and treated for 12 h with the solvents EtOH and DMSO at 36 hpf. (D) Representative image of a 48 hpf Tg[hsp70:kcnk5b-GFP] fish heat-shocked once at 32 hpf to induce expression of kcnk5b-GFP and treated with 10 μm Vinpocetine (Vin) and 40 μm Dibuciane (Dib) for 12 h starting at 36 hpf. (E–J) Embryos incubated in 10 μm DiSBAC2(3) dye for 3 h show dye penetration in the embryos. mCherry fluorescence of embryo treated with EtOH and DMSO before DiSBAC2(3) incubation (E). Brighfield image of embryo treated with EtOH and DMSO after DiSBAC2(3) incubation (F). Fluorescence of DiSBAC2(3) in embryo treated with 10 μm Vin and 40 μm Dib before DiSBAC incubation (I). mCherry fluorescence of embryo treated with Vin and Dib before DiSBAC2(3) incubation (H). Brighfield image of embryo treated with Vin and Dib after DiSBAC2(3) incubation (I). Fluorescence of DiSBAC2(3) in embryo treated with Vin and Dib after DiSBAC2(3) incubation (J). (TIF) [file pbio.3002565.s021.tif]

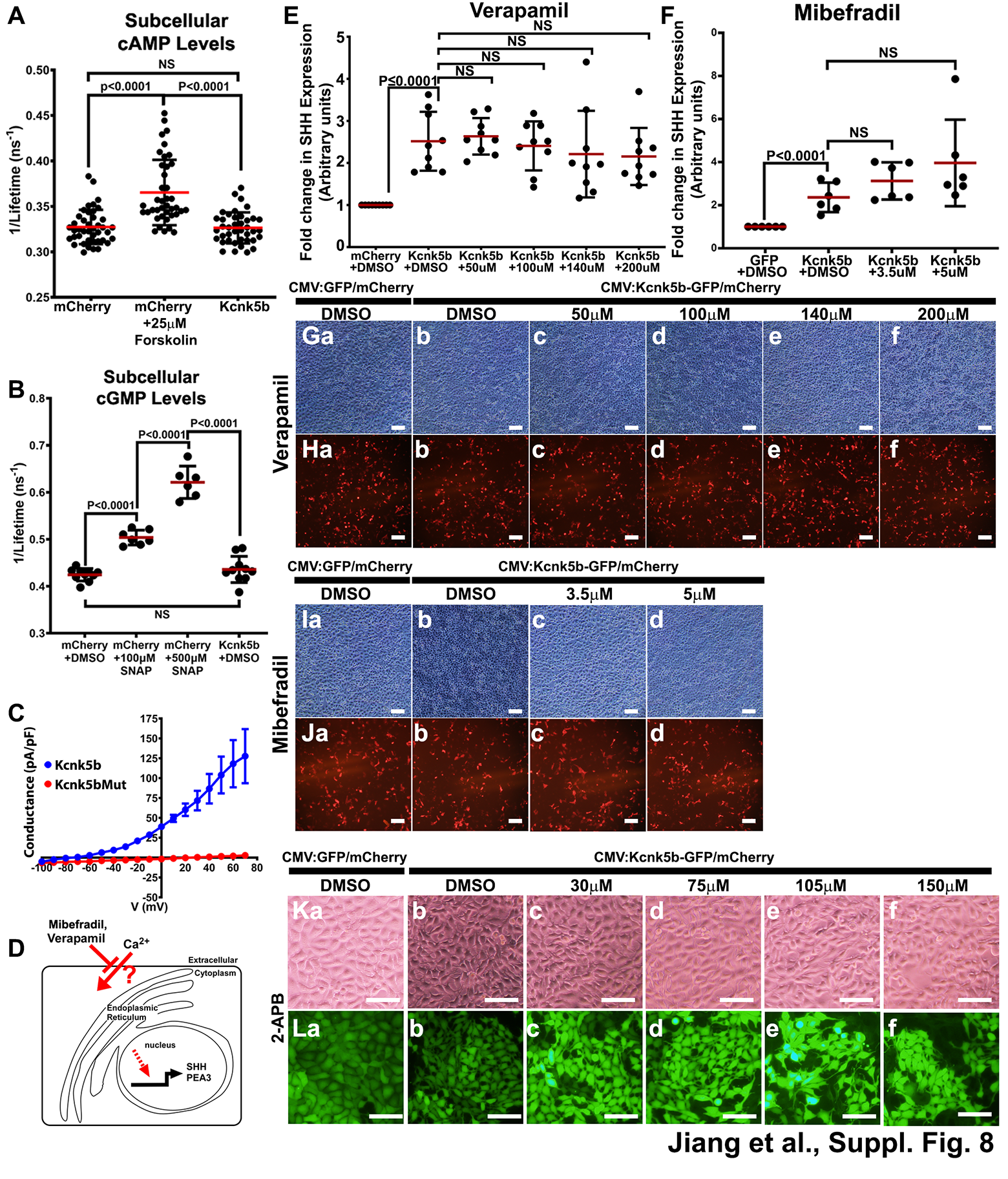

Supplement: S8 Fig — (A) FLIM-FRET measurements of intracellular cAMP levels. Forskolin used as positive control for cAMP production. (B) FLIM-FRET measurements of intracellular cGMP levels. SNAP used as positive control for cGMP production. (C) Patch-clamp experiment measuring the K+ leak from HEK293T cells transfected either with Kcnk5b-GFP (blue) or a mutated Kcnk5bMut-GFP (red) that displayed almost no channel activity. (D) Diagram for T- and L-type Ca2+ channel inhibition by verapamil or mibefradil. (E) qRT-PCR of SHH expression in HEK293 cells after treatment with verapamil at the indicated concentrations. (F) qRT-PCR of SHH expression in HEK293 cells after treatment with Mibefradil at the indicated concentrations. (G) Brightfield images of HEK293 cells transfected with CMV-mCherry and treated with DMSO (a) or transfected with CMV-Kcnk5b-mCherry and treated with DMSO (b) or with verapamil, a L-/T-channel inhibitor, at 50 μm (c), 100 μm (d), 140 μm (e), 200 μm (f). (H) Fluorescence of HEK293 cells transfected with CMV-mCherry and treated with DMSO (a), or transfected with CMV-Kcnk5b-mCherry and treated with DMSO (b) or with verapamil at 50 μm (c), 100 μm (d), 140 μm (e), 200 μm (f). (I) Brightfield images of HEK293 cells transfected with CMV-mCherry and treated with DMSO (a) or transfected with CMV-Kcnk5-mCherry and treated with DMSO (b) or with Mibefradil, a L-/T-channel inhibitor, at 3.5 μm (c), 5 μm (d). (J) Fluorescent images of HEK293 transfected with CMV-mCherry and treated with DMSO (a) or transfected with CMV-Kcnk5b-mCherry and treated with DMSO (b) or with Mibefradil at 3.5 μm (c) 5 μm (d). (K) Brightfield images of HEK293 cells transfected with CMV-GFP and treated with DMSO (a) or transfected with CMV-Kcnk5b-GFP and treated with DMSO (b) or with the IP3 receptor inhibitor 2-APB at 30 μm (c), 75 μm (d), 105 μm (e), 150 μm (f). (L) Fluorescence images of HEK293 cells transfected with CMV-GFP and treated with DMSO (a) or transfected with CMV-Kcnk5b-GFP and treated with DMSO [file pbio.3002565.s022.tif]

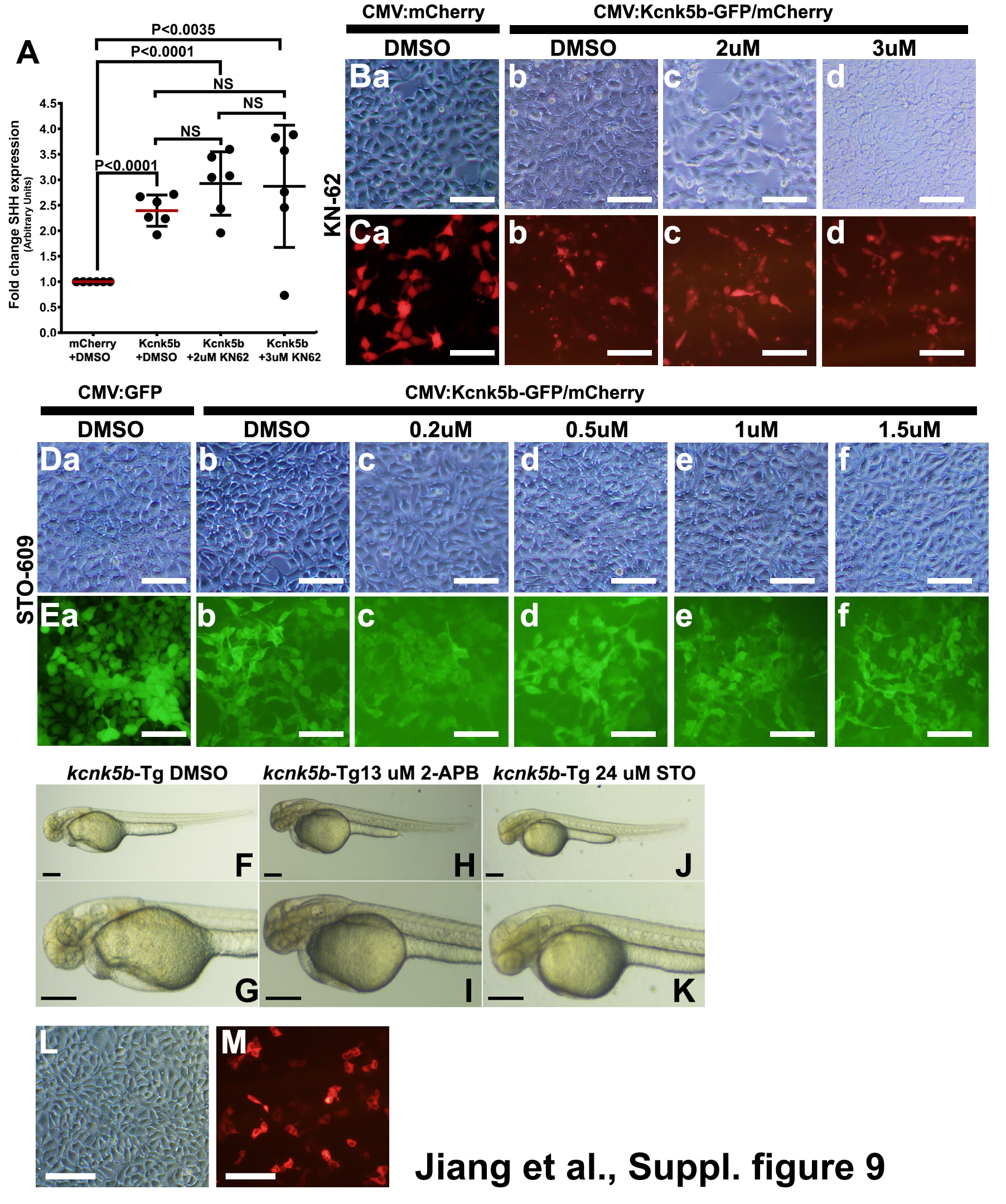

Supplement: S9 Fig — (A) qRT-PCR for SHH in HEK293 cells transfected either with GFP or Kcnk5b-GFP and treated with DMSO or the CaMKII,IV inhibitor KN-62 at the indicated concentrations. (B) Brightfield images of HEK293 cells transfected with CMV-mCherry and treated with DMSO (a) or transfected with CMV-Kcnk5b-mCherry and treated with DMSO (b) or treated with KN-62 an inhibitor for CaMKII and CaMKIV at 2 μm (c) and 3 μm (d). (C) Fluorescence images of HEK293 cells transfected with CMV-mCherry and treated with DMSO (a) or transfected with CMV-Kcnk5b-mCherry and treated with DMSO (b) or treated with KN-62 an inhibitor for CaMKII and CaMKIV at 2 μm (c) and 3 μm (d). (D) Brightfield images of cells treated transfected with CMV-GFP and treated with DSMO (a) or transfected with CMV-Kcnk5b-GFP and treated with DSMO (b) or STO-609 an inhibitor of CaMKK at 0.2 μm (c), 0.5 μm (d), 1 μm (e), 1.5 μm (f). (E) Fluorescent images of cells treated transfected with CMV-GFP and treated with DSMO (a) or transfected with CMV-Kcnk5b-GFP and treated with DSMO (b) or STO-609 an inhibitor of CaMKK at 0.2 μm (c), 0.5 μm (d), 1 μm (e), 1.5 μm (f). (F) Representative 48 hpf kcnk5b transgenic Tg[hsp70:kcnk5b-GFP] embryo 12 h post heat shock and 6 h treatment in solvent concentration of DMSO. (G) Enlarge view of kcnk5b-Tg embryo (F) shows some heart edema associated with heat-shock-induced expression of kcnk5b. (H) Representative 48 hpf kcnk5b transgenic embryo 12 h post heat shock and 4 h treatment in the IP3R inhibitor 13 μm 2-APB. (I) Enlarge view of kcnk5b transgenic embryo in (H). (J) Representative 48 hpf kcnk5b transgenic embryo 12 h post heat shock and 6 h treatment in 24 μm STO. (K) Enlarge view of kcnk5b transgenic embryo in (J). (L, M) Cells transfected with CMV-camkk1b-mCherry in representative brightfield (L) and fluorescence (M) images. Each experiment was repeated at least 3 times (N ≥ 3) and each repeat contained duplicate or triplicate samples. P values represent statistical analysis by Student’s t [file pbio.3002565.s023.tif]
